# ﻿Resurrection of the genus *Homostylium* Nees for the former *Aster* ser. *Albescentes* Y.Ling (Astereae, Asteraceae), with an updated generic synopsis

**DOI:** 10.3897/phytokeys.259.155606

**Published:** 2025-06-17

**Authors:** Xinyu Chen, Zhixi Fu

**Affiliations:** 1 Key Laboratory of Land Resources Evaluation and Monitoring in Southwest (Sichuan Normal University), Ministry of Education, Chengdu 610066, China Sichuan Normal University Chengdu China; 2 College of Life Science, Sichuan Normal University, Chengdu 610101, China Institute of Botany, Chinese Academy of Sciences Beijing China; 3 Sustainable Development Research Center of Resources and Environment of Western Sichuan, Sichuan Normal University, Chengdu 610101, China Sichuan Normal University Chengdu China; 4 State Key Laboratory of Plant Diversity and Specialty Crops, Institute of Botany, Chinese Academy of Sciences, Beijing 100093, China Institute of Botany, Chinese Academy of Sciences Beijing China

**Keywords:** *Aster*, Asteraceae, Astereae, classification, *Homostylium*, taxonomy

## Abstract

Recent molecular phylogenetic studies have demonstrated extensive paraphyly of Asterser.Albescentes Y.Ling. This woody-based shrubby group is both morphologically and molecularly distinct from the genus *Aster* L. The series is characterized by its shrubby habit with multiple branches, cauline leaves with revolute or flat margins, radiate capitula, keeled phyllaries, and multinerved achenes. We redefine a more natural circumscription of this series by reinstating *Homostylium* Nees with nomenclatural priority. A taxonomic synopsis of this series is presented herein. *Homostylium* is recognized and described as a distinct genus (Astereae, Asteraceae) to accommodate 7 shrubby species (6 endemic to China) formerly placed in Asterser.Albescentes. A total of 19 new combinations are proposed, e.g., *Homostyliumalbescens* (DC.) Z.X.Fu, **comb. nov.** (incl. 10 varieties), *Homostyliumargyropholium* (Hand.-Mazz.) Z.X.Fu, **comb. nov.** (incl. 2 varieties), *Homostyliumfulgidulum* (Grierson) Z.X.Fu, **comb. nov.**, *Homostyliumhypoleucum* (Hand.-Mazz.) Z.X.Fu, **comb. nov.**, *Homostyliumlavandulifolium* (Hand.-Mazz.) Z.X.Fu, **comb. nov.**, *Homostyliummotuoense* (Y.L.Chen) Z.X.Fu, **comb. nov.**, and *Homostyliumpolium* (C.K.Schneid.) Z.X.Fu, **comb. nov.** Formal typifications, synonyms, illustrations, distribution maps, and an identification key to the species of *Homostylium* are also provided herein.

## ﻿Introduction

The genus *Aster* L., as currently circumscribed, comprises around 152–180 species, primarily distributed across Eurasia (Nesom 1994; Nesom and Robinson 2007; Chen et al. 2011; Chen et al. 2024). It is a large genus within the tribe Astereae (Asteraceae), with approx. 123 species (82 species endemic) distributed in China (Chen et al. 2011). The genus is characterized by its herbs habit (rarely shrubs or subshrubs), white or purple ray florets, and typically compressed obovoid achenes with 2(-6) ribs (Nesom 1994; Anderberg et al. 2007; Chen et al. 2011). For a long time, *Aster* was defined in a broad sense, including more than 300 of morphologically similar but phylogenetically distant species distributed across Eurasia, Southeastern Africa, and North America (Nesom 1994). The taxonomic revisions, particularly by Nesom (1994), excluded the North American (New World) asters (except *Asteralpinus* L.), restricting *Aster* species to Old World. Brouillet et al. (2009) further removed African asters from the genus. Chen et al. (2011) acknowledged the taxonomic treatment of Nesom (1994) and Brouillet et al. (2009). Chen et al. (2011) subsumed the Asian members of related genera under *Aster* such as *Doellingeria* Ness (A.sect.Teretiachaenium), *Kalimeris* Cass. (A.sect.Asteromoea), *Heteropappus* Less. (A.sect.Pseudocalimeris), and *Miyamayomena* Kitam. and *Rhynchospermum* Reinw. (both included within A.sect.Aster). Accordingly, Chen et al. (2011) divided *Aster* into seven subgeneric groups: Incertae sedis group, A.sect.Teretiachaenium Kitamura, A.sect.Ageratoides (Kitamura) G.L.Nesom, A.sect.Asteromoea (Blume) Makino, A.sect.Pseudocalimeris Kitamura, A.sect.Aster, and A.sect.Alpigeni Nees. However, *Aster* as currently defined is both paraphyletic and polyphyletic even after the exclusion of North American and African asters (Li et al. 2012; Jafari et al. 2015). The precise circumscription of *Aster* remains unresolved and has been inconsistently interpreted across studies.

The introduction of molecular data has provided new evidence for the taxonomic study of *Aster* (Li et al. 2012; Jafari et al. 2015; Korolyuk et al. 2015; Nesom 2020a, 2020b; Chen et al. 2024). Li et al. (2012) proposed a new circumscription for *Aster* in a strict sense by including ten related groups and excluding Asterser.Hersileoides Y.Ling, Asterser.Albescentes Y.Ling, and Alpine *Aster*, etc. Jafari et al. (2015) confirmed the hypothesis of Li et al. (2012) that *Aster* is paraphyletic and polyphyletic. Korolyuk et al. (2015) identified three monophyletic groups within *Aster*, including “typical Eurasian asters”, “Heteropappus group”, and “Astherotamnus group”. They also assumed that the groups of “Heteropappus” and “Astherotamnus” should be treated as independent genus within Asterinae. Nesom (2020a, 2020b) subsequently delimited *Aster* in its strictest sense, retaining only the “Amellus group” and the “Kalimeris group” within its circumscription. The “Kalimeris group” included *Kalimeris* Cass., *Heteropappus* Less, *Miyamayomena* Kitam., *Sheareria* S.Moore, and the “ageratoides” group. The “Asteramellus group” included *Asteramellus* L. (the type of the genus), *A.alpinus*, *Rhynchospermum* Reinw., *Turczaninovia* DC., etc. Nesom (2020a, 2020c, 2020d, 2020e, 2020f, 2020g) proposed multiple new combinations segregated from *Aster*, namely *Geothamnus*, *Griersonia*, *Tibetiodes*, *Yonglingia*, etc. These newly recognized genera were divided into three branches (“Asterothamnus”, “Psychrogeton”, and “Hersileoides”) and two groups with unsettled phylogenetic placement (“Chlamydites” and “Iteroloba”). Chen et al. (2024) analyzed 25 chloroplast genomes of *Aster* and divided it into four distinct clades: core *Aster* (Clade A), Asterser.Albescentes (Clade B), Asterser.Hersileoides (Clade C), and Alpine *Aster* (Clade D). They argued that the genus should be restricted to include core *Aster* consistent with *Aster* sensu Li et al. (2012), with the same exclusion of clades B–D as Li et al. (2012), but in disagreement of Nesom (2020a, 2020b). This suggests that further evidence may lead to a comprehensive redefinition of *Aster* by merging related genera or segregating certain groups as distinctly new genera, such as Asterser.Albescentes.

In the Flora of China (Chen et al. 2011), Asterser.Albescentes was recognized as comprising six species, five of which are endemic to southwest China. In addition to these six species, Chen (1988) discovered and described *Astermotuoensis* Y.L.Chen, a new species endemic to Mêdog County, Tibet, China. Based on its original description and specimens cited, the species was identified as a new member of Asterser.Albescentes. Thereby, this series currently includes seven species, characterized by their shrubby habit with multiple branches, cauline leaves with revolute or flat margins, radiate capitula, keeled phyllaries, and multinerved achenes. The species of this series thrive in the forest understory, significantly contributing to soil-water conservation and ecological sustainability. However, there are various taxonomic treatments of Asterser.Albescentes based on morphological and phylogenetic studies. Asterser.Albescentes was initially established by Ling et al. (1985) within the genus *Aster* in the Flora Reipublicae Popularis Sinicae, with *Asteralbescens* (DC.) Hand.-Mazz designated as its type species. They placed nearly all shrubby species of asters into this series. However, Nesom (1994) noted that this series was isolated among Old World asters and concluded its segregation as a separate genus within Astereae. Chen et al. (2011) yet placed it in the “Incertae sedis group” within *Aster* due to insufficient phylogenetic data to place it within the tribe Astereae. This group included almost all shrubby species of *Aster*, consisting of Asterser.Albescentes and Asterser.Hersileoides (*Asterhersileoides* C.K.Schneid., and *Asternitidus* C.C.Chang). Additionally, Chen et al. (2011) suggested that this series could be considered as a section within *Aster*. Nesom (2020g) then proposed a new combination, namely *Sinosidus*, to accommodate this series, based on studies of Li et al. (2012), Zhang et al. (2015), Fu et al. (2019), and Nesom (2020a, 2020b).

Recent molecular phylogenetic analyses (Li et al. 2012; Jafari et al. 2015; Korolyuk et al. 2015; Zhang et al. 2015, 2019; Fu et al. 2016; Fu et al. 2019; Nesom 2020a, 2020b, 2020g; Chen et al. 2024) have consistently revealed the presence of multiple distinct lineages within *Aster*, including Asterser.Albescentes. The phylogenetic tree of *Aster* (Li et al. 2012), based on internal transcribed spacer, indicated that three species of Asterser.Albescentes form a monophyletic clade distantly separated from core *Aster* clade. Compared to *Asterargyropholis* Hand.-Mazz., *Asterlavandulifolius* Hand.-Mazz. was found to be more closely related to *Asteralbescens* (DC.) Koehne. To better understand the relationship of this series within *Aster* or Astereae, a broader sampling across the extensive evolutionary and taxonomic landscape was needed. Zhang et al. (2015, 2019) proposed a large-scale phylogeny of *Aster* and its 19 related genera (involving 3 species of Asterser.Albescentes) based on combined data (ITS, ETS, and *trnL-F*). They confirmed the distant relationship between Asterser.Albescentes and core *Aster*. Their analysis also revealed that the series forms a well-supported monophyletic group. Fu et al. (2019) further confirmed the monophyly of this series (involving 5 species) based on phylogenetic tree inferred from combined data (ITS and ETS). *Asterfulgidulus* Grierson was identified as the basal species to the other four, with *A.argyropholis*, *Asterpolius* C.K.Schneid., and *A.lavandulifolius* forming a clade separate from *A.albescens*. Compared to *A.lavandulifolius*, *A.polius* was more closely related to *A.argyropholis*. Chen et al. (2024) found that five species of this series form a monophyletic clade with high support, suggesting that it warrants recognition as separate genera. Their analysis also revealed that *A.argyropholis*, *A.albescens*, and *A.lavandulifolius* clustered into a separate clade from *A.polius*, with *Asterhypoleucus* Hand.-Mazz. as the basal species, inconsistent with Fu et al. (2019). This inconsistency may result from frequent cytonuclear discordance (Liu et al. 2022; Zhang et al. 2025). Overall, molecular systematics supports the segregation of Asterser.Albescentes at the generic rank. Although Nesom (2020b, 2020g) had proposed *Sinosidus* within the subtribe Asterinae for this taxon, the relevant literatures indicates that the name was actually not valid. A nomenclatural oversight occurred because Nesom (2020g) overlooked the earlier name *Homostylium* Ness in 1844 with nomenclatural priority for Asterser.Albescentes. Therefore, based on the latest molecular systematic results, an updated taxonomic treatment for this series is urgently required to resolve paraphyletic and polyphyletic issues in *Aster*.

In this study, based on our previous robust molecular systematic evidences and that of other researchers, nineteen new combinations for Asterser.Albescentes (seven species with several varieties) are provided. Formal typification, synonymy, illustrations, distribution maps, and a identification key to the species of the genus are also presented. The main objectives of this study are to (i) resurrect the genus *Homostylium* Nees to accommodate seven shrubby species of *Aster*, (ii) address one of the taxonomic issues of paraphyletic and polyphyletic *Aster* by segregating Asterser.Albescentes, and (iii) provide a taxonomic synopsis of *Homostylium* within the tribe Astereae.

## ﻿Materials and methods

Prof. Dr. Zhi Xi Fu conducted extensive fieldwork across nearly the entire geographical range of *Homostylium* in China (e.g., Shaanxi, Gansu, Sichuan, Xizang, and Yunnan) from August 2011 to November 2016. The collected specimens of *Homostylium* were formally identified by Z. X. Fu based on considerable taxonomic revisions in the Flora Reipublicae Popularis Sinicae (Ling et al. 1985) and Flora of China (Chen et al. 2011). Herbarium study was conducted by Z. X. Fu at 22 herbaria worldwide (AAU, BK, BKF, BM, C, CDBI, E, ECUH, G, GH, HUH, HZU, K, KUN, L, MO, NY, P, PE, QBG, US, and WNU). The specimens of *Homostylium* were retrieved from the Chinese Virtual Herbarium (https://www.cvh.ac.cn/), the full version of the subscription-based JSTOR Global Plants database (https://plants.jstor.org/), and other well-digitized online herbarium databases (e.g., NY) (Thiers 2025). The type specimens examined for the taxa discussed herein were listed in the supplementary file (Suppl. material 1). In addition to the types listed, ca. 1000 specimens of *Homostylium* were examined from 22 herbaria. Specimens without detailed locality information or a clear floristic region assignment were excluded. The distribution maps for the genus *Homostylium* and its 7 species were created and generated using ArcGIS v.10.8.2 (ESRI 2021), primarily based on fieldwork records and literature descriptions.

## ﻿Results

Through literature review, herbarium specimen examinations, and field investigations, we recognized seven species within Asterser.Albescentes. Based on our previous comprehensive molecular phylogeny, external morphology, and achene anatomy, we found this series to be paraphyletic and therefore excluded from *Aster*. In accordance with Articles 11, 41.2, and 42.1 of International Code of Nomenclature for Algae, Fungi, and Plants (Shenzhen Code) (Turland et al. 2018), *Homostylium* Nees holds nomenclatural priority. Therefore, the genus name *Homostylium* was resurrected and adopted to accommodate these shrubby species of the series. We classified this series into 19 new combinations, e.g., *Homostyliumalbescens* (DC.) Z.X.Fu, comb. nov. (incl. 10 varieties), *Homostyliumargyropholis* (Hand.-Mazz.) Z.X.Fu, comb. nov. (incl. 2 variety), *Homostyliumfulgidulus* (Grierson) Z.X.Fu, comb. nov., *Homostyliumhypoleucus* (Hand.-Mazz.) Z.X.Fu, comb. nov., *Homostyliumlavandulifolius* (Hand.-Mazz.) Z.X.Fu, comb. nov., *Homostyliummotuoensis* (Y.L.Chen) Z.X.Fu, comb. nov., and *Homostyliumpolius* (C.K.Schneid.) Z.X.Fu, comb. nov. The selected morphological characters, including plant height, leaves, and inflorescence, of all 7 species within the genus *Homostylium* were also compared in Table 1.

**Table 1.** Comparative morphological characters within the genus *Homostylium* Nees (Data taken from Ling et al. (1985), Chen et al. (2011), and additional herbarium specimens we examined).

Species/ Character	*H.albescens*	*H.argyropholium*	*H.fulgidulum*	*H.hypoleucum*	*H.lavandulifolium*	*H.motuoense*	*H.polium*
Plant height	28–194 cm	93–228 cm	126–192 cm	12–36 cm	48–124 cm	53–166 cm	56–118 cm
Leaves	Narrowly to broadly lanceolate, ovate, elliptic, or oblong-lanceolate, (2-)3–17(-21) × (0.4-)1–3(-12) cm	Elliptic, oblong, or lanceolate-ovate, 1–4 × 0.3–1.6 cm	Ovate, (4-)6–9 × (2-)2.4–4.8 cm	Elliptic, oblanceolate, 0.3–1.7 × 0.19–0.33 cm	Narrowly linear, 1–4(-5.4) × 0.1–0.3(-0.53) cm	Lanceolate-oblong or narrowly oblong, (1-)3–4(-6.3) × 0.7–1.4 cm	Narrowly ovate to elliptic, 1.3–3.7 × 0.4–1.6 cm
Inflorescence	Capitula 6–40, in terminal compound corymbiform synflorescences	Capitula 4–10(-20), in corymbiform synflorescences, terminal on axillary branches	Capitula numerous, in terminal corymbiform synflorescences	Capitula 1–3, terminal on lateral branches	Capitula 3–6 at ends of lateral branches numerous, or numerous in ± densely corymbiform synflorescences, terminal on current-year branches	Capitula numerous, in densely corymbiform synflorescences, terminal on the branch tips or in the upper leaf axils	Capitula 3–10(-20), in corymbiform synflorescences, terminal on current-year lateral branches

### ﻿Taxonomic treatment

#### ﻿ Homostylium

Taxon classificationPlantaeAsteralesAsteraceae

Nees, Del. Sem. Hort. Bot. Vratisl. 3. 1844, emend. Z.X.Fu

72634C37-8E13-5792-9AE1-FB892FA95A7C

= Asterser.Albescentes Y.Ling, Fl. Reipubl. Popularis Sin. 74: 357. 1985.
= Sinosidus G.L.Nesom, Phytoneuron 2020-64: 12. 2020.

##### Type.

*Homostyliumcabulicum* (Lindl.) Nees.

##### Description.

Perennial shrubs. ***Stems*** erect to spreading, profusely branched. ***Leaves*** alternate, leathery, subleathery, herbaceous, or chartaceous, basal leaves withering at flowering, middle cauline leaves ovate, lanceolate to linear; margins entire to remotely apiculate, serrulate, or coarsely serrate, revolute or flat; adaxially glabrous or sparsely appressed pubescent; abaxially closely gray- to white-tomentose or sometimes glabrous, sometimes glandular beneath the tomentum with sessile, resinous glands. ***Capitula*** pedunculate, solitary or in corymbose or paniculate-corymbose inflorescences at branch tips. ***Involucres*** campanulate, cylindrical or obconic, 5–9 mm wide (pressed). ***Phyllaries*** in 3–5-seriate, imbricate, strongly graduate, keeled, oblong, ovate, ovate-lanceolate to lanceolate, glabrous to nearly glabrous, or sparsely short pubescence, tomentum; margins membranaceous or scarious, sometimes ciliate, abaxially herbaceous or sometimes leathery. ***Florets*** heteromorphic, numerous, outer ray florets 1-seriate, pistillate, white, pink, or violet-blue, lamina 3-lobed, glabrous, glabrescent, sparsely filiform-pilose or puberulent; central disc florets numerous, bisexual, yellow, tubes glabrous or sparsely to moderately pilosulous basally or apically, limb 5- lobed, lobes equal or unequal, glabrous or sparsely puberulent, sometimes glandular. ***Anthers*** linear, base obtuse, subauriculate, or caudate, apical appendage lanceolate. ***Styles*** glabrous or distally papillate; bifid in disc florets, branches linear, abaxially glabrous or sparsely papillate, adaxially two stigmatic lines of densely papillate cells from base to appendages; apical sterile appendages triangular or lanceolate, papillate; filiform in ray florets, not bifid. ***Receptacles*** flat or convex to conic, alveolate, sometimes lacerate, epaleate, glabrous. ***Pappus*** persistent, white, straw-colored, or reddish-brown, with numerous subequal strigose bristles, outer layer extremely short strigose bristles. ***Achenes*** white, straw-colored, tan, or reddish brown, compressed, fusiform, cylindrical, oblong, or obovoid, strigillose, (3-)5–8-ribbed, pilose, pubescent, or glandular-pubescent.

##### Distribution.

**Figure 1.**
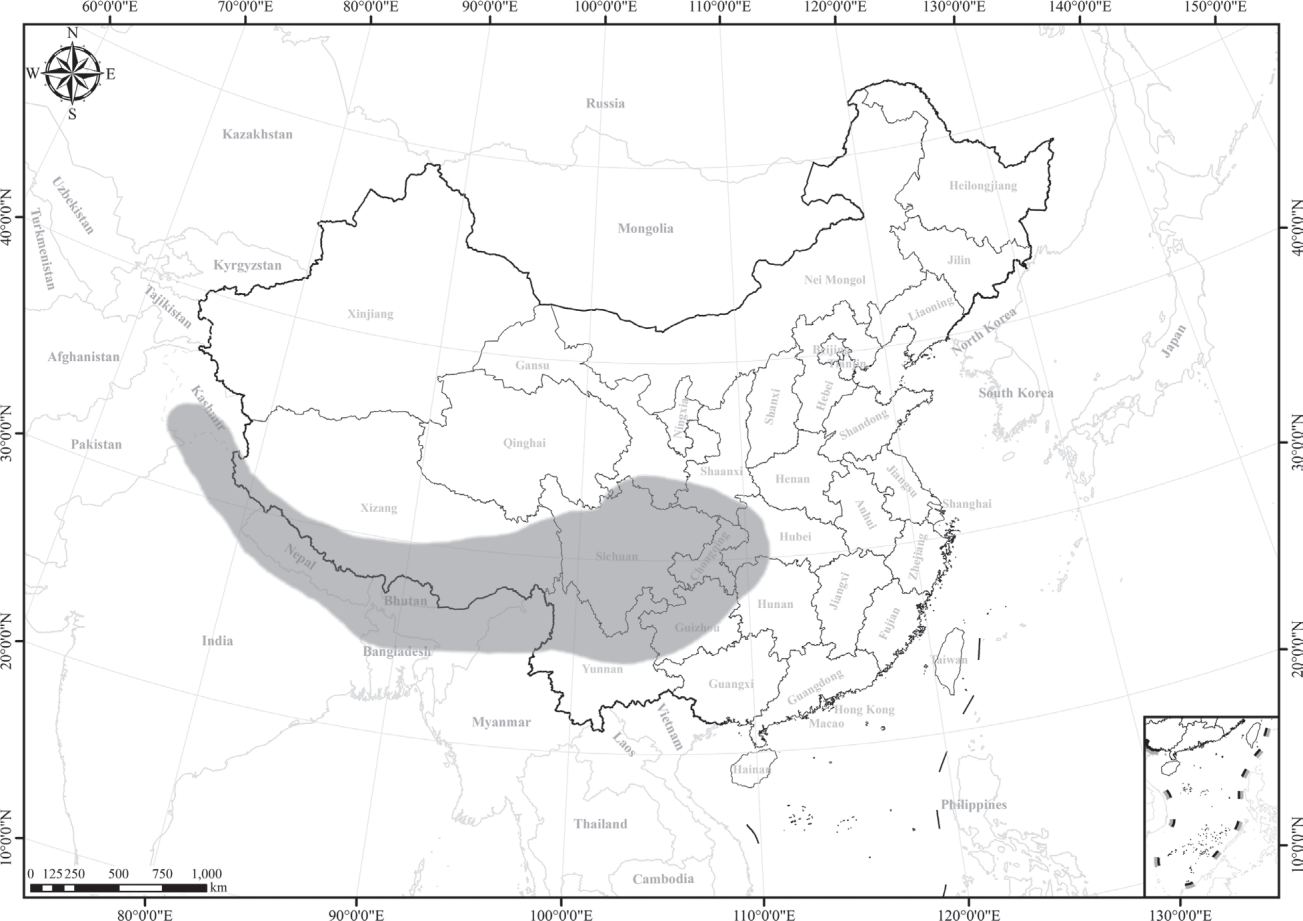
Distribution of the genus *Homostylium* Nees. China is centrally located on the map.

6 of 7 species endemic to SW China. Only *Homostyliumalbescens* distributed widely in SW China and the Himalayan region (Nepal, Bhutan, India, Myanmar, Bangladesh, and Kashmir) (Fig. 1).

##### Habitat.

Alpine and subalpine forests and shrubs, aridly hot river valleys, rock cliffs, and streambanks.

##### Phenology.

Flowering June to October. Fruiting August to November.

##### Etymology.

It is derived from the Greek “*Homo*-”, meaning “same”, and “-*styl*-”, meaning “pillar/style”, combined with the Latin “-*ium*”, a common neuter genus suffix. The genus name probably refers to the uniform style in the florets of *Homostylium* species.

##### Notes.

Among the seven species names in Asterser.Albescentes, the earliest published was *Amphirhapisalbescens* DC. in 1836 by A. P. de Candolle, with no type cited. Jeffrey (1982) later typified the genus *Amphirhapis* DC. with *Amphirhapisheterotricha* DC. However, this species was subsequently treated as a synonym of *Duhaldeaeupatorioides* (DC.) Steetz in the genus *Duhaldea* DC. of the tribe Inuleae (Anderberg 1991). It made the name *Amphirhapis* no longer applicable to accommodate Asterser.Albescentes. The second earliest name was *Homostylium* Nees published in 1844 by Mr. C. G. D. Nees von Esenbeck. *Homostylium* was originally a monotypic genus including only *Homostyliumcabulicum* (Lindl.) Nees. This species is currently treated as a synonym for *Asteralbescens*. The genus is characterized by its shrubby habit, dentate leaves, corymbose inflorescences, cylindrical involucres, imbricately arranged lanceolate-acuminate phyllaries, 1-seriate pistillate pale violet ray florets, bisexual disc florets with 5-lobed limb, and compressed, pubescent, cuneate achenes, as described in its original protologue (https://seedlists.naturalis.nl/content/homostylium-cabulicum-nees). The original morphological characters of *Homostylium* are generally consistent with those of Asterser.Albescentes. However, no type specimen information was provided in the protologue.

In the Flora Reipublicae Popularis Sinicae (Ling et al. 1985), we found that *Homostyliumcabulicum* was cited as a synonym under the name *Asteralbescens*. However, Mr. Rong Ling marked this synonym as “nom. nud. ?”, suggesting it might be a nomen nudum with uncertainty. This was probably due to him not having seen the original literature. After reviewing the original literature published in 1844, we found the monotypic genus *Homostylium* had a detailed description of its characteristics and essential traits, confirming it as validly published and not a nomen nudum. Notably, the name *Homostylium* has not also been used for other taxa. Therefore, we adopted the validly published genus name *Homostylium* with nomenclatural priority to accommodate 7 species of Asterser.Albescentes, in accordance with Articles 11, 41.2, and 42.1 of International Code of Nomenclature for Algae, Fungi, and Plants (Shenzhen Code) (Turland et al. 2018). Initially, *Homostylium* was published as applicable only to *Asteralbescens*. However, to include the remaining six species (*A.lavandulifolius*, *A.argyropholis*, *A.fulgidulus*, *A.motuoensis*, *A.hypoleucus*, and *A.polius*) and its varieties, the genus concept is broadened.

### ﻿Key to the species of *Homostylium* Nees (Astereae, Asteraceae)

**Table:** 

1	Leaves narrowly linear, margin revolute, adaxial papillose; pappus uniseriate	***H* . *lavandulifolium***
–	Leaves ovate, ovate-lanceolate, elliptic, oblong, oblong-lanceolate, or oblanceolate	**2**
2	Capitula in terminal compound corymbiform synflorescences	**3**
–	Capitula in corymbiform synflorescences or solitary at end of lateral branches	**4**
3	Leaves margin entire, adaxially ± glandular; pappus biseriate, dirty white	**5**
–	Leaves magin remotely serrulate to coarsely serrate, teeth mucronulate or entire, adaxially eglandular or rarely sparsely glandular; pappus uniseriate, straw-colored, sometimes purplish	***H.albescens***
4	Leaves margin entire or (1-)2-serrate-spinose, adaxially arachnoid or glabrate, abaxially white tomentose; capitula 1–3 terminal on lateral branches	***H* . *hypoleucum***
–	Leaves margin entire, adaxially verruculose, abaxially white tomentose or arachnoid; capitula 3–10(-20) in corymbiform synflorescences	***H* . *polium***
5	Leaves (4-)6–9 cm long, both surfaces nearly glabrous, abaxially veins reticulate, intercostal glossy	***H* . *fulgidulum***
–	Leaves 1–4 cm long, adaxially moderately scabridulous, abaxially grayish-white arachnoid-tomentose or lanate	**6**
6	Capitula with 15–20 ray florets; involucres campanulate	***H* . *argyropholium***
–	Capitula with 4–6 ray florets; involucres cylindrical or subcylindrical	***H* . *motuoense***

### ﻿New combinations and synonyms in *Homostylium*

#### ﻿1. Homostylium albescens

Taxon classificationPlantaeAsteralesAsteraceae

(DC.) Z.X.Fu comb. nov.

7C265DF3-8F97-589A-AAFD-194305264C99

urn:lsid:ipni.org:names:77363583-1

Figs 2 , 3

≡ Amphirhapisalbescens DC., Prodr. 5: 343. 1836. ≡ Microglossaalbescens (DC.) C.B.Clarke, Compos. Ind. 59. 1876. ≡ Asteralbescens (DC.) Wall. ex Hand.-Mazz., Acti Horti Gothob. 12: 205. 1938. ≡ Sinosidusalbescens (DC.) G.L.Nesom, Phytoneuron 2020-64: 12. 2020. Type: Nepal, Kamaon, Gossain-Than, N. Wallich 2974/84 (lectotype, designated by Nesom (2020g), K 001118307!) (Suppl. material 1: fig. S1).
= Astercabulicus Lindl., Edwards’s Bot. Reg. 29: 62, no. 89. 1843. ≡ Homostyliumcabulicum (Lindl.) Nees, Del. Sem. Hort. Bot. Vratisl. 3. 1844. ≡ Microglossacabulica (Lindl.) C. B. Clarke, Compos. Ind. 57. 1876. Type: Typified by the description.
= Asterignoratus Kunth et Bouche, Ind. Sem. Hort. Berol. 11. 1845. Type: Unknown place, Herb, Schultz Bip. 81 (holotype, P 00691946!) (Suppl. material 1: fig. S2).
= Asterferrugineus Edgew., Trans. Linn. Soc. 20: 64. 1846. Type: India, Carhoul State, Mana. M. P. Edgeworth 16 (holotype, K 000890396!) (Suppl. material 1: fig. S3).
= Microglossasalicifolia Diels, Bot. Jahrb. Syst. 29: 612. 1901. Type: China, Chongqing, Leijiaping, A. V. Rosthorn 136 (holotype, B, not seen).
= Astercavaleriei Vaniot & H.Lév., Bull. Soc. Bot. France 53: 549. 1906. Type: China, Guizhou, Tien-sen-kiao river, Novomeber 1904, J. Cavalerie 1895 (holotype, E, not seen).

##### Type.

Nepal • Kamaon, Gossain-Than. N. Wallich 2974/84 (lectotype, designated by Nesom (2020g), K 001118307!) (Suppl. material 1: fig. S1).

##### Description.

Shrubs, 28–194 cm tall. ***Leaves*** subpapery, narrowly to broadly lanceolate, ovate, elliptic, or oblong-lanceolate, or elliptic, (2–)3–17(–21) × (0.4-)1–3(-12) cm, midvein and pinnate lateral veins abaxially prominent, veins scabrous or glandular-punctate, sparsely to moderately villosulous; margins entire or teeth mucronulate, upper leaves smaller; adaxially glabrous or scabridulous to scabrous, eglandular or rarely sparsely minutely stipitate glandular; abaxially glabrous or sparsely to densely appressed villosulous to tomentulose, eglandular or sometimes sparsely to densely minutely stipitate glandular. ***Capitula*** 6–40 (5–8 mm wide), in terminal compound corymbiform synflorescences at branch tips. ***Peduncles*** slender, 5–11 mm long, bracts subulate. ***Involucres*** obconic to campanulate, 4–7 mm long. ***Phyllaries*** 4–5 seriate, imbricate, unequal, glabrescent to pubescent or tomentose, outer series narrowly lanceolate, ca. 1 mm long, inner series linear-lanceolate, 3.5–4.8 × 0.6–0.8 mm.

**Figure 2.**
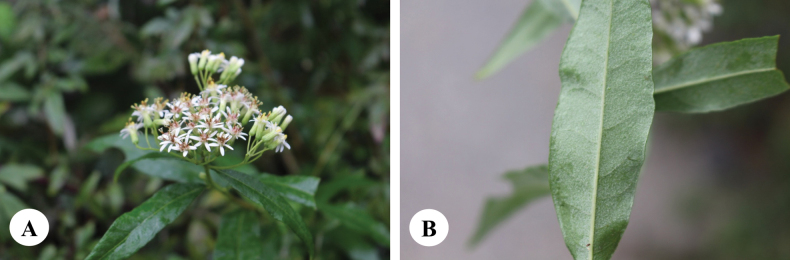
*Homostyliumalbescens* (DC.) Z.X.Fu. **A.** Flowering plant; **B.** Abaxial surface of leaf. Photographed by Z. X. Fu.

##### Distribution.

**Figure 3.**
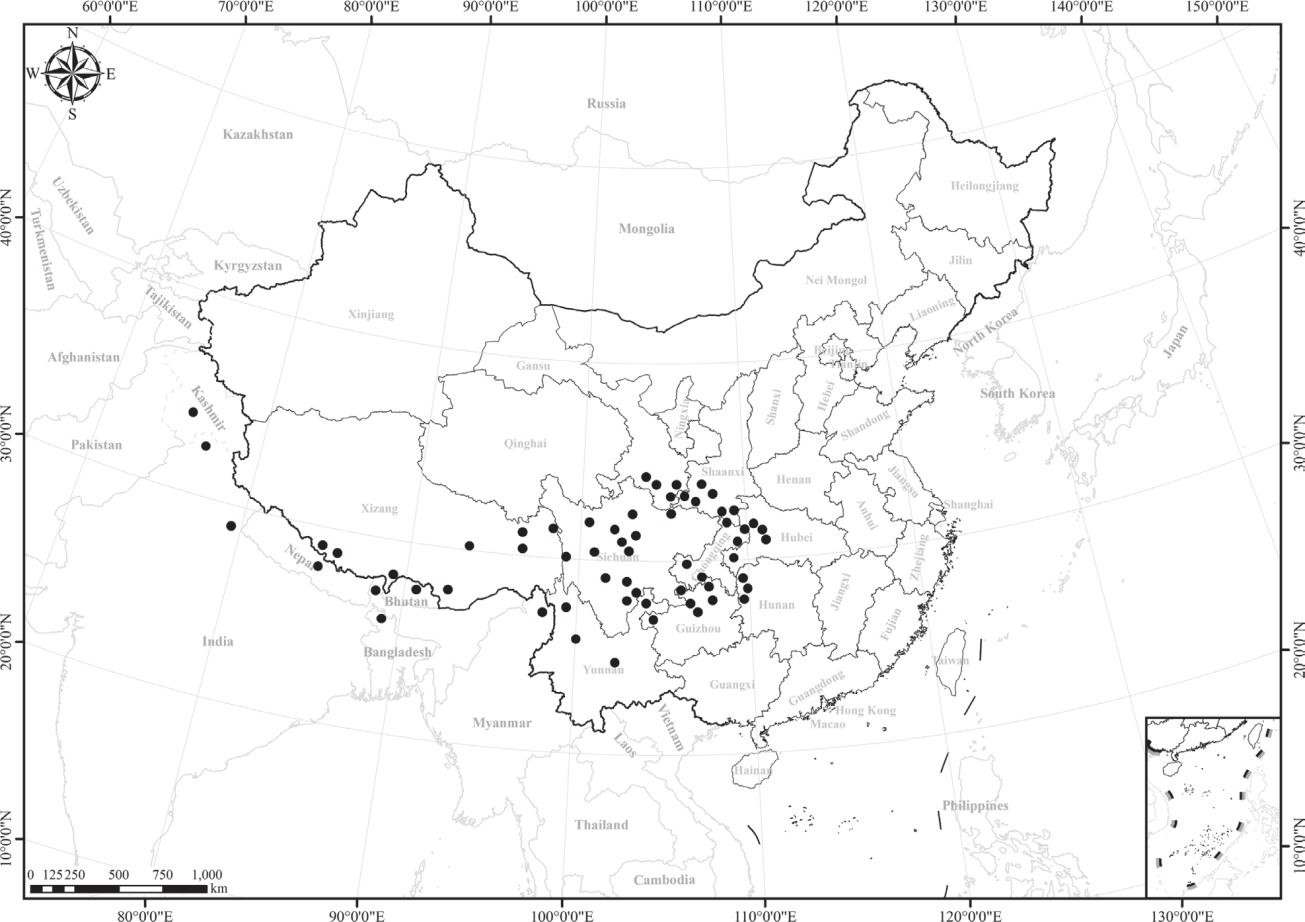
Distribution of *Homostyliumalbescens*. China is centrally located on the map.

Widely distributed in China (S Gansu, N Guizhou, W Hubei, S Shaanxi, Sichuan, S Xizang, N Yunnan), Himalayas region (Bhutan, N India, Kashmir, and Nepal), N Myanmar, and N Bangladesh (Fig. 3).

##### Habitat.

Margins of deciduous or coniferous forests and thickets, open or grazed meadows, shrublands, seepage or damp areas, streamsides, ditch or field margins, disturbed areas and cut or disturbed forests, hills to alpine regions. 500–4100 m.

##### Phenology.

Flowering June to September. Fruiting August to October.

##### Etymology.

It is derived from the Latin “*albesco*-” meaning “to become white” and the neuter suffix “-*ens*”, indicating a present participle or ongoing action. The specific epithet likely refers to the gradual whitening of tomentose on the abaxial surface of the leaf in this species.

##### Notes.

In the Flora Reipublicae Popularis Sinicae (Ling et al. 1985) and Flora of China (Chen et al. 2011), *Homostyliumalbescens* (≡ *Asteralbescens*) was treated under the genus *Aster*. In both Floras, the classification of its intraspecific taxa is nearly consistent. *H.albescens* is a variable complex of intergrading populations of shrubs extending from the western Himalaya to central and south-central China. To facilitate taxonomic work, we have reviewed relevant literature and examined the type specimens and photos of these populations in herbarium collections. Ten varietal names (except type variety) have been assigned to segregate this complex in China, primarily based on pubescence, gland characters, size of the leaves, and pubescence and shape of the involucres (Boufford and Chen 2009). Some of these do not appear to warrant recognition yet. Certain varieties are considered to be closer to, or intermediate between other species. A molecular phylogenetic study is required to further clarify this complex. However, no effort is made herein to assess their evolutionary validity. While these varieties are not formally recognized here, they are still listed below along with their key features and comments. Our current understanding of intraspecific variation in *Homostyliumalbescens* is very limited. Additional collections from the type locality are particularly needed for future study.

#### ﻿1.1. Homostylium albescens var. albescens

Taxon classificationPlantaeAsteralesAsteraceae

F4074BCA-46EA-529F-BCA7-79618FDAE502

##### Diagnosis.

***Leaves*** narrowly to broadly lanceolate, 5–12 × 1–12.5 cm, abaxially gray-white appressed tomentulose, margin flat, entire. ***Phyllaries*** outer ovate, abaxially sparsely hairy or glabrate.

##### Distribution.

Widely distributed in China (Chongqing, Gansu, Guizhou, W Hubei, S Shaanxi, S Sichuan, E and N Xizang, and NW Yunnan), and S and W Himalayan region (Myanmar, India, Bhutan, Nepal, and Kashmir).

##### Habitat.

Understories and shrublands from low to high mountains. 400–4300 m.

##### Specimens examined.

**China** • **Chongqing**: Leijiaping, A. V. Rosthorn 136 (B); Chengkou, T. L. Dai 101444 (PE), 101639 (PE), 101838 (PE), 101982 (PE), 102535 (PE), 103225 (PE 00274528, PE 01831284), 103407 (PE), 103509 (PE), 103650 (PE), 103834 (PE), 103975 (PE), 104192 (PE), 104266 (PE), 104331 (PE), 104428 (PE), 105954 (PE), 106361 (PE), 107079 (PE); Fengjie, H. F. Zhou 26755 (PE), 26884 (PE 00274620, PE 00300444), T. Y. Chang 25759 (PE 00274494, PE 00300443), 25892 (PE), M. Y. Fang 23956 (PE), 24870 (PE), 24979 (PE), Anonymous 24696 (PE); Hechuan, T. H. Tu 5196 (PE); Nanchuan, K. C. Kuan et al. 1409 (PE 00274510, PE 00274511), J. H. Xiong & Z. L. Zhou 92893 (PE), 93620 (PE), G. F. Li 63361 (PE), 64223 (PE), Z. Y. Liu 17947 (PE); Wushan, K. H. Yang 59843 (PE), H. F. Zhou & H. Y. Li 110020 (PE); Wuxi, K. H. Yang 59482 (PE), 65060 (PE), 65481 (PE), K. L. Chu 1898 (PE), 1946 (PE); • **Gansu**: Chengxian, W. Y. Hsia 6127 (PE); Tanchang, Y. M. Yuan 1084 (PE), T. P. Wang 14307 (PE); Kangxian, Z. Y. Zhang 16747 (PE), 17238 (PE), 16429 (PE), Y. S. Lian et al. 96309 (PE); Wenxian, Z. Y. Zhang 13999 (PE), 15534 (PE), 14992 (PE), 15188 (PE), 14317 (PE), Q. X. Li & X. C. Zhao 2376 (PE), 2153 (PE); Zhouqu, Y. Q. He 544 (PE 00274485, PE 00274486); • **Guizhou**: Chishui, Bijie Exped. 1243 (PE); Daozhen, Z. Y. Liu 16233 (PE); Renhuai, X. L. Wang 2189 (PE); Sinan, J. Zhang 4061 (PE); Weining, Bijie Exped. 224 (PE 00274646, PE 00274647, PE 01776696); Xishui, P. C. Tsoong 292 (PE 00274641, PE 00274642, PE 00274643, PE 00274644); Bijie, Bijie Exped. 1546 (PE 00274650, PE 00274651, PE 01776697), 1618 (PE 00274648, PE 00274648, PE 01823525), 1719 (PE 00274652, PE 00274653, PE 01823526), C. W. Wu 1217 (PE); Zunyi, Sichuan–Guizhou Exped. 1055 (PE), 1411 (PE); Tien-sen-kiao river, J. Cavalerie 1895 (E); • **Hubei**: Lichuan, W. C. Cheng & C. T. Hwa 967 (PE), L. Y. Dai & C. H. Qian 822 (PE); Xingshan, Z. D. Chen et al. 961115 (PE); Shennongjia, Sino–Amer. Exped. 1061 (PE); • **Shaanxi**: Hanzhong, J. W. Wang & Z. C. Shi 109 (PE); Lüeyang, C. L. Tang 467 (PE); Nanzheng, K. T. Fu 6161 (PE); Ningqiang, T. N. Liou 11810 (PE 00274549, PE 00274550); Ningshan, H. W. Kung 3305 (PE), P. C. Kuo 1003 (PE); Pingli, P. Y. Li 4992 (PE); Taibai, C. G. Ma et al. 308 (PE 01824224, PE 01824225, PE 01824226); Yangxian, P. C. Kuo 2003 (PE), K. T. Fu 5245 (PE), H. W. Kung 3530 (PE), J. X. Yang 2075 (PE); Ziyang, P. Y. Li 4765 (PE), 4829 (PE), 6326 (PE), H. N. Qin et al. 18407 (PE); • **Sichuan**: Batang, D. E. Boufford et al. 35510 (PE), 35585 (PE), 35616 (PE); Baoxing, C. P’ei 814 (PE), S. S. Chang & Y. X. Ren 7470 (PE); Butuo, Sichuan, Econ. Pl. Exped. 5898 (PE); Dujiangyan, Z. T. Wang et al. 870015 (PE); Ebian, Z. X. Zhao 737 (PE), C. W. Yao 2890 (PE), S. L. Sun 987 (PE); Emei, T. N. Liou 10589 (PE), J. H. Xiong et al. 32869 (PE), W. P. Fang 3182 (PE), K. C. Kuan et al. 1858 (PE 00274512, PE 00274513), 1989 (PE 00274516, PE 00274517), 2508 (PE 00274514, PE 00274515), 2623 (PE 00274508, PE 00274509), K. H. Yang 57013 (PE), 57561 (PE), T. N. Liou & C. Wang 783 (PE 00274522, PE 00274523); Ganluo, Anonymous 4162 (PE 00274518, PE 00274608); Guangyuan, Y. Q. He 1577 (PE), T. N. Liou & C. Wang 207 (PE); Hanyuan, T. P. Wang 9491 (PE); Heishui, X. Li 73404 (PE), 73610 (PE); Jinchuan, X. Li 75360 (PE), G. Z. Zhu & X. Li 75666 (PE); Jiulong, Anonymous 4888 (PE), D. E. Boufford et al. 33040 (PE 01799277, PE 01799278); Kangding, W. K. Hu & C. Ho 11051 (PE), 10641 (PE); Leshan, Z. T. Guan 6327 (PE); Leibo, C. S. Cao 533 (PE), 607 (PE), 635 (PE), 1570 (PE); Lixian, C. S. Cao 1037 (PE); Luhuo, D. E. Boufford et al. 34765 (PE); Mabian, T. T. Yu 4174 (PE 00274507, PE 00274589, PE 00274613); Barkam, Anonymous 23467 (PE); Shimian, C. J. Xie 42525 (PE); Songpan, K. T. Fu 2109 (PE); Tianquan, W. P. Fang 3493 (PE), X. L. Jiang 35067 (PE), 37601 (PE), 38012 (PE), 35143 (PE), X. L. Jiang & J. H. Xiong 37754 (PE), K. L. Chu 4123 (PE), 4116 (PE), W. K. Hu & C. Ho 12002 (PE); Xiaojin, S. S. Chang & Y. X. Ren 6416 (PE); Xinlong, D. E.Boufford et al. 34132 (PE), 36291 (PE), 36269 (PE), 37281 (PE); Ya’an, T. P. Wang 8527 (PE), 8361 (PE), K. C. Kuan et al. 2498 (PE); Yuexi, Sichuan, Econ. Pl. Exped. 3724 (PE); Precise locality unknown, Anonymous 45 (PE), C. P’ei 8062 (PE), H. Smith 13564 (PE), Y. C. Yang 3547 (PE), West China Acad. Sci. 3120 (PE), 4123 (PE), C. L. Wu 12205 (PE); • **Xizang**: Bomê, Z. C. Ni et al. 1428 (PE), H. N. Qin et al. 362 (PE), T. S. Ying & D. Y. Hong 650124 (PE); Qamdo, D. E. Boufford et al. 32367 (PE 01799281, PE 01799282); Cona, Anonymous 75–1622 (PE 00532438, PE 00532439, PE 00532440); Gyirong, Qinghai–Xizang Exped. Vegetation Team 4678 (PE), Qinghai–Xizang Exped. 6966 (PE 00274661, PE 00274669), Jomda, D. E. Boufford et al. 31359 (PE); Riwoqê, D. E. Boufford et al. 32163 (PE); Nyingchi, Z. C. Ni et al. 34 (PE 00274675, PE 00274676), H. N. Qin et al. 193 (PE); Nyalam, Xizang Med. Herb Exped. 1505 (PE 00274666, PE 01532300), Y. T. Zhang & K. Y. Lang 4575 (PE 00274667, PE 00274668), H. N. Qin et al. 744 (PE 01717594, PE 01717595); Yadong, Qinghai–Xizang Exped. 2142 (PE 00274664, PE 00274665), Qinghai–Xizang Supplement Team 750299 (PE 00274671, PE 00274672), Anonymous 75–901 (PE 00532436, PE 00532437); • **Yunnan**: Dali, George Forrest 11670 (PE); Deqin, K. M. Feng 5357 (PE 00274654, PE 00274655), Anonymous 75–766 (PE); Kunming, Z. Y. Liu 19198 (PE); Shangri–la, H. N. Qin et al. 609 (PE); Yiliang, Northeast Yunnan Exped. 943 (PE); Precise locality unknown, T. P. Zhu 235 (PE), George Forrest 11025 (PE), Anonymous 4580 (PE); **India** • **Carhoul**: Mana, M. P. Edgeworth 16 (K 000890396); **Nepal** • **Kamaon**: Gossain-Than, N. Wallich 2974/84 (K 001118307).

#### ﻿1.2. Homostylium albescens var. discolor

Taxon classificationPlantaeAsteralesAsteraceae

(Y.Ling) Z.X.Fu comb. nov.

F471A1F2-132E-5354-A944-A061B65D6CDF

urn:lsid:ipni.org:names:77363584-1

≡ Asteralbescensvar.discolor Y.Ling, Fl. Reipubl. Popularis Sin. 74: 358. 1985. ≡ Sinosidusalbescensvar.discolor (Y.Ling) G.L.Nesom, Phytoneuron 2020-64: 13. 2020. Type: China, Sichuan, Songpan, alt. 2400 m, Roadside, 22 Oct 1937, T. P. Wang 7896 (holotype, PE 00274683!) (Suppl. material 1: fig. S4).

##### Type.

China • Sichuan, Songpan, alt. 2400 m, Roadside, 22 Oct 1937, T. P. Wang 7896 (holotype, PE 00274683!) (Suppl. material 1: fig. S4).

##### Diagnosis.

***Leaves*** elliptic-lanceolate, 2–3.5 × 0.5–1 cm, abaxially white, appressed tomentose, adaxially glabrous. ***Phyllaries*** outer ovate, puberulent or glabrate. This variety is similar to *Homostyliumargyropholium*. It may be intermediate between *H.argyropholium* and *H.polium*.

##### Distribution.

China, Sichuan (Songpan). Ca. 2400 m.

##### Habitat.

Subalpine.

##### Etymology.

The varietal name “*discolor*” is derived from the Latin “*dis*-” meaning “apart” or “opposite”, and “-*color*” meaning “color”. The name likely refers to a color variation (abaxially white leaves) compared to *Homostylium albescens* var. *albescens*.

##### Specimens examined.

**China** • **Sichuan**: Songpan, T. P. Wang 7896 (PE 00274683), T. P. Wang 7924 (PE).

#### ﻿1.3. Homostylium albescens var. glabratum

Taxon classificationPlantaeAsteralesAsteraceae

(Diels) Z.X.Fu comb. nov.

B69C68D6-90AC-5FB6-B4C8-D72FD28BBF02

urn:lsid:ipni.org:names:77363585-1

≡ Asterharrowianusvar.glabratus Diels, Notes Roy. Bot. Gard. Edinburgh 5: 184. 1912. ≡ Asteralbescensvar.glabratus (Diels) Boufford & Y.S.Chen, Harvard Pap. Bot. 14: 43. 2009. ≡ Sinosidusalbescensvar.glabratus (Diels) G.L.Nesom, Phytoneuron 2020-64: 13. 2020. Type: China. Yunnan, Lijiang, shady, rocky situations, side valleys on the eastern flank, Lat. 27'15°N, alt. 9500–11000 ft, July 1906, G. Forrest 2508 (holotype, E 00385621!; isotype, P 00711686!) (Suppl. material 1: figs S5, S6).
= Asteralbescensvar.levissimus Hand.-Mazz., Acta Horti Gothob. 12: 208. 1938. Type: China, Sichuan, Lixian, Zhuokeji (=Drogochi), alt., 3300 m, Anonymous 4544 (holotype, WU, not seen).

##### Type.

China • Yunnan, Lijiang, shady, rocky situations, side valleys on the eastern flank, Lat. 27'15°N, alt. 9500–11000 ft, July 1906, G. Forrest 2508 (holotype, E 00385621!; isotype, P 00711686!) (Suppl. material 1: figs S5, S6).

##### Diagnosis.

***Leaves*** narrowly to broadly lanceolate, 5–12 cm, both surfaces glabrous, sometimes young leaves abaxially sparsely tomentose on midvein only. ***Phyllaries*** outer ovate to lanceolate, glabrous. The leaves are similar in shape to those of Homostyliumalbescensvar.albescens, but are often shorter and narrower.

##### Distribution.

China (Chongqing, Gansu, Xizang, Hubei, W Sichuan, N Yunnan). 800–3000 m.

##### Habitat.

Subalpine.

##### Etymology.

The varietal name “*glabratum*” is derived from the Latin “*glaber*”, meaning “smooth” or “hairless”, combined with the neuter suffix “-*atum*”, which indicates “possession”. The name likely refers to the abaxially smooth, hairless leaf surface of this variety.

##### Specimens examined.

**China** • **Chongqing**: Chengkou, T. L. Dai 103779 (PE); Nanchuan, G. F. Li 64223 (PE), J. H. Xiong & Z. L. Zhou 92453 (PE); Wuxi, G. H. Yang 65505 (PE), • **Gansu**: Kangxian, Z. Y. Zhang 16874 (PE); Wenxian, Y. Q. He 936 (PE), J. X. Yang & Y. Q. He 3380 (PE); • **Hubei**: Xuan’en, H. J. Li 3852 (PE), 4689 (PE); Lichuan, G. X. Fu & Z. S. Zhang 1588 (PE 00274747, PE 00274748, PE 00274749); • **Sichuan**: Baiyu, D. E. Boufford et al. 37202 (PE); Baoxing, T. P. Soong 39045 (PE), K. L. Chu 3120 (PE); Butuo, Sichuan, Econ. Pl. Exped. 5898 (PE); Danba, D. Y. Hong et al. 95061 (PE); Ebian, Anonymous 145 (PE), Anonymous 146 (PE), Heishui, X. Li 74090 (PE), 73288 (PE), 73844 (PE), Jinchuan, X. Li 75077 (PE), 75152 (PE), 75171 (PE), 75231 (PE), 75270 (PE), 75288 (PE), 75387 (PE), 75411 (PE), 75457 (PE), 75460 (PE), 75475 (PE), 75493 (PE), 75557 (PE), 75702 (PE), 75908 (PE), 76223 (PE), 76273 (PE), 76304 (PE), 76531 (PE), 76585 (PE), 76761 (PE), 77993 (PE), 78055 (PE), 78242 (PE), 78362 (PE), 78490 (PE), 78509 (PE 00274889, PE 00274891), 78611 (PE), 78714 (PE 00274757, PE 00274758), G. Z. Zhu & X. Li 75578 (PE), 75629 (PE), 75677 (PE), 75816 (PE), G. Z. Zhu 75052 (PE), Anonymous 9474 (PE), Anonymous 75848 (PE), Kangding, W. G. Hu & X. L. Jiang 36862 (PE), X. L. Jiang 37151 (PE), 37044 (PE), 37010 (PE), C. S. Liu 1058 (PE 00274780, PE 00274797); Lixian, Anonymous 4544 (WU), C. S. Cao 21 (PE), 48 (PE), 149 (PE), 1203 (PE), Z. He & Z. L. Zhou 4216 (PE); Luhuo, Wei L. Chen et al. 7304 (PE); Barkam, X. Li 72379 (PE), 72567 (PE), 71852 (PE), 71944 (PE), X. Li & J. X. Zhou 72668 (PE), J. X. Zhou & X. Li 72729 (PE), Anonymous 23273 (PE); Meigu, Sichuan, Econ. Pl. Exped. 1677 (PE), Anonymous 13096 (PE); Mianning, S. F. Zhu 20327 (PE); Muli, K. M. Feng 2923 (PE), T. T. Yu 14100 (PE); Shimian, C. J. Xie 42771 (PE), 42413 (PE); Tianquan, X. L. Jiang 35226 (PE); Wenchuan, Anonymous 8267 (PE); Xiangcheng, Veg. Exped. 3233 (PE); Xiaojin, P. X. Li 10030 (PE), J. Zhou E572 (PE), S. S. Chang & Y. X. Ren 6481 (PE); Yajiang, D. E. Boufford et al. 35796 (PE), West China Acad. Sci. 4116 (PE), S. X. Jia 360 (PE), H. Smith 12593 (PE), Anonymous 102001 (PE), Y. W. Tsui 5965 (PE), X. Li 76708 (PE), 76954 (PE), 76874 (PE), 76932 (PE), S. F. Zhu 20327 (PE), Anonymous 5675 (PE), Sichuan Veg. Exped. 2397 (PE); • **Xizang**: Zayü, C. W. Wang 66522 (PE), Jin W. Zhang 982 (PE), Chagyab, Qinghai-Tibet Exped. 12287 (PE); Markam, Qinghai-Tibet Exped. 11980 (PE 01824813, PE 01824814); Mainling, Xizang Med. Herb Exped. 4226 (PE); • **Yunnan**: Lijiang, G. Forrest 2508 (E 0038562, P 00711686); Shangri-la, Zhongdian Exped. 2091 (PE 00274836, PE 00274837).

#### ﻿1.4. Homostylium albescens var. salignum

Taxon classificationPlantaeAsteralesAsteraceae

(Franch.) Z.X.Fu comb. nov.

89694488-69DA-59B0-B8FD-EE10DB92D301

urn:lsid:ipni.org:names:77363586-1

≡ Inulacuspidatavar.saligna Franch., Nouv. Arch. Mus. Hist. Nat., sér. 2 10: 37. 1888. ≡ Asteralbescensvar.salignus (Franch.) Hand.-Mazz., Acta Horti Gothob. 12: 207. 1938. Type: India, George Forrest 2508 (lectotype, designated here, E 00385621!) (Suppl. material 1: fig. S7).
= Sinosidusalbescensvar.salignus (Franch.) G.L.Nesom, Phytoneuron 2020-64: 13. 2020. Type: Xizang. J.P.A. David s.n.

##### Type.

India, George Forrest 2508 (lectotype, designated here, E 00385621!) (Suppl. material 1: fig. S7).

##### Diagnosis.

***Leaves*** elliptic-lanceolate, abaxially brown pubescent on veins or sometimes totally, glandular, base attenuate, apex acuminate. ***Phyllaries*** outer narrowly lanceolate, puberulent. The main differences from the type variety include leaves long-elliptic-lanceolate, base attenuate, apex acuminate, abaxially brown tomentose along veins or entirely, stem often more robust.

##### Distribution.

China (W and N Sichuan, Xizang, Yunnan); N India. 1900 to 3900 m.

##### Habitat.

Subalpine.

##### Etymology.

The varietal name “*salignum*” is derived from the Latin “*salix*”, meaning “willow” and the neuter suffix “-*gnum*”, meaning “pertaining” or “resembling”. The name likely refers to the resemblance of this variety to willow leaves, particularly in shape.

##### Specimens examined.

**China** • **Sichuan**: Baoxing, T. P. Soong 38993 (PE), 39214 (PE); Daofu, S. Jiang et al. 2351 (PE); Jinchuan, X. Li 76838 (PE), 78065 (PE), 78362 (PE), 78490 (PE), 78611 (PE); Kangding, S. Jiang et al. 9763 (PE); Meigu, Sichuan, Econ. Pl. Exped. 1677 (PE); Muli, T. T. Yu 6605 (PE 00274903, PE 00274904), 14100 (PE); Xiaojin, G. Z. Liu 408 (PE), S. S. Chang & Y. X. Ren 5875 (PE), 6715 (PE), 6798 (PE), 6994 (PE); Yajiang, Z. Y. Luo et al. 527 (PE), Precise locality unknown, C. W. Wang 65280 (PE), 66060 (PE), K. L. Chu 7595 (PE 00274885, PE 00274886), Min. Forest. s.n. (PE); • **Xizang**: Precise locality unknown, J.P.A. David s.n.; • **Yunnan**: Binchuan, T. N. Liou 22098 (PE), 22103 (PE); Deqin, T. T. Yu 9438 (PE 00274956, PE 00274957), 9858 (PE 00274926, PE 00274927), 10535 (PE 00274906, PE 00274907), K. M. Feng 5535 (PE), 5644 (PE 00274917, PE 00274918), C. W. Wang 64762 (PE), 69994 (PE), H. T. Tsai 54400 (PE), 54416 (PE); Gongshan, C. W. Wang 67082 (PE), K. M. Feng 8391 (PE); Heqing, R. C. Ching 23614 (PE), 23937 (PE 00274910, PE 00274933); Lanping, H. T. Tsai 56284 (PE); Lijiang, T. T. Yu 15239 (PE), R. C. Ching 30994 (PE 00274912, PE 00274913), K. M. Feng 21327 (PE); Shangri-la, T. T. Yu 11550 (PE 00274919, PE 00274920), 12390 (PE 00274958, PE 00274959), 12522 (PE 00274914, PE 00274915), K. M. Feng 2749 (PE), Zhongdian Exped. 1508 (PE 00274937, PE 00274938); Precise locality unknown, T. T. Yu 15239 (PE 00274908, PE 00274909), 19693 (PE), 20823 (PE), 20925 (PE 00274953, PE 00274954), 22617 (PE), K. M. Feng 1210 (PE), H. T. Tsai 52981 (PE), Zhongdian Exped. 1413 (PE 00274939, PE 00274940); **India** • **Unknown**: Precise locality unknown, George Forrest 2508 (E 00385621).

##### Notes.

In Nesom (2020g), the type specimen for this variety was designated as “Xizang. J.P.A. David s.n.”, with neither a collection number nor a specimen number specified. According to the protologue, the holotype specimen number for Homostyliumalbescensvar.salignum was also not seen. A careful search was conducted at the PE Herbarium. More than 190 specimens of this variety were found, but still no holotype was designated. Among them, the specimen E00385621 (George Forrest 2508) (Suppl. material 1: fig. S7) closely matched the illustration in the original publication. Therefore, this specimen was designated as the lectotype of this variety.

#### ﻿1.5. Homostylium albescens var. glandulosum

Taxon classificationPlantaeAsteralesAsteraceae

(Hand.-Mazz.) Z.X.Fu comb. nov.

A010F55D-FBB3-5490-A154-7E064C3E5303

urn:lsid:ipni.org:names:77363587-1

≡ Asteralbescensvar.glandulosus Hand.-Mazz., J. Bot. 76: 284. 1938. ≡ Sinosidusalbescensvar.glandulosus (Hand.-Mazz.) G.L.Nesom, Phytoneuron 2020-64: 13. 2020. Type: China, Xizang, Chamdo, alt. 13000ft, 6 August 1933, F. Kingdon-Ward 10752 (holotype, E 00385688!; isotype, BM 000945772!) (Suppl. material 1: figs S8, S9).

##### Type.

China • Xizang, Chamdo, alt. 13000ft, 6 August 1933, F. Kingdon-Ward 10752 (holotype, E 00385688!; isotype, BM 000945772!) (Suppl. material 1: figs S8, S9).

##### Diagnosis.

***Leaves*** ovate or ovate to oblong-lanceolate, 4–10 × 1–2.5 cm, abaxially brown hairy on veins, densely glandular. It is clearly similar to H.albescensvar.salignum but has smaller leaves, more glandular points, and different hairiness.

##### Distribution.

China, S and E Xizang (Yadong, Bomi, Ranwu, Zayü, etc.), W and SW Sichuan, NW Yunnan (Weixi, Deqin, etc.); India. 1900 to 3900 m.

##### Habitat.

Subalpine.

##### Etymology.

The varietal name “*glandulosum*” is derived from the Latin “*glandula*”, meaning “gland”, and the neuter suffix “-*osum*”, meaning “full of” or “abundant in”. The name likely refers to the abundant glands on this variety, suggesting glandular leaves on the abaxial surface.

##### Specimens examined.

**China** • **Sichuan**: Dege, Y. W. Tsui 5082 (PE); Precise locality unknown, W. G. Hu & C. Ho 10929 (PE); • **Xizang**: Bomê, T. S. Ying & D. Y. Hong 650124 (PE 01825438, PE 00274703), 650193 (PE 00274701, PE 00274702), 651069 (PE), S. Z. Cheng & B. S. Li 173 (PE), 486 (PE), 617 (PE 01825439, PE 01825440); Chagyab, Qinghai-Tibet Exped. 12287 (PE), Jin W. Zhang 1098 (PE), 6316 (PE); Zayü, Z. C. Ni et al. 1091 (PE 00274679, PE 00274680); Cona, C. Y. Wu et al. 75-1099 (PE); Qamdo, F. Kingdon-Ward 10752 (E 00385688, BM 000945772), G. C. Xia et al. 1326 (PE), 1390 (PE); Gongjue, Qinghai-Tibet Exped. 12553 (PE 00274744, PE 00274745), J. S. Yang 91-493 (PE); Gyaca, Xizang Med. Herb Exped. 4551 (PE); Nyingchi, B. S. Li et al. 6410 (PE), Z. C. Ni et al. 0034 (PE 00274675, PE 00274676); Lhünzê, G. X. Fu & Jin W. Zhang 01159 (PE); Mainling, B. S. Li & S. Z. Cheng 05549 (PE), Z. C. Ni et al. 3052 (PE); Mêdog, B. S. Li & S. Z. Cheng 01135 (PE 01824803, PE 01824804); Yadong, G. X. Fu & Jin W. Zhang 1080 (PE), G. X. Fu & Jin W. Zhang 1122 (PE), G. X. Fu 907 (PE), Precise locality unknown, C. W. Wang 66550 (PE), S. X. Jia 315 (PE), 418 (PE), 682 (PE); • **Yunnan**: Deqing, T. T. Yu 8459 (PE 00274697, PE 00274698); Weixi, C. W. Wang 68048 (PE 00274699, PE 00274700).

#### ﻿1.6. Homostylium albescens var. harrowianum

Taxon classificationPlantaeAsteralesAsteraceae

(Diels) Z.X.Fu comb. nov.

67643666-9FCE-5626-8342-0C618367C7FB

urn:lsid:ipni.org:names:77363588-1

= Asterharrowianus Diels, Not. Roy. Bot. Gard. Edinb. 15: 183. 1912. Type: China, Yunnan, Dali, Moist, rocky situations, side valleys on the eastern flank of the Tali Range, lat. 25'40°N, alt. 10000–11000 ft, Sept. 1906, G. Forrest 4004 (holotype, E 00385622!) (Suppl. material 1: fig. S10).
= Asterharrowianusvar.glabratus Diels, Notes Roy. Bot. Gard. Edinburgh 5: 184. 1912. Type: China. Yunnan, Lijiang, shady, rocky situations, side valleys on the eastern flank, Lat. 27'15°N, alt. 9500–11000 ft, July 1906, G. Forrest 2508 (holotype, E 00385621!; isotype, P 00711686!) (Suppl. material 1: figs S5, S6).
≡ Asterlimprichtiivar.gracilior Hand.-Mazz., Symb. Sin. 7: 1093. 1936. ≡ Asteralbescensvar.gracilior (Hand.-Mazz.) Hand.-Mazz., Acta Horti Gothob. 12: 206. 1938. ≡ Sinosidusalbescensvar.gracilior (Hand.-Mazz.) G.L.Nesom, Phytoneuron 2020-64: 13. 2020. Type: China, Sichuan, Muli, alt. 2800 m, 31 July 1915, Hand.-Mazz. 7350 (holotype, WU 0061122!; isotype, A 00003821!) (Suppl. material 1: figs S11, S12).

##### Type.

China • Sichuan, Muli, alt. 2800 m, 31 July 1915, Hand.-Mazz. 7350 (holotype, WU 0061122!; isotype, A 00003821!) (Suppl. material 1: figs S11, S12).

##### Diagnosis.

***Leaves*** elliptic-lanceolate, 7–20 × 1–5 cm, abaxially densely white or gray-white tomentose or cottony, base attenuate, apex acuminate. ***Phyllaries*** outer oblong or lanceolate. The tomentose of this variety is the same as that of Homostyliumalbescensvar.limprichtii, but its leaf shape is similar to that of H.albescensvar.salignum.

##### Distribution.

China, W Sichuan (Jinchuan, Heishui, etc.), Chongqing (Wushan, Nanchuan, etc.), Guizhou, NW Yunnan (Dali), Gansu, and S Shaanxi.

##### Habitat.

Temperate biome and subalpine.

##### Etymology.

The varietal name “*harrowianum*” is derived from the name of Harrow, with the Latin neuter adjectival suffix “-*ianum*”, indicating possession or association. The variety was likely named in honor of Harrow, either the collector name or the place of collection in London, Britain.

##### Specimens examined.

**China** • **Chongqing**: Precise locality unknown, T. N. Liou 9842 (PE 00274690, PE 00274691); • **Gansu**: Wenxian, Z. Y. Zhang 14794 (PE); • **Guizhou**: Chishui, Bijie Exped. 1243 (PE 00274634, PE 00274635); Zunyi, Sichuan-Guizhou Exped. 1542 (PE 00274637, PE 00274638, PE 00274640); • **Shaanxi**: Mianxian, S. Jiang et al. 232 (PE), T. N. Liou & P. C. Tsoong 3411 (PE), • **Sichuan**: Xiangcheng, S. Jiang et al. 9611 (PE); Kangding, K. C. Kuan et al. 77 (PE 00274687, PE 00274688, PE 00274689); Lixian, C. L. Wu 33209 (PE), S. Jiang et al. 1958 (PE); Muli, Hand.-Mazz. 7350 (WU 0061122, A 00003821); • **Yunnan**: Dali, T. N. Liou 17471 (PE 00274716, PE 00274728), 17631 (PE), 20950 (PE), 21031 (PE), R. C. Ching 22719 (PE), H. C. Wang 770 (PE 00274659, PE 00274660), 865 (PE), 990 (PE), 1140 (PE 00274723, PE 00274725), 1144 (PE), 1450 (PE), 1458 (PE 00274717, PE 00274730), 1574 (PE 00274721, PE 00274727), 4302 (PE), 4404 (PE), H. T. Tsai 53877 (PE), G. Forrest 4004 (E 00385622); Lijiang, G. Forrest 2508 (E 00385621, P 00711686).

##### Notes.

The autonyms take priority over other variety names established simultaneously, as well as over other synonyms, according to Articles 11.6 of ICN (Turland et al. 2018). Asterharrowianusvar.harrowianus (1912), established alongside A.harrowianusvar.glabratus (1912), takes precedence over A.limprichtiivar.gracilior (1936) and A.albescensvar.gracilior (1938). Accordingly, for this new combination, we adopted the varietal epithet “*harrowianus*” and adjusted it to the neuter form “*harrowianum*” to match the gender of *Homostylium*.

#### ﻿1.7. Homostylium albescens var. limprichtii

Taxon classificationPlantaeAsteralesAsteraceae

(Diels) Z.X.Fu comb. nov.

A6BD7EB3-FD86-56F8-8FEF-A84998CD9D2C

urn:lsid:ipni.org:names:77363589-1

≡ Asterlimprichtii Diels, Repert. Spec. Nov. Regni Veg. Beih. 12: 503. 1922. ≡ Asteralbescensvar.limprichtii (Diels) Hand.-Mazz., Acta Horti Gothob. 12: 206. 1938. ≡ Sinosidusalbescensvar.limprichtii (Diels) G.L.Nesom, Phytoneuron 2020-64: 13. 2020. Type: China, Sichuan, Batang-Litang, alt. 3400 m, 21 August 1914, H. W. Limpricht 2226 (lectotype, designated here, WRSL S07-7760!; isolectotypes, A 00003820!, PE no. 32347!, WU 0061126!) (Suppl. material 1: figs S13–S16).

##### Type.

China • Sichuan, Batang-Litang, alt. 3400 m, 21 August 1914, H. W. Limpricht 2226 (lectotype, designated here, WRSL S07-7760!; isolectotypes, A 00003820!, PE no. 32347!, WU 0061126!) (Suppl. material 1: figs S13–S16).

##### Diagnosis.

***Leaves*** elliptic or oblong, 3–7 × 1–3 cm, abaxially densely white or gray-white tomentose or cottony, base broadly cuneate or rounded. ***Phyllaries*** outer ovate, sparsely tomentose. The main differences from the type variety include leaves elliptic or oblong, base broadly cuneate or rounded, apex obtuse, abaxially densely tomentose or cottony, white to gray-white.

##### Distribution.

China, W and NW Sichuan (Songpan, Maoxian, Lixian, Kangding, Heishui, Hanyuan), Xizang, and W Gansu (Xigu, Longnan). 2400–3100 m.

##### Habitat.

Temperate biome and subalpine.

##### Etymology.

The varietal name “*limprichtii*” is derived from the name of German botanist and teacher Hans Wolfgang Limpricht, with the Latin eponymic suffix “-*ii*”. The variety was likely named in honor of Mr. Limpricht, the collector of the type specimens.

##### Specimens examined.

**China** • **Gansu**: Longnan, T. P. Wang 14702 (PE 00274842, PE 00274845), 15235 (PE), 15212 (PE); • **Sichuan**: Heishui, X. Li & J. X. Zhou 73734 (PE); Kangding, H. Smith 13414 (PE); Maoxian, C. Ho & Z. L. Zhou 12984 (PE); Songpan, T. P. Wang 7760 (PE); Hanyuan, T. P. Wang 8706 (PE); Jiuzhaigou, P. Q. Li 149 (PE), H. Smith 10355 (PE); Batang-Litang, H. W. Lim­pricht 2226 (WRSL S07-7760, A 00003820, PE no. 32347, WU 0061126); • **Xizang**: Zayü, Qinghai-Tibet Exped. 161 (PE 00274856, PE 00274857), Z. C. Ni et al. 195 (PE 00274677, PE 00274678).

##### Notes.

Nesom (2020g) only provided the type specimen information with collection locality, collector, and collection number (Xizang, Limpricht 2226) for this variety, without specifying an exact specimen number. According to the protologue, the holotype specimen number for Homostyliumalbescensvar.limprichtii was not seen. A careful search was conducted at the PE Herbarium. Forty-three specimens of this variety were found, but still no holotype was designated. Among them, the specimen WRSL S07-7760 (H. W. Limpricht 2226) (Suppl. material 1: figs S13–S16) closely matched the illustration in the original publication. Therefore, this specimen was designated as the lectotype of this variety. The duplicate specimens (syntypes) from the same collections were then treated as isolectotypes.

#### ﻿1.8. Homostylium albescens var. megaphyllum

Taxon classificationPlantaeAsteralesAsteraceae

(Y.Ling) Z.X.Fu comb. nov.

0E4BE71C-B030-5C81-A59E-F014A1B52A18

urn:lsid:ipni.org:names:77363590-1

≡ Asteralbescensvar.megaphyllus Y.Ling, Fl. Reipubl. Popularis Sin. 74: 358, 185. 1985. ≡ Sinosidusalbescensvar.megaphyllus (Y.Ling) G.L.Nesom, Phytoneuron 2020-64: 13. 2020. Type: China, Sichuan, Lixian, C. Ho & Z. L. Zhou 13318 (holotype, PE 00274858!) (Suppl. material 1: fig. S17).

##### Type.

China • Sichuan, Lixian, C. Ho & Z. L. Zhou 13318 (holotype, PE 00274858!) (Suppl. material 1: fig. S17).

##### Diagnosis.

***Stems*** yellow-brown pilose and white arachnoid. ***Leaves*** elliptic or ovate-lanceolate, 10–15 × 5–7 cm, adaxially arachnoid pilose, reticulate veins prominent, abaxially sparsely pilose, midvein white villous, base rounded or broadly cuneate, margin subentire, apex acute or subrounded. ***Phyllaries*** outer ovate, sparsely puberulent. The leaf shape of this variety differs from all other varieties.

##### Distribution.

China, W Sichuan (Lixian, Maoxian, Wenchuan).

##### Habitat.

Subalpine.

##### Etymology.

The varietal name “*megaphyllum*” is derived from the Greek “*mega*-”, meaning “large” or “grate”, and the “-*phyllon*”, meaning “leaf”, combined with the Latin neuter suffix “-*um*”. The name likely refers to this variety having larger leaves than other varieties.

##### Specimens examined.

**China** • **Sichuan**: Lixian, C. Ho & Z. L. Zhou 13318 (PE).

#### ﻿1.9. Homostylium albescens var. pilosum

Taxon classificationPlantaeAsteralesAsteraceae

(Hand.-Mazz.) Z.X.Fu comb. nov.

F3EB54FA-F040-5059-935D-A7241D90C911

urn:lsid:ipni.org:names:7363591-1

≡ Asteralbescensvar.pilosus Hand.-Mazz., Acta Horti Gothob. 12: 207. 1938. ≡ Sinosidusalbescensvar.pilosus (Hand.-Mazz.) G.L.Nesom, Phytoneuron 2020-64: 13. 2020. Type: China, Yunnan, Lijiang, Ninglang and Yongsheng, 15 May 1922. J. F. Rock 5164 (holotype, WU, not seen; isotypes, E 00385623!, WU 1937-0004375!) (Suppl. material 1: figs S18, S19).

##### Type.

China • Yunnan, Lijiang, Ninglang and Yongsheng, 15 May 1922, J. F. Rock 5164 (holotype, WU, not seen; isotypes, E 00385623!, WU 1937-0004375!) (Suppl. material 1: figs S18, S19).

##### Diagnosis.

***Leaves*** oblong-lanceolate, 4–9 × 1–2 cm, abaxially on midvein or totally white pilose, adaxially sparsely hispidulous, flat. ***Phyllaries*** outer sparsely hairy. achenes densely pilose. The main differences from type variety include leaves oblong-lanceolate, abaxially sparsely pilose along the midvein or entirely, adaxially sparsely hispid, achenes densely villous.

##### Distribution.

Common. SW China (W Sichuan, E Xizang, NW Yunnan). 2800–4000 m.

##### Habitat.

Subalpine.

##### Etymology.

The varietal name “*pilosum*” is derived from the Latin “*pilus*”, meaning “hair”, combined with the neuter suffix “-*osum*”, meaning “full of” or “abundant in”. The name likely refers to abundant pilose on the abaxial surface of leaves.

##### Specimens examined.

**China** • **Sichuan**: Dege, Anonymous 115 (PE); Daocheng, Bot. Exped. 2436 (PE); Kangding, H. Smith 10436 (PE), 12912 (PE), 13990 (PE); Jinchuan, X. Li 78185 (PE); Lixian, S. Jiang et al. 1608 (PE); Precise locality unknown, S. Jiang et al. 2018 (PE); • **Xizang**: Nyingchi, Xizang Med. Herb Exped. 3450 (PE), D. E. Boufford et al. 30173 (PE), Y. T. Zhang & K. Y. Lang 1315 (PE 00274876, PE 00274877), B. S. Li et al. 6247 (PE); Mainling, Qinghai-Tibet Exped. 1961 (PE 00274878, PE 00274879), Xizang Med. Herb Exped. 3724 (PE), Qinghai-Tibet Supplement Team 750817 (PE 00274874, PE 00274875); Precise locality unknown, B. S. Li & S. Z. Cheng 5376 (PE 01824815, PE 01824816); • **Yunnan**: Dali, R. C. Ching 22919 (PE), Qin et al. 649 (PE); Qiaojia, B. X. Sun et al. 908 (PE); Lijiang, J. F. Rock 5164 (E 00385623, WU 1937-0004375).

#### ﻿1.10. Homostylium albescens var. rugosum

Taxon classificationPlantaeAsteralesAsteraceae

(Y.Ling) Z.X.Fu comb. nov.

AD2A6413-5AF4-5B97-B9AC-1870415955C6

urn:lsid:ipni.org:names:77363592-1

≡ Asteralbescensvar.rugosus Y.Ling, Fl. Reipubl. Popularis Sin. 74: 358, 184. 1985. Type: China, Yunnan, Qinghua university 5895 (holotype, PE 02050377!) (Suppl. material 1: fig. S20).
= Sinosidusalbescensvar.rugosus (Y.Ling) G.L.Nesom, Phytoneuron 2020-64: 13. 2020. Type: China, Sichuan, Kangding, 1893, Soulie 900 (paratypes, PE 02050375!, PE 02050376!) (Suppl. material 1: figs S21, S22).

##### Type.

China • Yunnan, Qinghua university 5895 (holotype, PE 02050377!) (Suppl. material 1: fig. S20).

##### Diagnosis.

***Leaves*** oblong-lanceolate, adaxially hispidulous, reticulate veins prominent, areoles of reticulate veins with foamy process. Leaf shape is similar to that of the previous varieties, but with more prominently raised reticulate venation, making the interveinal areas bullate, adaxially sparsely hispidulous.

##### Distribution.

China, NW Yunnan (Dali) and W Sichuan (Huili).

##### Habitat.

Subalpine.

##### Etymology.

The varietal name “*rugosum*” is derived from the Latin “*ruga*”, meaning “wrinkle” or “fold”, combined with the neuter suffix “-*osum*”, meaning “full of” or “abundant in”. The name likely refers to the uneven leaf surface, indicating the presence of a foamy process on the adaxial surface.

##### Specimens examined.

**China** • **Sichuan**: Huili, T. T. Yu 1605 (PE); Kangding, Soulie 900 (PE 02050375, PE 02050376); • **Yunnan**: Precise locality unknown, Qinghua university 5895 (PE 02050377).

##### Notes.

The polymorphism of *H.albescens* is evident. In addition to the varieties listed herein, there is also a variety of Homostyliumalbescensvar.niveum distributed in Sikkim. According to Grierson (1964), its abaxial leaf surface is covered with cottony tomentum. The plants observed in western China with a cottony tomentose abaxial leaf surface should likely be merged into either H.albescensvar.limprichtii or H.albescensvar.harrowianum.

#### ﻿1.11. Homostylium albescens var. niveum

Taxon classificationPlantaeAsteralesAsteraceae

(Hand.-Mazz.) Z.X.Fu comb. nov.

B209DEA8-CBA3-5A04-B82C-DC84DCB99299

urn:lsid:ipni.org:names:77363594-1

≡ Asteralbescensvar.niveus Hand.-Mazz., Acta Hort. Gothob. 12: 208. 1938. Type: India, Sikkim, Jakpho, Napa Hill, alt. 3017 m, 25 Oct. 1885, C. B. Clarke 41356 (holotype, E 00531283!; isotype, K 00089039!) (Suppl. material 1: figs S23, A24).

##### Type.

India • Sikkim, Jakpho, Napa Hill, alt. 3017 m, 25 Oct. 1885, C. B. Clarke 41356 (holotype, E 00531283!; isotype, K 00089039!) (Suppl. material 1: figs S23, A24).

##### Diagnosis.

***Leaves*** abaxially densely cottony tomentum.

##### Distribution.

China, Chongqing, W Sichuan (Emei Mount., Xiaojin), SE Shaanxi (Pingli); N India (Sikkim).

##### Habitat.

Subalpine or subarctic biome.

##### Etymology.

The varietal name “*niveum*” is derived from the Latin “*nix*”, meaning “snow”, combined with the neuter suffix “-*eum*”. The name likely refers to its habitat in the snow-covered subalpine and subarctic biome, or to the abaxial leaf densely covered in white tomentum.

##### Specimens examined.

**China** • **Chongqing**: Nanchuan, J. H. Xiong & Z. L. Zhou 94046; • **Shaanxi**: Pingli, Field Collection Team of Western Xida’an Kang District 20132; • **Sichuan**: Emei, W. P. Fang 3297, 3182; Xiaojin, T. T. Yu 2429; **India** • **Sikkim**: Jakpho, C. B. Clarke 41356 (E 00531283, K 00089039).

##### Notes.

The new combination *Sinosidusalbescens* (DC.) G.L.Nesom proposed by Nesom (2020g) did not include this variety, although it comprised ten varieties (including the type variety).

#### ﻿2. Homostylium argyropholium

Taxon classificationPlantaeAsteralesAsteraceae

(Hand.-Mazz.) Z.X.Fu comb. nov.

14C83666-672C-55E6-B0FE-E29CAF1143DA

urn:lsid:ipni.org:names:77363595-1

Figs 4 , 5

≡ Asterargyropholis Hand.-Mazz., Acta Horti Gothob. 12: 208. 1938. ≡ Sinosidusargyropholis (Hand.-Mazz.) G.L.Nesom, Phytoneuron 2020-64: 13. 2020. Type: China, Sichuan, Kangding (Tatien-lu), frutex fere metralis in declivo aprico, 2 Jul 1922, H. Smith 2258 (holotype, UPS, v-080214!) (Suppl. material 1: fig. S25).

##### Type.

China • Sichuan, Kangding (Tatien-lu), frutex fere metralis in declivo aprico, 2 Jul 1922, H. Smith 2258 (holotype, UPS, v-080214!) (Suppl. material 1: fig. S25).

##### Description.

Shrubs, 93–228 cm tall. ***Leaves*** subleathery, alternate, elliptic, oblong, or lanceolate-ovate, 1–4 × 0.3–1.6 cm; midvein abaxially prominent, lateral veins apparent, pinnate; margin subundulate to entire, revolute, glandular-punctate, upper leaves smaller; adaxially green, moderately scabridulous, minutely glandular, resin-dotted, scabrous; abaxially densely lanate or greyish-white arachnoid-tomentose. ***Capitula*** 4–10(-20), with 15–20 ray florets, in corymbiform synflorescences, terminal on axillary branches. ***Peduncles*** slender, 2–7 mm, bracteoles linear. ***Involucres*** campanulate, ca. 5 mm long, 8 mm in diameter. ***Phyllaries*** 4–5 seriate, imbricate, pubescent to glabrescent, outer phyllaries oblong, 1 × 0.5 mm, inner phyllaries ovate, 4–5 × 1 mm.

**Figure 4.**
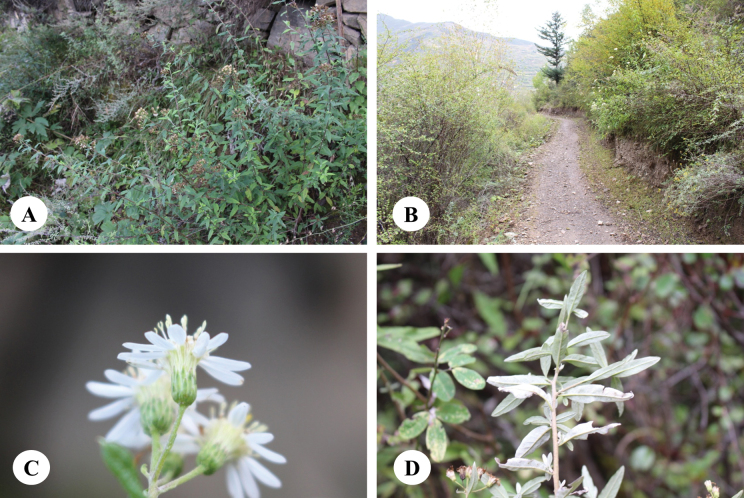
*Homostyliumargyropholium* (Hand.-Mazz.) Z.X.Fu. **A.** Habit; **B.** Habitat; **C.** Capitula (lateral view, showing the involucre); **D.** Abaxial surface of leaves. Photographed by Z. X. Fu from Z. X. Fu 2944 (PE).

##### Distribution.

**Figure 5.**
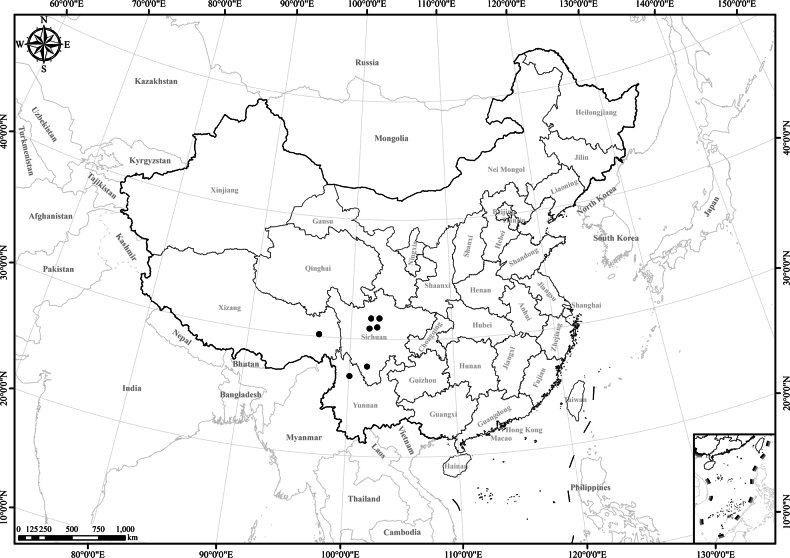
Distribution of *Homostyliumargyropholium*. China is centrally located on the map.

China, S and W Sichuan, SE Xizang, NW Yunnan (Fig. 5).

##### Habitat.

Alpine and subalpine slopes, under forests, grasslands, riverbanks. 2100–3100 m.

##### Phenology.

Flowering May to October. Fruiting August to October.

##### Etymology.

It is derived from the Greek “*argyros*”, meaning “silver”, and “*pholis*”, meaning “skin” or “scale”, combined with the Latin neuter suffix “-*um*”. The specific epithet likely refers to the silvery, scale-like, glandular punctate on the leaves.

##### Notes.

This species is similar to Homostyliumalbescensvar.discolor. However, it can be distinguished by its fewer capitula borne at the branch tips, narrower involucre, and white buds in the leaf axils. *H.polium* bears soft and white arachnoid-tomentose on the abaxial leaves, particularly resembling *H.argyropholium*. However, it has much smaller leaves, and inflorescences borne on lateral branches, allowing for easy differentiation from *H.argyropholium*.

#### ﻿2.1. Homostylium argyropholium var. argyropholium

Taxon classificationPlantaeAsteralesAsteraceae

7B119ECE-D666-56B3-B415-4BAC43E32AA5

##### Diagnosis.

***Leaves*** abaxially densely tomentose or pubescence, secondary veins abaxially not prominent, apex acute or abruptly acute, rarely rounded. ***Peduncles*** densely tomentulose. ***Involucres*** 4–5 mm. ***Phyllaries*** 4-seriate. The leaves exhibit considerable variation, with apices either obtuse or acuminate.

##### Distribution.

Common. China, Sichuan (Damba, Jinchuan, Lixian, Barkham, Xiaojin), and Xizang (Basu).

##### Habitat.

Subalpine.

##### Specimens examined.

**China** • **Sichuan**: Kangding (Tatien-lu), H. Smith 2258 (UPS v-080214); Danba, Z. X. Fu 2970 (PE), 2971 (PE), 2972 (PE); Jinchuan, Z. X. Fu 2993 (PE), 2994 (PE), 2995 (PE), 2996 (PE), 2997 (PE), 2998 (PE), 2999 (PE), 3001 (PE), X. Li 76624 (PE), 77595 (PE 00275057, PE 00274598), 75154 (PE), 77904 (PE), 75124 (PE), D. Y. Hong et al. 95047 (PE); Lixian, C. S. Cao 193 (PE), 1569 (PE), G. J. Zhang 145 (PE), 146 (PE), Z. X. Fu 3054 (PE), 3055 (PE), 3056 (PE), 3058 (PE); Barkam, T. Y. Chang & H. F. Zhou 23111 (IBSC, KUN), Z. X. Fu 3003 (PE), 3010 (PE), 3011 (PE), 3012 (PE), 3013 (PE), 3015(PE), 3016 (PE), 3017 (PE), S. Jiang & C. L. Jin 1026 (PE 00275056, PE 01776701, PE 01822231), T. Y. Chang & H. F. Zhou 22631 (PE, KUN, IBSC), 23024 (PE, KUN, IBSC), Z. B. Feng 960306 (HX), G. J. Zhang 148 (PE), 151 (PE), 152 (PE), 153 (PE), T. Y. Chang & H. F. Zhou 23467 (PE, KUN); Xiaojin, Z. X. Fu 2944 (PE), J. Zhou 86 (PE, IBSC), H. N. Qin, S. X. Yu & J. Y. Wu 17087 (PE); • **Xizang**: Baxoi, J. S. Yang 90-251 (PE).

#### ﻿2.2. Homostylium argyropholium var. niveum

Taxon classificationPlantaeAsteralesAsteraceae

(Y.Ling) Z.X.Fu comb. nov.

8CD50D5B-63D7-5C11-A1D8-BF2E08529FFF

urn:lsid:ipni.org:names:77363596-1

≡ Asterargyropholisvar.niveus Y.Ling, Fl. Reipubl. Popularis Sin. 74: 358. 1985. ≡ Sinosidusargyropholis (Hand.-Mazz.) G.L.Nesom, Phytoneuron 2020-64: 13. 2020. Type: China, Sichuan, Lixian, alt. 2700 m, 8 July 1930, F. T. Wang 21626 (holotype, PE 00275069!) (Suppl. material 1: fig. S26).

##### Type.

China • Sichuan, Lixian, alt. 2700 m, 8 July 1930, F. T. Wang 21626 (holotype, PE 00275069!) (Suppl. material 1: fig. S26).

##### Diagnosis.

***Leaves*** abaxially densely white tomentum, secondary veins abaxially not prominent, apex obtuse or rounded. ***Peduncles*** densely white tomentum. ***Involucres*** 4–5 mm. ***Phyllaries*** 4-seriate. The leaves’ coloration could distinctly differentiate it from the other varieties.

##### Distribution.

China, W Sichuan (Jinchuan, Luding, Lixian, Barkam, Maoxian, Yanyuan), and NW Yunnan (Lijiang).

##### Habitat.

Subalpine.

##### Etymology.

The varietal name “*niveum*” is derived from the Latin “*nix*”, meaning “snow”, combined with the neuter suffix “-*eum*”. The name likely refers to its abaxial leaf and inflorescence peduncles densely covered in white tomentum.

##### Specimens examined.

**China** • **Sichuan**: Jinchuan, X. Li 77625 (PE); Luding, Anonymous 6828 (PE 01824233, CDBI 0141863, CDBI 0141864, CDBI 0141865); Barkam, P. X. Li 10061 (PE); Maoxian & Lixian, C. Ho & Z. L. Zhou 13058 (PE); Lixian, F. T. Wang 21626 (PE 00275069); • **Yunnan**: Lijiang, W. L. He & C. Y. Zhao 20344 (PE, KUN), R. C. Ching 20344 (KUN).

#### ﻿2.3. Homostylium argyropholium var. paradoxum

Taxon classificationPlantaeAsteralesAsteraceae

(Y.Ling) Z.X.Fu comb. nov.

EE14E639-9043-5290-A97F-920B98854539

urn:lsid:ipni.org:names:77363597-1

≡ Asterargyropholisvar.paradoxus Y.Ling, Fl. Reipubl. Popularis Sin. 74: 358. 1985. ≡ Sinosidusparadoxus (Y.Ling) G.L.Nesom, Phytoneuron 2020-64: 13. 2020. Type: China, Sichuan, Barkam, alt. 2700 m, 18 July 1957, X. Li 23091 (holotype, PE 00275072!; isotype, KUN, not seen) (Suppl. material 1: fig. S27).

##### Type.

China • Sichuan, Barkam, alt. 2700 m, 18 July 1957, X. Li 23091 (holotype, PE 00275072!; isotype, KUN, not seen) (Suppl. material 1: fig. S27).

##### Diagnosis.

**Leaves** abaxially sparsely tomentose, secondary veins abaxially prominent. **Involucres** 5–6 mm. **Phyllaries** 5-seriate. The lateral veins of abaxial leaves in this variety are prominently elevated and covered with fine tomentum, differentiating it from the type variety.

##### Distribution.

China, W Sichuan (Barkam, Jinchuan). Jinchuan is a new distribution record at the county level.

##### Habitat.

Subalpine.

##### Etymology.

The varietal name “*paradoxum*” is derived from the Greek “*paradoxos*”, meaning “contrary to expectation” or “unexpected”, combined with the Latin neuter suffix “-*um*”. The name likely refers to a distinctive or unexpected characteristic that distinguishes this variety from others.

##### Specimens examined.

**China** • **Sichuan**: Barkam, T. Y. Chang & H. F. Zhou 23091 (PE, IBSC, KUN, QTPMB), Z. X. Fu 3004 (PE), 3005 (PE), 3006 (PE), 3007 (PE), 3008 (PE), 3014 (PE), 3018 (PE); Jinchuan, Z. X. Fu 3002 (PE).

#### ﻿3. Homostylium fulgidulum

Taxon classificationPlantaeAsteralesAsteraceae

(Griers.) Z.X.Fu comb. nov.

05EFC189-8DE3-54B3-866C-34C163F4E040

urn:lsid:ipni.org:names:77363598-1

Figs 6 , 7 , 8

≡ Asterfulgidulus Griers., Notes Roy. Bot. Gard. Edinburgh 26: 110. 1964. ≡ Sinosidusfulgidulus (Grierson) G.L.Nesom, Phytoneuron 2020-64: 13. 2020. Type: China, Xizang, Bomê, Tangme (Tongmai), Tsangpo-Yigrong Confluence, alt. 7000 ft, 3 June 1947, F. Ludlow, G. Sherriff & H. H. Elliott 13074 (holotype, E 00385686!; isotype, BM 000945773!) (Suppl. material 1: figs S28, S29).

##### Type.

China • Xizang, Bomê, Tangme (Tongmai), Tsangpo-Yigrong Confluence, alt. 7000 ft, 3 June 1947, F. Ludlow, G. Sherriff & H. H. Elliott 13074 (holotype, E 00385686!; isotype, BM 000945773!) (Suppl. material 1: figs S28, S29).

##### Description.

Shrubs, 126–192 cm tall. ***Leaves*** alternate, ovate, (4-)6–9 × (2-)2.4–4.8 cm, venation prominently pinnate, intercostal glossy; margin entire, revolute, abaxially sparsely strigillose, glossy between minor veins, sparsely minutely glandular; adaxially glabrous or glabrate, sparsely minutely glandular, midvein sparsely strigillose. ***Capitula*** numerous, in terminal corymbiform synflorescences. ***Peduncles*** 1.5–2 cm long. ***Involucres*** campanulate, 5–7 mm in diameter. ***Phyllaries*** 3–4 seriate, imbricate, 1.5–3 × 1 mm, outer series ovate, shorter, inner series ovate, 6–7 × 0.5 mm.

**Figure 6.**
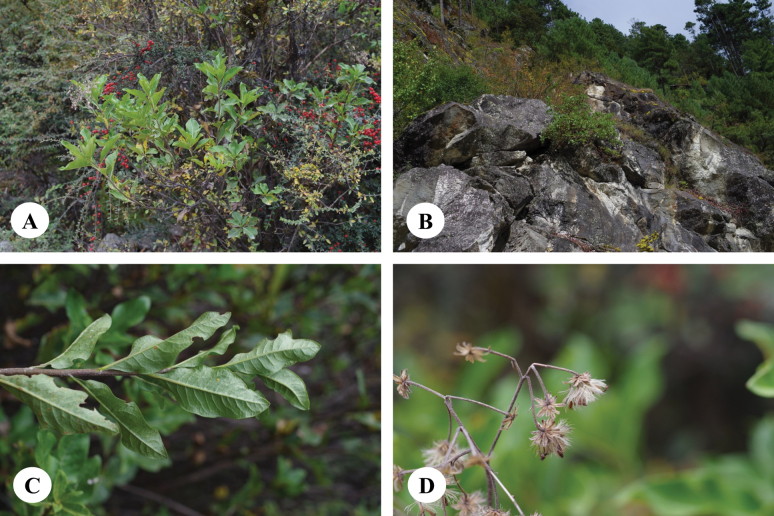
*Homostyliumfulgidulum* (Grierson) Z.X.Fu. **A.** Habit; **B.** Habitat; **C.** Abaxial surface of leaves; **D.** Capitula. Photographed by G. J. Zhang from B. H. Jiao & G. J. Zhang 294 (PE).

##### Distribution.

**Figure 7.**
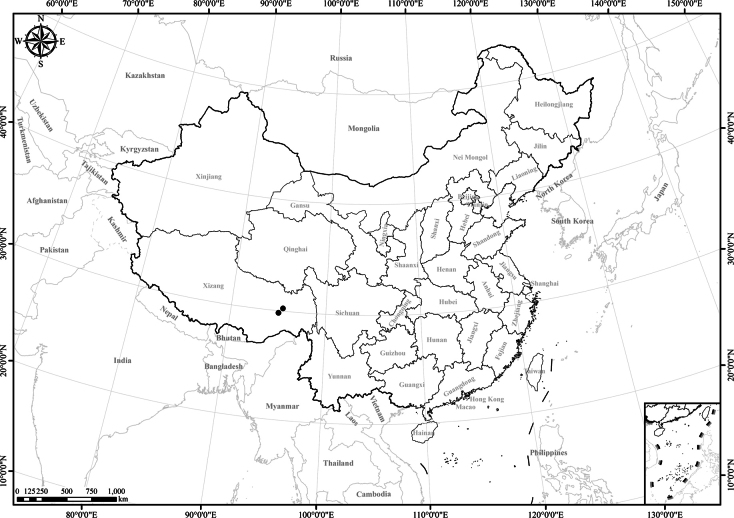
Distribution of *Homostyliumfulgidulum*. China is centrally located on the map.

China, SE Xizang (Bomê and Nyingchi). 2200–3000 m (Fig. 7).

##### Habitat.

Margins of submontane forest and hillside shrublands.

##### Phenology.

Flowering June to August. Fruiting June to August.

##### Etymology.

The name “*fulgidulum*” is derived from the Latin “*fulgidus*”, meaning “shining” or “radiant”, combined with the Latin neuter suffix “-*ulum*”, meaning “diminutive”. The specific epithet likely refers to its abaxially glossy intercostal veins on the surface of leaves.

##### Specimens examined.

**China** • **Xizang**: Bomê, T. S. Ying & D. Y. Hong 650705 (PE), Jin W. Zhang & J. T. Wang 440 (PE), G. J. Zhang & B. H. Jiao 294 (PE), W. L. Zheng 293 (XZ), D. E. Boufford et al. 29838 (PE), F. Ludlow, G. Sherriff & H. H. Elliott 13074 (E 00385686, BM 000945773); Nyingchi, B. S. Li et al. 6410 (PE), Xizang Med. Herb Exped. 3542 (PE), T. Naito et al. 1099 (PE), G. Yao et al. 1282 (XZ), W. L. Zheng 985 (XZ), W. L. Zheng et al. 3236 (XZ), W. L. Zheng et al. 3239 (XZ), Anonymous 22 (XZ).

**Figure 8.**
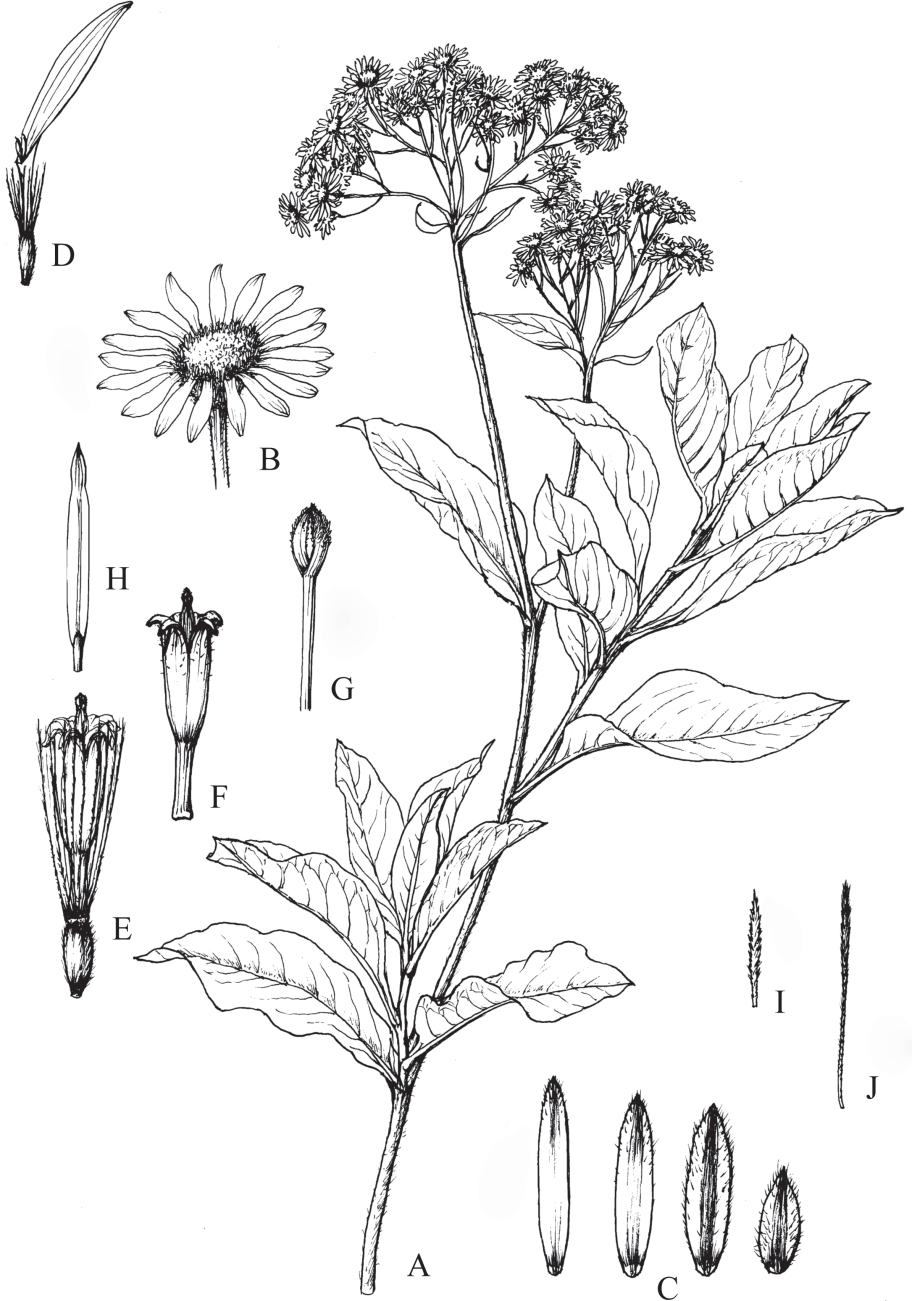
*Homostyliumfulgidulum* (Grierson) Z.X.Fu. **A.** Habit; **B.** Capitulum; **C.** Involucral bracts; **D.** Ray floret; **E.** Disc floret; **F.** Floret with pappus bristle and removed achene; **G.** Style branches of disc floret; **H.** Stamen of disc floret; **I.** Outer pappus bristle; **J.** Inner pappus bristle. Drawn by Z. J. Chen.

##### Notes.

This species closely resembles *Homostyliumalbescens*. However, it could be differentiated from the latter by its ovate, larger, both surfaces nearly glabrous leaves, and abaxially glossy intercostal veins.

#### ﻿4. Homostylium hypoleucum

Taxon classificationPlantaeAsteralesAsteraceae

(Hand.-Mazz.) Z.X.Fu comb. nov.

070C9BF1-806A-5C9F-AFF2-97C5A7574B34

urn:lsid:ipni.org:names:77363599-1

Figs 9 , 10 , 11

≡ Asterhypoleucus Hand.-Mazz., J. Bot. 76: 285. 1938. ≡ Sinosidushypoleucus (Hand.-Mazz.) G.L.Nesom, Phytoneuron 2020-64: 13. 2020. Type: China, Xizang, Nangxian, Kyimdong Dzong, F. Kingdon-Ward 11993 (holotype, BM 000945771!) (Suppl. material 1: fig. S30).

##### Type.

China • Xizang, Nangxian, Kyimdong Dzong, F. Kingdon-Ward 11993 (holotype, BM 000945771!) (Suppl. material 1: fig. S30).

##### Description.

Dwarf shrubs, cespitose, 12–36 cm tall. ***Leaves*** alternate, elliptic to oblanceolate, 0.3–1.7 × 0.19–0.33 cm, leathery, margins entire or (1-)2-serrate-spinose, strongly revolute, adaxially dark green, glabrate, arachnoid, or sparsely sericeous, abaxially densely white tomentose. ***Capitula*** 1–3, terminal on lateral branches. ***Peduncles*** 1.5–3 cm long, sparsely strigose, with few subulate bracteoles. ***Involucres*** subcampanulate, 4–5 mm in diameter. ***Phyllaries*** 4-seriate, imbricate, outer phyllaries extremely short, ovate-lanceolate, inner phyllaries linear-lanceolate, 5 × 0.6 mm, 4–5 × as long as outer phyllaries.

**Figure 9.**
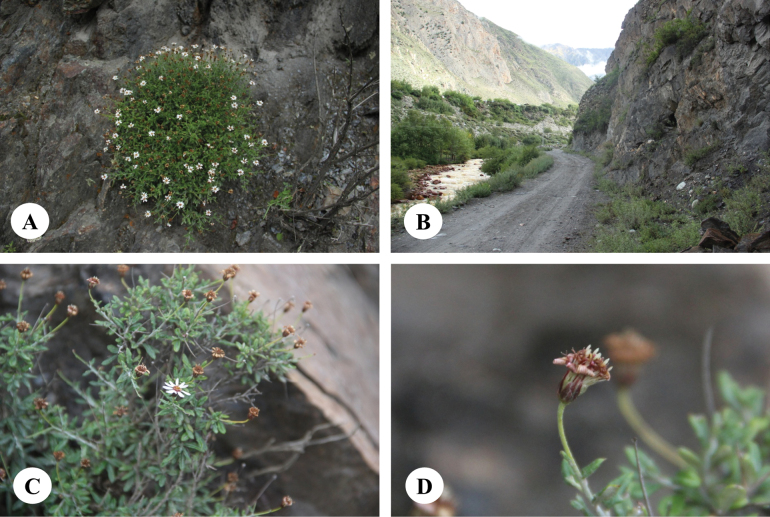
*Homostyliumhypoleucum* (Hand.-Mazz.) Z.X.Fu. **A.** Habit; **B.** Habitat; **C.** Upper portion of plant; **D.** Capitulum. Photographed by Z. X. Fu from Z. X. Fu 1475 (PE).

##### Distribution.

**Figure 10.**
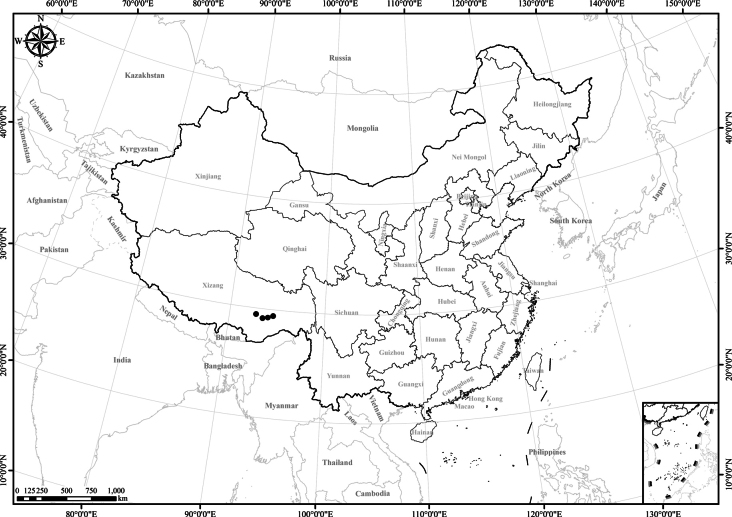
Distribution of *Homostyliumhypoleucum*. China is centrally located on the map.

Endemic to China, SE Xizang (Lang, Gyaca, Mainling, and Zangbo Valley) (Fig. 10).

##### Habitat.

Arid mountain slopes of river valleys, rock crevices, woodland margins, and shrublands. 3000–3700 m.

**Figure 11.**
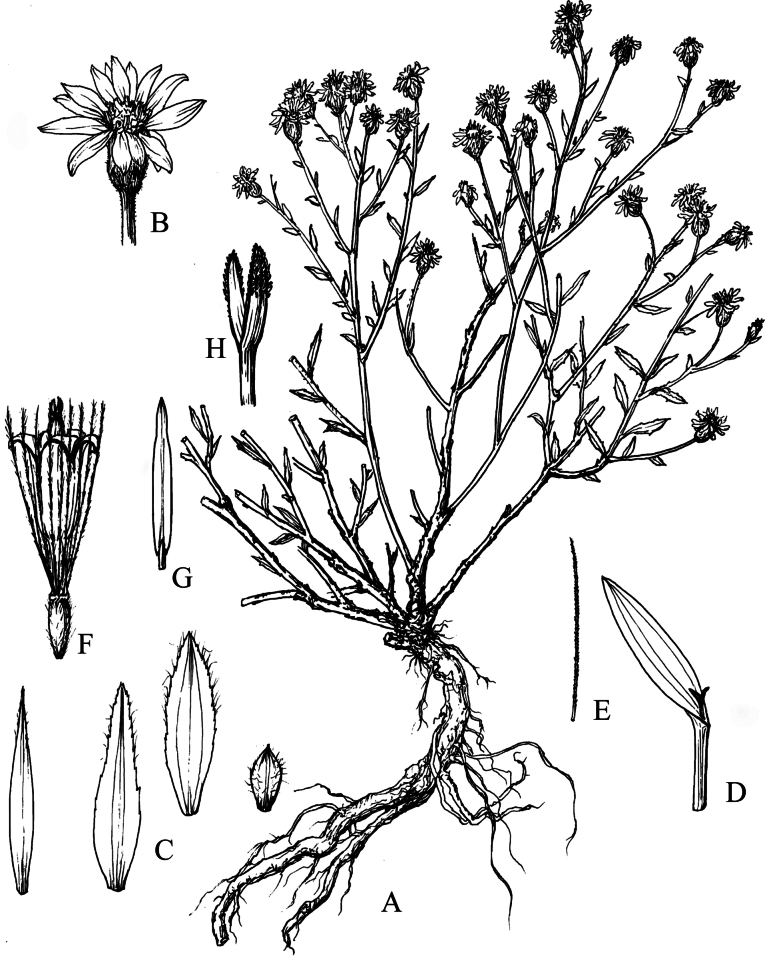
*Homostyliumhypoleucum* (Hand.-Mazz.) Z.X.Fu. **A.** Habit; **B.** Capitulum; **C.** Involucral bracts; **D.** Ray floret; **E.** Pappus bristle; **F.** Disc floret; **G.** Stamen of disc floret; **H.** Style branches of disc floret. Drawn by Z. J. Chen.

##### Phenology.

Flowering July to August. Fruiting August to September.

##### Etymology.

The specific epithet “*hypoleucum*” is derived from the Greek “*hypo*-”, meaning “under” or “beneath”, and “*leucos*”, meaning “white”, combined with the Latin neuter suffix “-*um*”. The name likely refers to the whitish coloration on the abaxial surface of the leaves (abaxially densely covered with thick white tomentum) in this species.

##### Specimens examined.

**China** • **Xizang**: Gyaca, B. Q. Xu et al. XiaNh–07zx–0639 (IBSC), Z. C. Ni et al. 2724 (PE 01836991, PE 01836993, PE 01836992), Qinghai–Xizang Supplement Team 750652 (PE 00275826, PE 00275825), Xizang Med. Herb Exped. 4399 (PE 01532326, PE 00275824); Nangxian, Z. X. Fu 1475 (PE), 1476 (PE), 1477 (PE), 1478 (PE), 1479 (PE), Qinghai–Xizang Exped. 7899 (PE 01532325, PE 01532063), Qinghai–Xizang Veget. Exped. 3314 (PE 01836952, PE 01532324), H. N. Qin et al. 554 (PE), J. Luo et al. L048 (QTPMB), Xizang Med. Herb Exped. 4250 (PE 00275828, PE 00275828), Z. C. Ni et al. 2757 (PE), F. Kingdon-Ward 11993 (BM 000945771); Mainling, Xizang Med. Herb Exped. 4302 (PE 01532328, PE 00275827).

##### Notes.

This species can be easily distinguished from other shrubby *Homostylium* by its dwarf shrub habit and the persistent peduncles. Its leaf shape is similar to that of *H.lavandulifolium*. However, *H.hypoleucum* has relatively shorter and smaller leaves without adaxial papillate protrusions, capitula not arranged in a dense corymbose, broader involucres, and white pappus, which are diagnostic characters.

#### ﻿5. Homostylium lavandulifolium

Taxon classificationPlantaeAsteralesAsteraceae

(Hand.-Mazz.) Z.X.Fu comb. nov.

A4EF9E76-05F0-511B-99A9-F59C3EDED6F5

urn:lsid:ipni.org:names:77363600-1

Figs 12 , 13 , 14

≡ Asterlavandulifolius Hand.-Mazz., Notizbl. Bot. Gart. Berlin-Dahlem 13: 609. 1937. ≡ Sinosiduslavandulifolius (Hand.-Mazz.) G.L.Nesom, Phytoneuron 2020-64: 14. 2020. Type: China, Sichuan, Muli, watershed of the Shou-chu river and Shou-chu valley, in dry gorge, alt. 2435–2900 m, June 1928, J.C.F. Rock 16273 (lectotype, designated by Nesom (2020g), W, not seen; isolectotypes, US 00145655!, A 00003817!, GH 00003818!, E 00413420!) (Suppl. material 1: figs S31–S34).

##### Type.

China • Sichuan, Muli, watershed of the Shou-chu river and Shou-chu valley, in dry gorge, alt. 2435–2900 m, June 1928, J. F. Rock 16273 (lectotype, designated by Nesom (2020g), W, not seen; isolectotypes, US 00145655!, A 00003817!, GH 00003818!, E 00413420!) (Suppl. material 1: figs S31–S34).

##### Description.

Shrubs, 48–124 cm tall. ***Leaves*** subleathery, narrowly linear, 1–4(-5.4) × 0.1–0.3(-0.53) cm, margins entire, revolute; midvein prominently glabrous, venation pinnate; adaxially green, rugose, resinous, very sparsely sericeous to nearly glabrous, papillate protrusions; abaxially densely gray-white tomentose; upper leaves smaller. ***Capitula*** 6–9 mm in diameter, 3–6 at ends of lateral branches or numerous in ± densely corymbiform synflorescences, terminal on current-year branches. ***Peduncles*** 2–6 mm long, with subulate bracteoles. ***Involucres*** obconical to campanulate, 5–7 × 4–7 mm. ***Phyllaries*** 4–5-seriate, imbricate, outer phyllaries ovate, ca. 1 mm × 0.5 mm, inner phyllaries longer, linear-lanceolate, sparsely tomentose, 4–5 × 0.7–0.9 mm.

**Figure 12.**
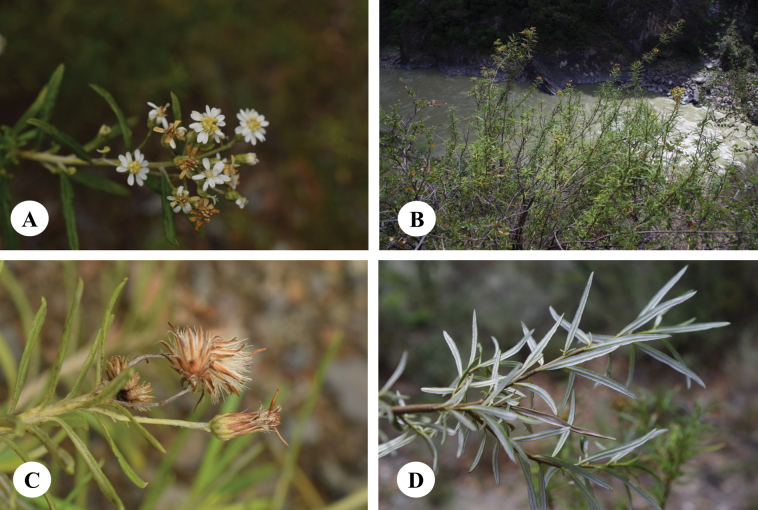
*Homostyliumlavandulifolium* (Hand.-Mazz.) Z.X.Fu. **A.** Habit; **B.** Habitat; **C.** Capitula; **D.** Abaxial surface of leaves. **A, C.** From FLPH Sichuan Exped. 151845 (PE), photographed by R. Ke, in Muli, Sichuan. **B, D.** From G. J. Zhang 170 (PE), photographed by G. J. Zhang.

##### Distribution.

**Figure 13.**
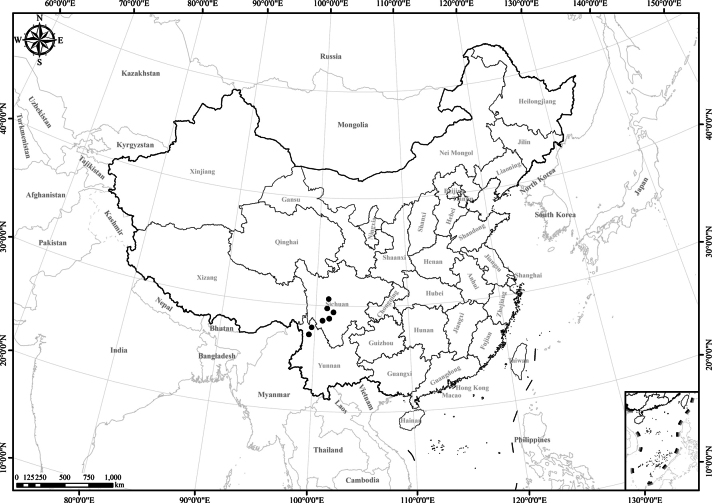
Distribution of *Homostyliumlavandulifolium*. China is centrally located on the map.

Endemic to China, W Sichuan (Daocheng, Jiulong, Kangding, Muli, Yajiang), and NW Yunnan (Fig. 13).

##### Habitat.

Subalpine stony slopes, riverbanks, and shrublands of aridly hot river valleys. 2000–2900 m.

##### Phenology.

Flowering June to August. Fruiting August to September.

##### Specimens examined.

**China** • **Sichuan**: Daocheng, Sichuan Veg. Exped. 2109 (CDBI); Jiulong, S. Jiang et al. 3918 (PE), T. T. Yu 6694 (PE), X. J. Yang 3935 (PE); Kangding, Z. J. Zhao et al. 114731 (CDBI), Y. S. Chen 7566 (PE); Muli, T. T. Yu 5894 (PE, IBSC), Y. S. Chen 7099 (PE), Y. S. Chen 7507 (PE), J.C.F. Rock 16273 (W, US 00145655, A 00003817, GH 00003818, E 00413420), FLPH Sichuan Exped. 151845 (PE); Yajiang, G. J. Zhang 170 (PE), Z. T. Guan56-0438 (PE), Z. P. Huang et al. 552 (PE), S. Jiang et al. 3202 (PE), Wei L. Chen et al. 6604 (PE).

**Figure 14.**
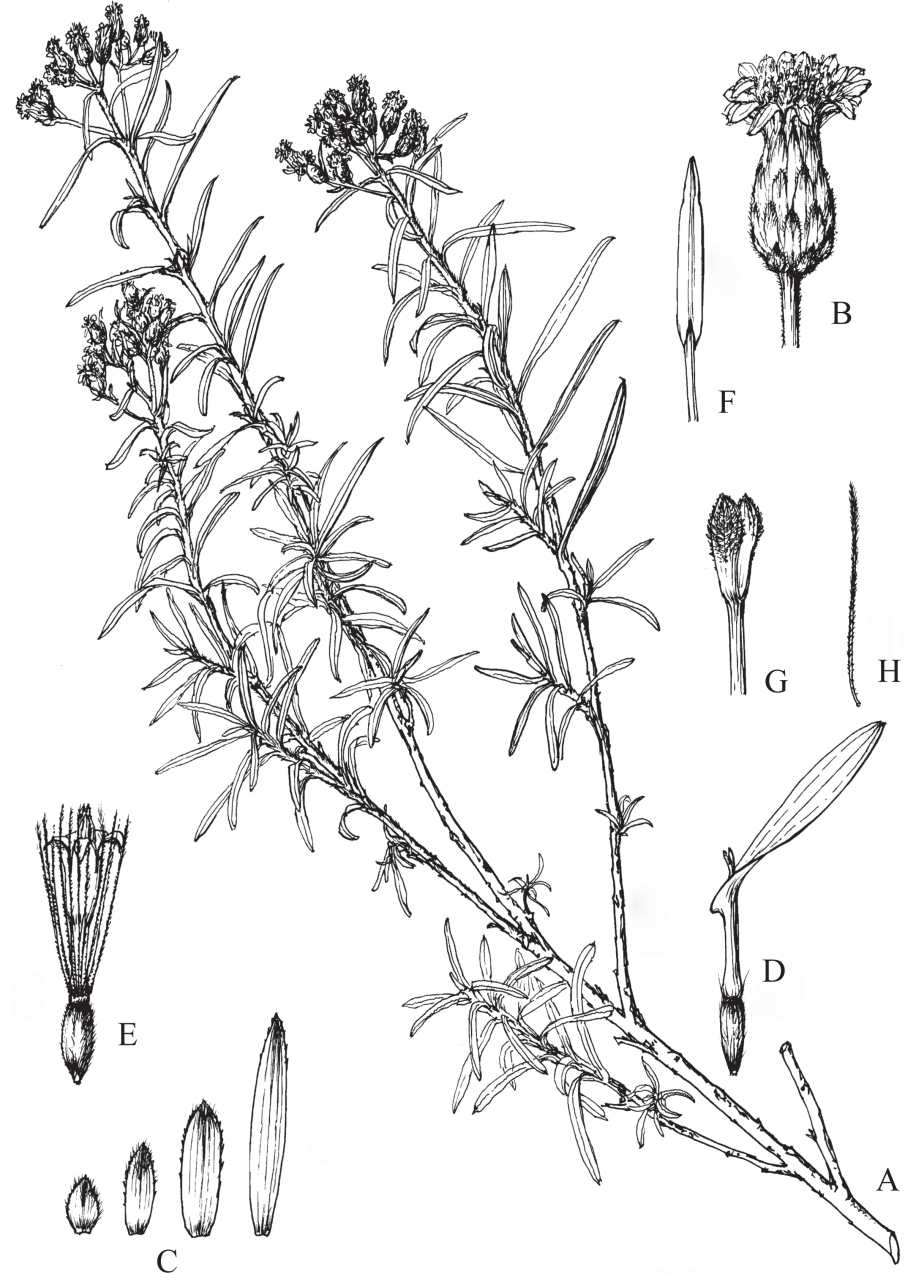
*Homostyliumlavandulifolium* (Hand.-Mazz.) Z.X.Fu. **A.** Habit; **B.** Capitulum; **C.** Involucral bracts; **D.** Ray floret; **E.** Disc floret; **F.** Stamen of disc floret; **G.** Style branches of disc floret; **H.** Pappus bristle. Drawn by Z. J. Chen.

##### Etymology.

The name “*lavandulifolium*” is derived from the Latin “*lavandula*”, referring to the genus *Lavandula* L., combined with the neuter suffix “-*folium*”, meaning “leaf” or “have the form of”. The specific epithet likely refers to the linear leaf shape of this species, resembling the distinctive form of *Lavandula*.

##### Notes.

This species is particularly distinctive within *Homostylium*. The leaves of this species are narrowly linear, with revolute margins. The abaxial surface is pubescent, with prominent midrib, and cylindric achenes.

#### ﻿6. Homostylium motuoense

Taxon classificationPlantaeAsteralesAsteraceae

(Y.L.Chen) Z.X.Fu comb. nov.

2BD6EC6E-C218-5245-9182-924B997554CA

urn:lsid:ipni.org:names:77363601-1

Figs 15 , 16

≡ Astermotuoensis Y.L.Chen, Bull. Bot. Res. 8: 12. 1988. ≡ Sinosidusmotuoensis (Y.L.Chen) G.L.Nesom, Phytoneuron 2020-64: 14. 2020. Type: China, Xizang, Mêdog, Qarasa, gudeng-ganhua, in prato lapidoso secus marginem rivuli, alt. 1100 m, Dec. 1982, S. Z. Cheng & B. S. Li 2181 (holotype, PE 02050341!; isotypes, PE 02050372!, PE 02050373!, PE 02050374!) (Suppl. material 1: figs S35–S38).

##### Type.

China • Xizang, Mêdog, Qarasa, gudeng-ganhua, in prato lapidoso secus marginem rivuli, alt. 1100 m, Dec. 1982, S. Z. Cheng & B. S. Li 2181 (holotype, PE 02050341!; isotypes, PE 02050372!, PE 02050373!, PE 02050374!) (Suppl. material 1: figs S35–S38).

##### Description.

Shrubs, 53–166 cm tall. ***Leaves*** alternate, subcoriaceous, median leaves oblong-lanceolate to narrowly oblong, (1-)3–4(-6.3) × 0.7–1.4 cm, margins entire, revolute, upper leaves gradually smaller, adaxially dark green, prominent venation, glossy, abaxially densely grayish-white to white tomentose except on midvein. ***Capitula*** numerous, small, radiate, 2–3 mm in diameter, with 4–6 ray florets, in densely corymbiform synflorescences, terminal on branch tips or in upper leaf axils. ***Peduncles*** slender, densely white tomentum. ***Involucres*** cylindrical or subcylindrical, 5.3–6.4 × 2 mm. ***Phyllaries*** 4–5-seriate, imbricate, subcoriaceous, outer phyllaries very small, ovate, ca. 1 mm long, sparsely pubescent, inner phyllaries oblong-lanceolate, 4.2–5.2 × 0.5–1 mm.

##### Distribution.

**Figure 15.**
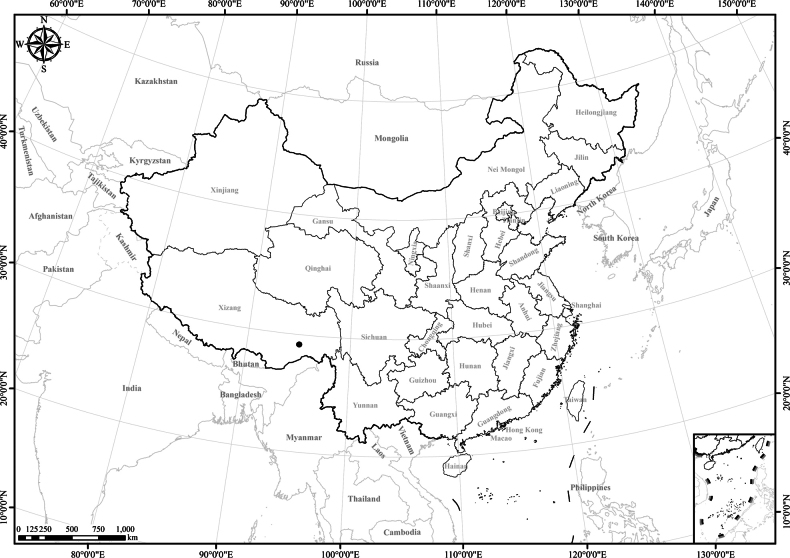
Distribution of *Homostyliummotuoense*. China is centrally located on the map.

Endemic to China, SE Xizang, Mêdog (Fig. 15).

##### Habitat.

Riverbank slopes, roadside, and dry grassy slopes near villages. 980–1100 m.

**Figure 16.**
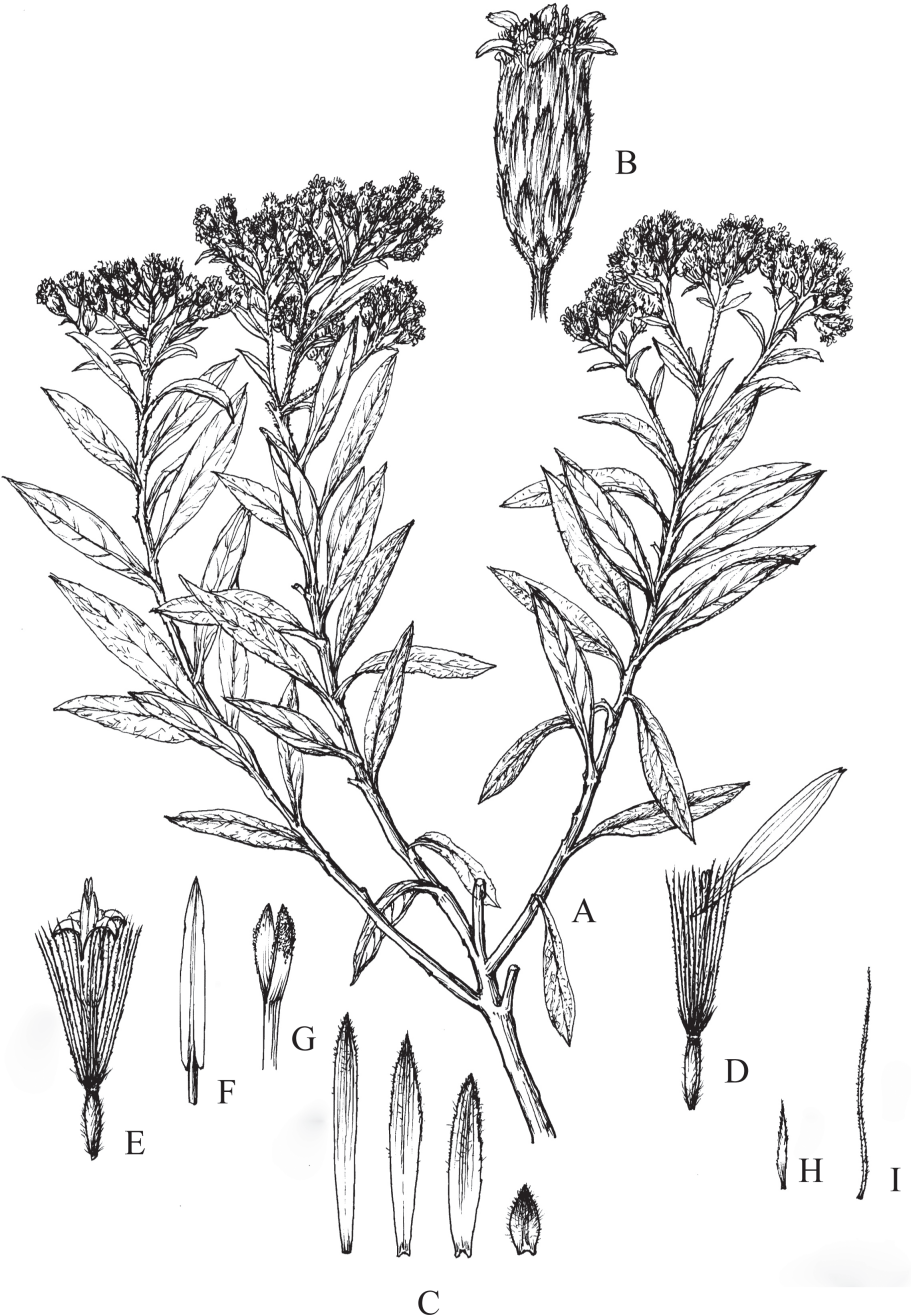
*Homostyliummotuoense* (Y.L.Chen) Z.X.Fu. **A.** Habit; **B.** Capitulum; **C.** Involucral bracts; **D.** Ray floret; **E.** Disc floret; **F.** Stamen of disc floret; **G.** Style branches of disc floret; **H.** Outer pappus bristle; **I.** Inner pappus bristle. Drawn by Z. J. Chen.

##### Phenology.

Flowering and fruiting ca. November.

##### Etymology.

The specific epithet “*motuoense*” is derived from the place name “Motuo” combined with the Latin neuter suffix “-*ense*”, meaning “from” or “of”. The name refers to the species being found and collected in Motuo (Mêdog) County, located in SE Tibet, China.

##### Specimens examined.

**China** • **Xizang**: Mêdog, S. Z. Cheng & B. S. Li 1796 (PE), 2075 (PE), 2181 (PE 02050341, PE 02050372, PE 02050373, PE 02050374).

##### Notes.

Due to transportation difficulties in Mêdog County (Xizang), we were unable to photograph, collect, or sequence *Homostyliummotuoense*. As a result, 6 *Homostylium* species, excluding *H.motuoense*, have been sequenced and used to reconstruct phylogenetic relationship within *Aster* and Astereae to date (Li et al. 2012; Zhang et al. 2015, 2019; Fu et al. 2019; Chen et al. 2024). The morphology of this species resembles *H.lavandulifolium*, but its leaves are narrowly oblong or elongated-lanceolate, with a glossy surface and prominent venation. The involucres are cylindrical. The ray florets 4–6, with two layers of pappus: the outer layer is very short, and the inner layer is 4 cm long, rough-haired. These characteristics make it easily distinguishable from *H.lavandulifolium*.

#### ﻿7. Homostylium polium

Taxon classificationPlantaeAsteralesAsteraceae

(Schneid.) Z.X.Fu comb. nov.

05EB6FCA-38F8-5B58-8DD0-D48D51AA370E

urn:lsid:ipni.org:names:77363602-1

Figs 17 , 18 , 19

≡ Asterpolius C.K.Schneid., Pl. Wilson. (Sargent) 3: 459. 1917. ≡ Sinosiduspolius (C.K.Schneid.) G.L.Nesom, Phytoneuron 2020-64: 14. 2020. Type: China, Sichuan, Xiaojin (=Nin Monkong Ting), head of Chin Ho Valley, alt. 7000–9000 ft, June 1908, E. H. Wilson 2233 (holotype, A 00003825!; isotype, US 01696632!) (Suppl. material 1: figs S39, S40).

##### Type.

China • Sichuan, Xiaojin (=Nin Monkong Ting), head of Chin Ho Valley, alt. 7000–9000 ft, June 1908, E. H. Wilson 2233 (holotype, A 00003825!; isotype, US 01696632!) (Suppl. material 1: figs S39, S40).

##### Description.

Shrubs, 56–118 cm tall. ***Leaves*** subleathery, alternate, narrowly ovate to elliptic, 1.3–3.7 × 0.4–1.6 cm, margin entire, strongly revolute, adaxially green, scabridulous, verrucose-pubescent, abaxially densely white tomentose or arachnoid, including the midrib. ***Capitula*** 1.5 cm in diameter, radiate, numerous, 3–10(-20), in corymbiform synflorescences, terminal on current-year lateral branches. ***Peduncles*** slender, 5–10 mm long. ***Involucres*** campanulate or broadly campanulate, 5–6 × 5–7 mm. ***Phyllaries*** 4–5-seriate, imbricate, outer phyllaries ovate, ca. 1.2 mm long, narrowly lanceolate, inner phyllaries unequal, lanceolate, 3–4 × ca. 1 mm.

**Figure 17.**
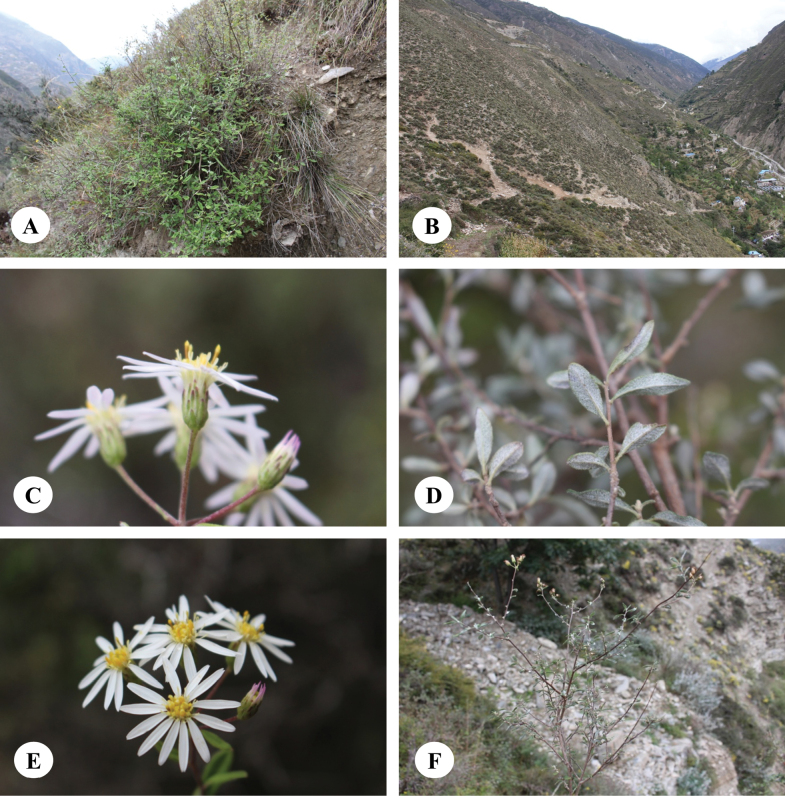
*Homostyliumpolium* (Schneid.) Z.X.Fu. **A.** Habit; **B.** Habitat; **C.** Capitula (lateral view, showing the involucre); **D.** Abaxial surface of leaves; **E.** Capitula; **F.** Branches of stem (showing capitula terminal on lateral branches). Photographed by Z. X. Fu from Z. X. Fu 2918 (PE).

##### Distribution.

**Figure 18.**
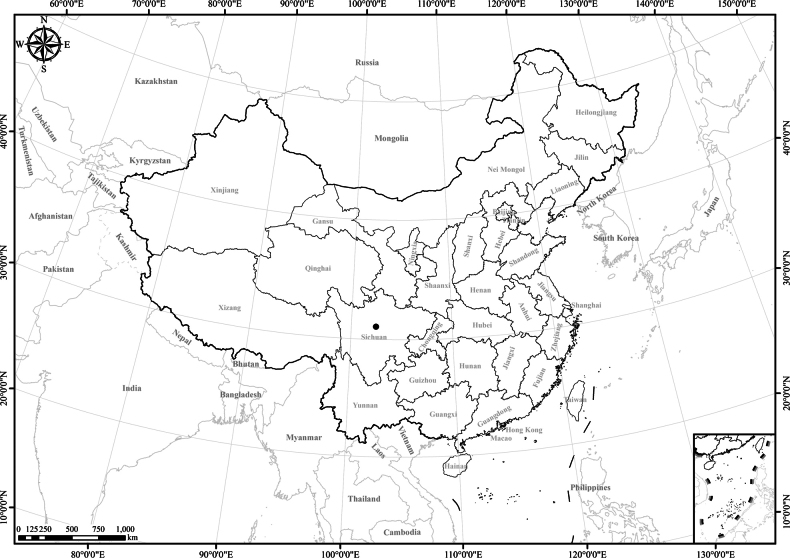
Distribution of *Homostyliumpolium*. China is centrally located on the map.

Endemic to China, Sichuan, Xiaojin (Fig. 18).

##### Habitat.

Shrubland of arid river valleys. 2000–2700 m.

**Figure 19.**
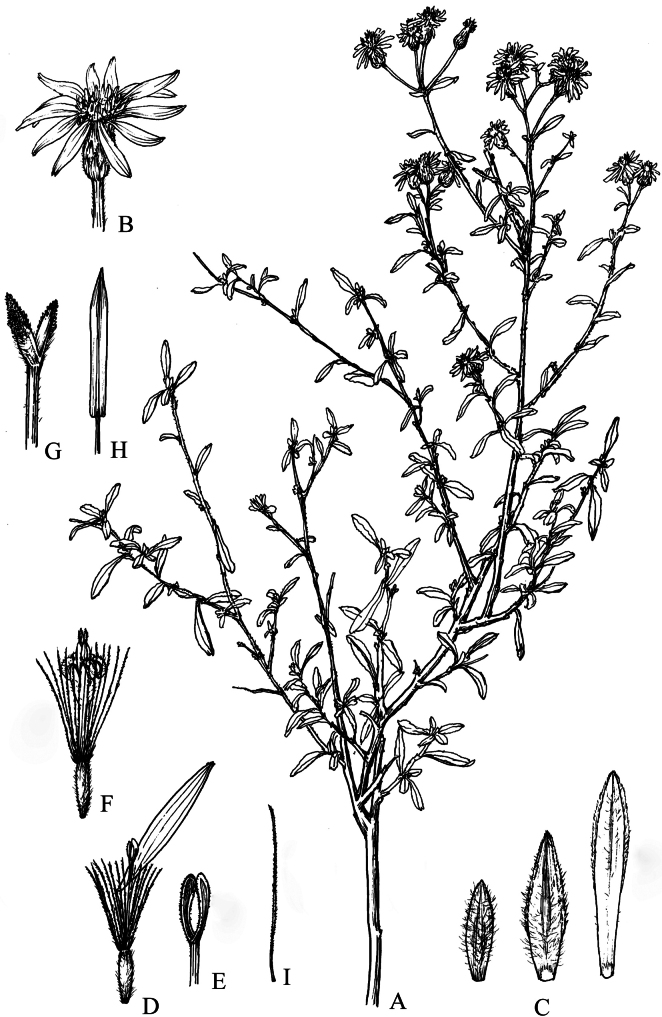
*Homostyliumpolium* (Schneid.) Z.X.Fu. **A.** Habit; **B.** Capitulum; **C.** Involucral bracts; **D.** Ray floret; **E.** Style branches of ray floret; **F.** Disc floret; **G.** Style branches of disc floret; **H.** Stamen of disc floret; **I.** Pappus bristle. Drawn by Z. J. Chen.

##### Phenology.

Flowering and Fruiting ca. July to September.

##### Etymology.

The specific epithet “*polium*” is derived from the Greek “*polios*”, meaning “gray” or “hoary”, combined with the Latin neuter suffix “-*um*”. The name likely refers to the grayish indumentum on the stems or the adaxial leaf surface.

##### Specimens examined.

**China** • **Sichuan**: Xiaojin, Z. X. Fu 2918 (PE), 2919 (PE), 2920 (PE), 2921 (PE), 2922 (PE), 2923 (PE), 2924 (PE), 2925 (PE), 2927 (PE), E. H. Wilson 2233 (A 00003825, US 01696632).

##### Notes.

Xiaojin County is located at the junction of the China-Japan Forest Subregion and the China-Himalayan Plant Subregion, resulting in a diverse vegetation composition. Compared to surrounding areas, the county faces harsh environmental conditions, characterized by water scarcity and aridity. A distinctive feature of the region is the widespread presence of mesophotic, drought-tolerant sparse shrub vegetation. It also underscores the restricted distribution of *H.polium*. Based on combined ITS and ETS data, Fu et al. (2019) reconstructed a BI phylogenetic tree for *Aster*, including five *Homostylium* species. The results showed that *H.polium* is more closely related to *H.argyropholium*, compared to *H.lavandulifolium*. These three species, together with *H.albescens* and *H.fulgidulum*, cluster into a monophyletic clade, with *H.fulgidulum* positioned as the basal species.

## Supplementary Material

XML Treatment for Homostylium

XML Treatment for Homostylium albescens

XML Treatment for Homostylium albescens var. albescens

XML Treatment for Homostylium albescens var. discolor

XML Treatment for Homostylium albescens var. glabratum

XML Treatment for Homostylium albescens var. salignum

XML Treatment for Homostylium albescens var. glandulosum

XML Treatment for Homostylium albescens var. harrowianum

XML Treatment for Homostylium albescens var. limprichtii

XML Treatment for Homostylium albescens var. megaphyllum

XML Treatment for Homostylium albescens var. pilosum

XML Treatment for Homostylium albescens var. rugosum

XML Treatment for Homostylium albescens var. niveum

XML Treatment for Homostylium argyropholium

XML Treatment for Homostylium argyropholium var. argyropholium

XML Treatment for Homostylium argyropholium var. niveum

XML Treatment for Homostylium argyropholium var. paradoxum

XML Treatment for Homostylium fulgidulum

XML Treatment for Homostylium hypoleucum

XML Treatment for Homostylium lavandulifolium

XML Treatment for Homostylium motuoense

XML Treatment for Homostylium polium

## ﻿Additional information

### Author ORCIDs

Xinyu Chen https://orcid.org/0009-0008-6347-2490

Zhixi Fu https://orcid.org/0000-0002-2789-6287

### Data availability

All of the data that support the findings of this study are available in the main text or Supplementary Information.

## Supplementary materials

Supplementary material 1 Supplementary information Data type pdf Explanation note The supplementary file provides 40 images of the type specimens examined for the taxa discussed in this study. https://binary.pensoft.net/file/1355121This dataset is made available under the Open Database License (http://opendatacommons.org/licenses/odbl/1.0/). The Open Database License (ODbL) is a license agreement intended to allow users to freely share, modify, and use this Dataset while maintaining this same freedom for others, provided that the original source and author(s) are credited.Xinyu Chen, Zhixi Fu
