# Supplementary material for: ﻿Resurrection of the genus Homostylium Nees for the former Aster ser. Albescentes Y.Ling (Astereae, Asteraceae), with an updated generic synopsis
Source: PhytoKeys. 2025 Jun 17;259:27–66. doi: 10.3897/phytokeys.259.155606 (PMC12239000; doi:10.3897/phytokeys.259.155606)
Supplement: Supplementary material 1 — Supplementary information [file phytokeys-259-027_article-155606__-s001.pdf]

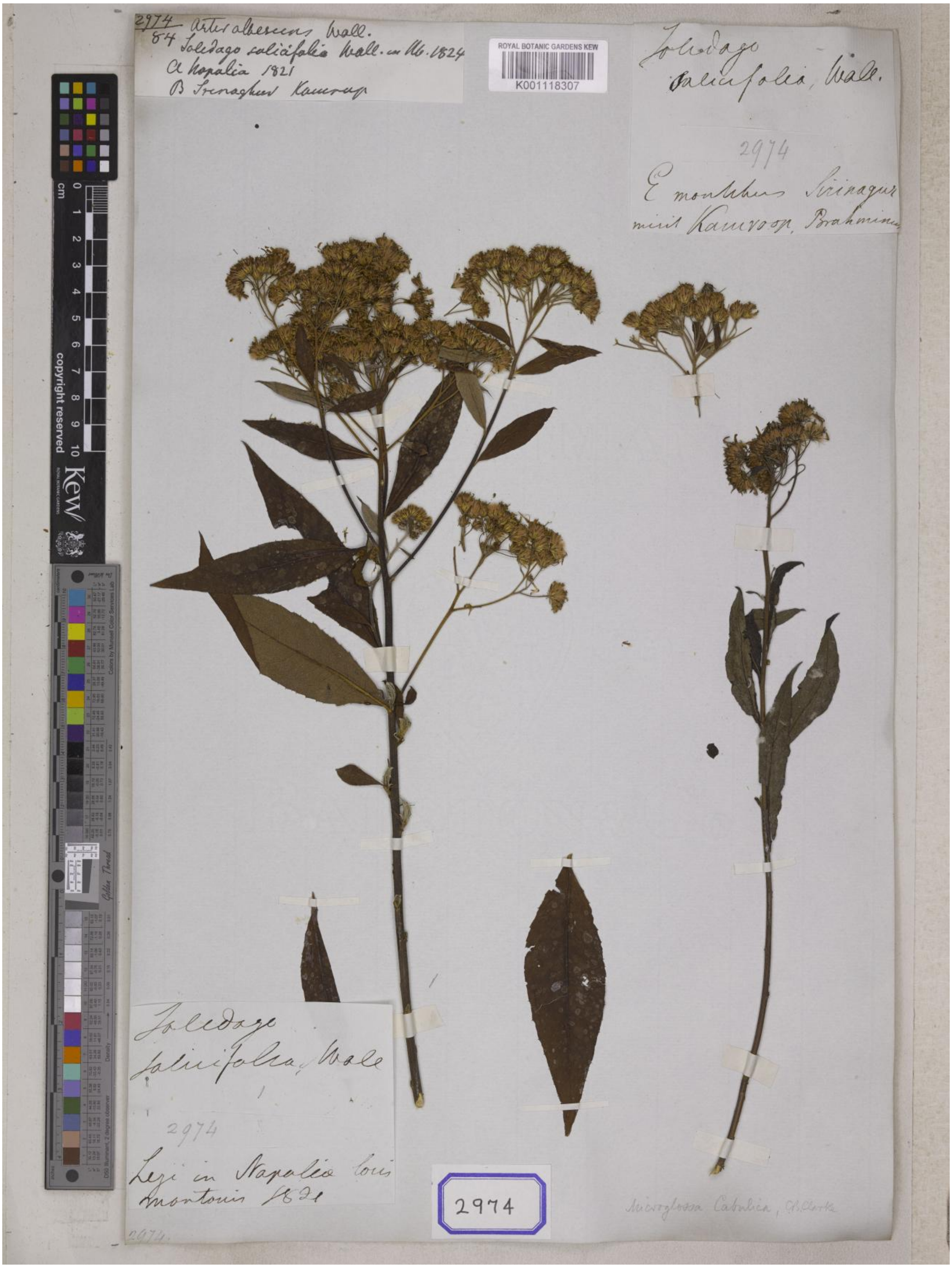

figure S1. ***Homostylium albescens*** (DC.) Z.X.Fu, **comb. nov.** ≡ *Amphirhapis albescens* DC. ≡ *Microglossa albescens* (DC.) C.B.Clarke ≡ *Aster albescens* (DC.) Wall. ex Hand.-Mazz. ≡ *Sinosidus albescens* (DC.) G.L.Nesom. Nepal, Kamaon, Gossain-Than, N. Wallich 2974/84 (lectotype, designated by Nesom (2020g), K 001118307!).

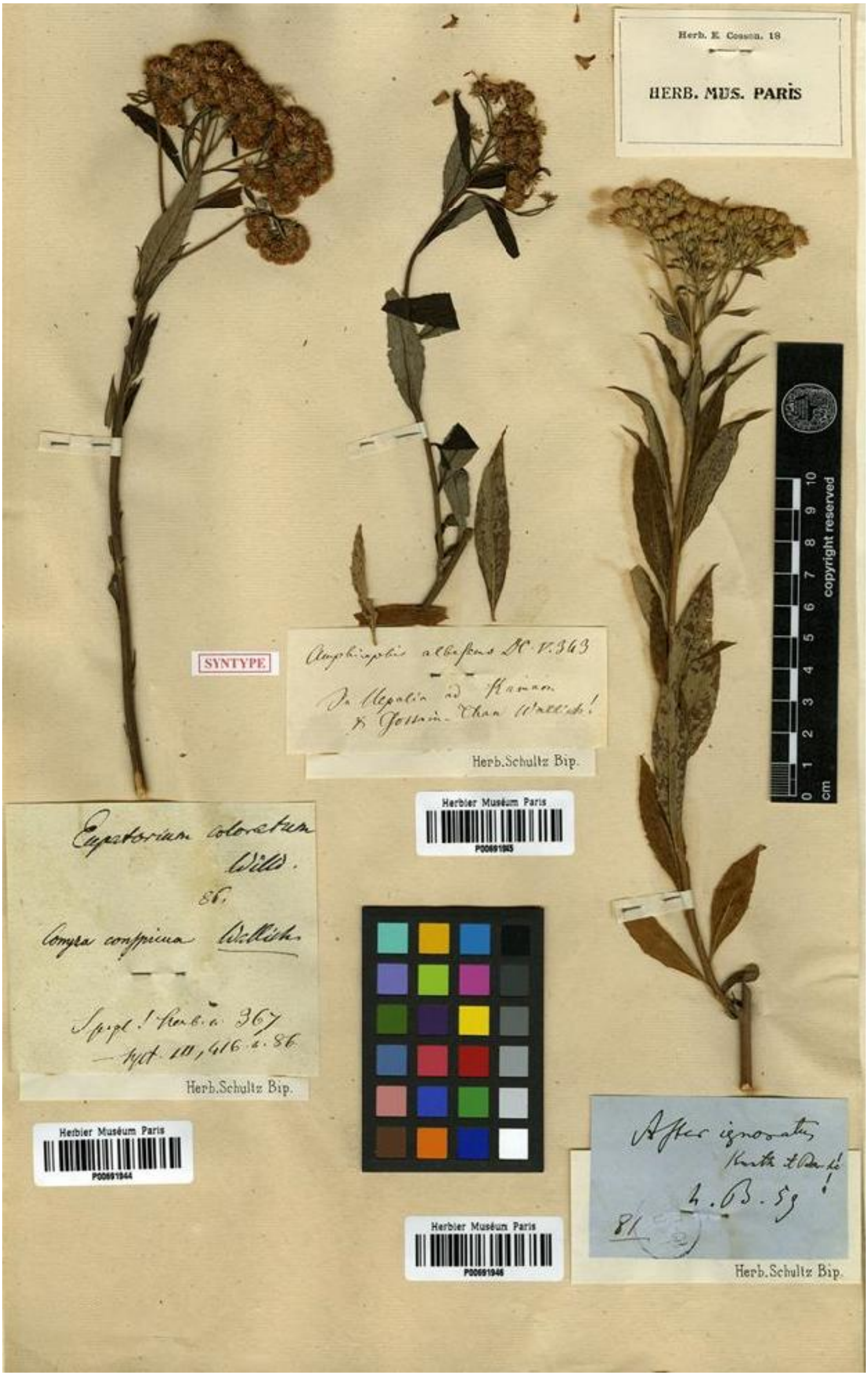

figure S2. ***Homostylium albescens*** (DC.) Z.X.Fu, **comb. nov.** = *Aster ignoratus* Kunth et Bouche. Unknown place, Herb, Schultz Bip. 81 (holotype, P 00691946!).

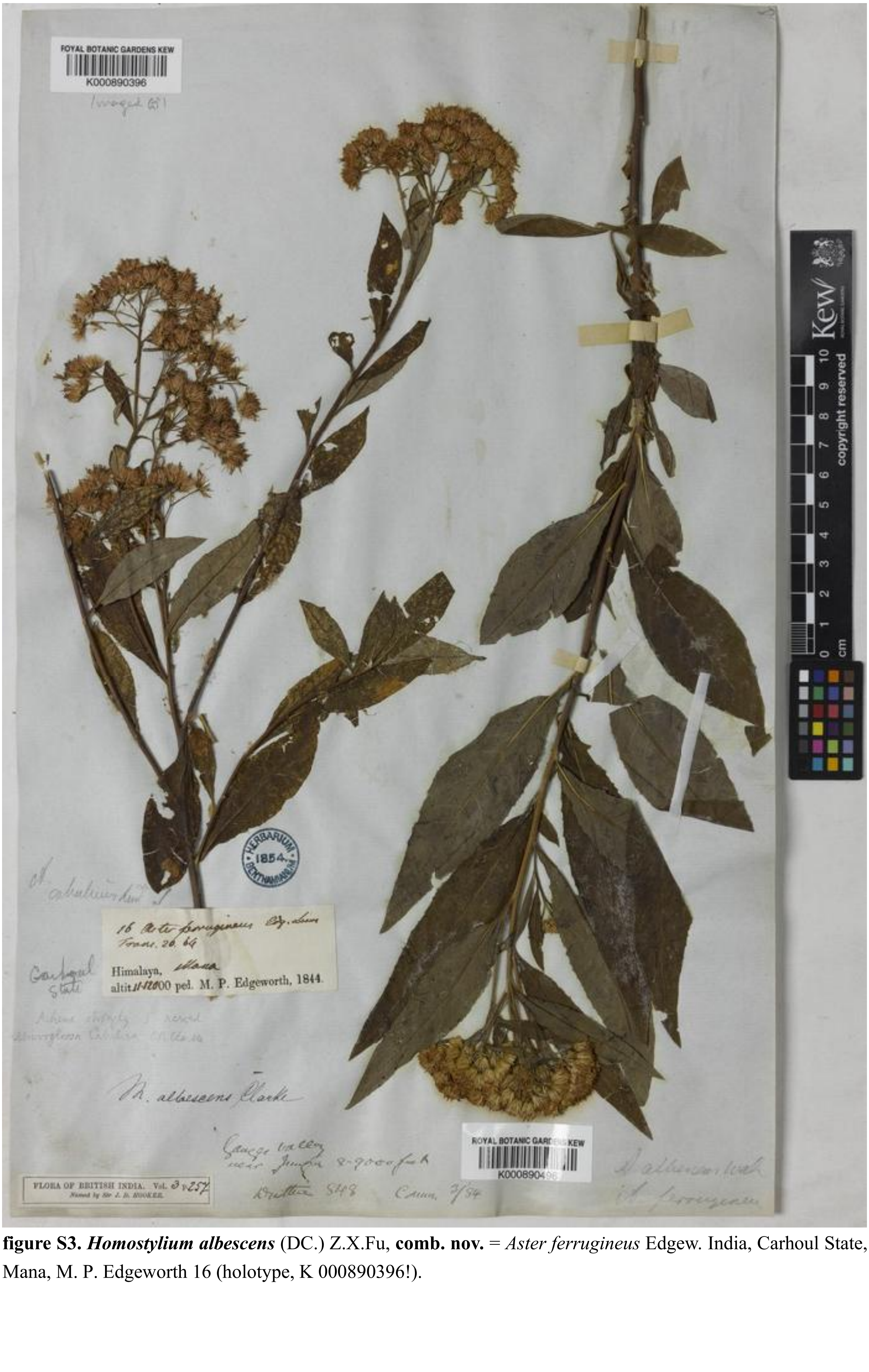

figure S3. ***Homostylium albescens*** (DC.) Z.X.Fu, **comb. nov.** = *Aster ferrugineus* Edgew. India, Carhoul State, Mana, M. P. Edgeworth 16 (holotype, K 000890396!).

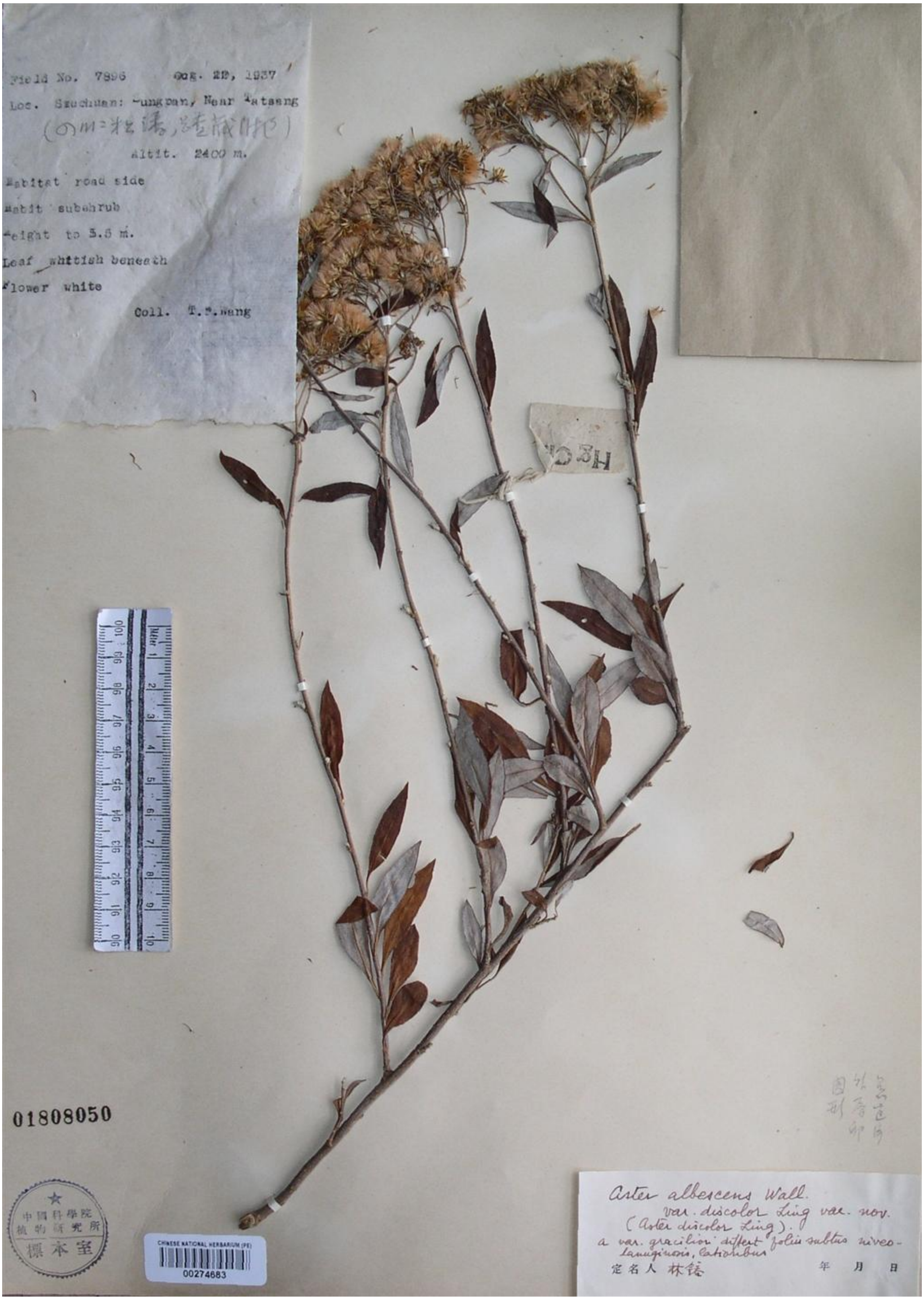

figure S4. ***Homostylium albescens*** var. ***discolor*** (Y.Ling) Z.X.Fu, **comb. nov.** ≡ *Aster albescens* var. *discolor* Y.Ling ≡ *Sinosidus albescens* var. *discolor* (Y.Ling) G.L.Nesom. China, Sichuan, Songpan, alt. 2400m, Roadside, 22 Oct 1937, T. P. Wang 7896 (holotype, PE 00274683!).

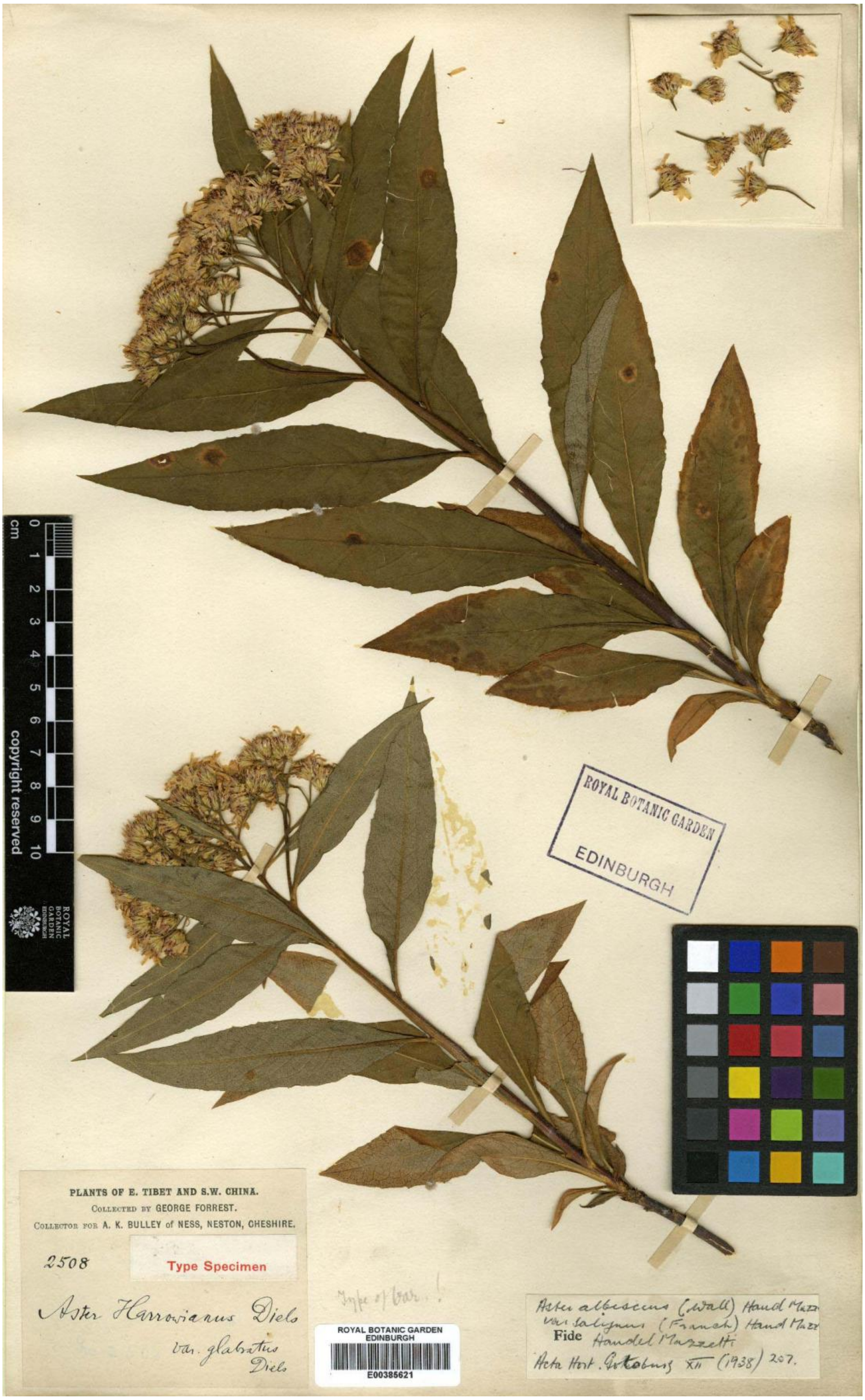

figure S5. ***Homostylium albescens*** var. ***glabratum*** (Diels) Z.X.Fu, **comb. nov.** ≡ *Aster harrowianus* var. *glabratus* Diels ≡ *Aster albescens* var. *glabratus* (Diels) Boufford & Y.S.Chen ≡ *Sinosidus albescens* var. *glabratus* (Diels) G.L.Nesom. China, Yunnan, Lijiang, shady, rocky situations, side valleys on the eastern flank, Lat. 27'15 N, alt. 9500-11000 ft, July 1906, G. Forrest 2508 (holotype, E 00385621!).

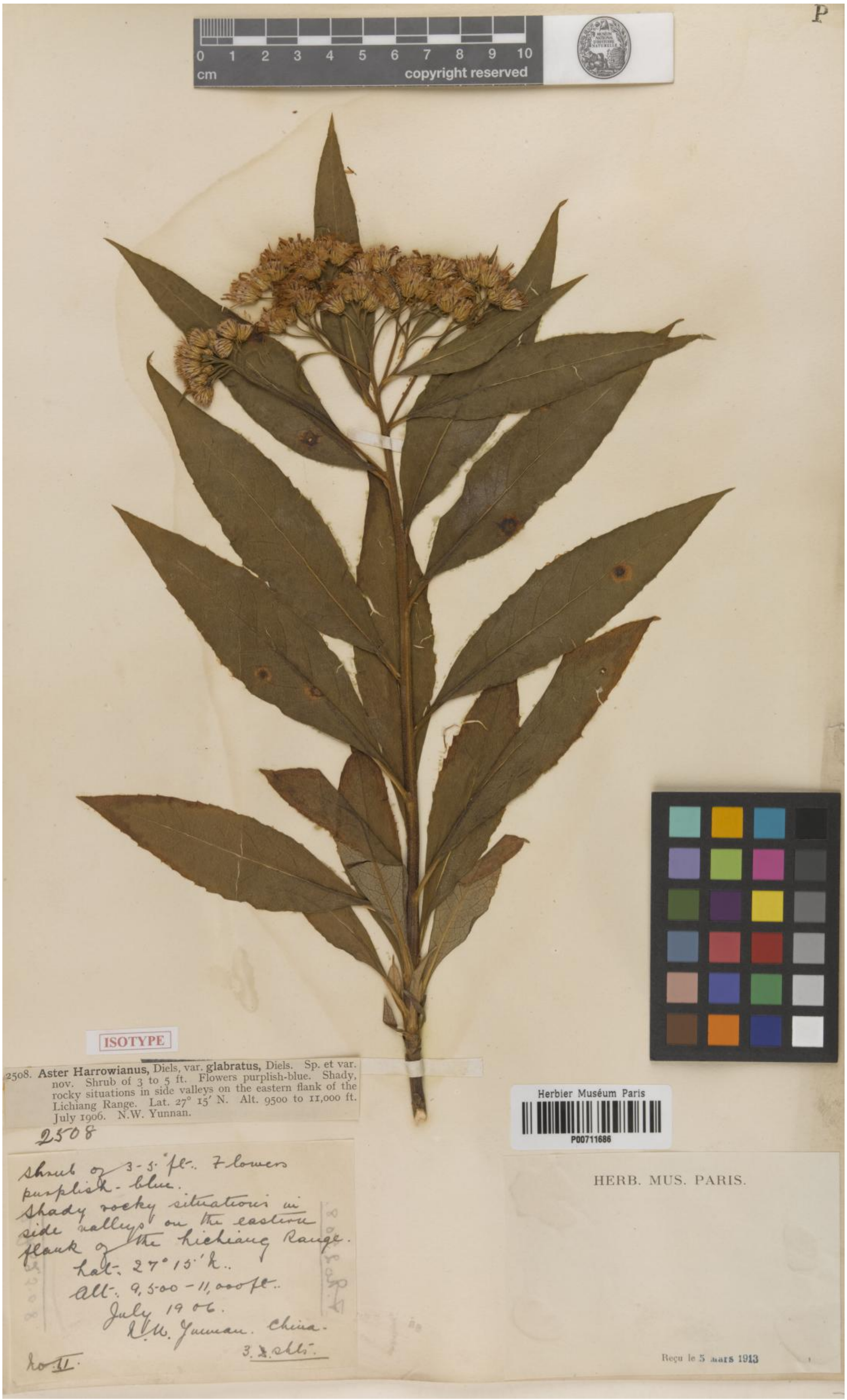

figure S6. ***Homostylium albescens*** var. ***glabratum*** (Diels) Z.X.Fu, **comb. nov.** ≡ *Aster harrowianus* var. *glabratus* Diels ≡ *Aster albescens* var. *glabratus* (Diels) Boufford & Y.S.Chen ≡ *Sinosidus albescens* var. *glabratus* (Diels) G.L.Nesom. China, Yunnan, Lijiang, shady, rocky situations, side valleys on the eastern flank, Lat. 27’15 N, alt. 9500-11000 ft, July 1906, G. Forrest 2508 (isotype, P 00711686!).

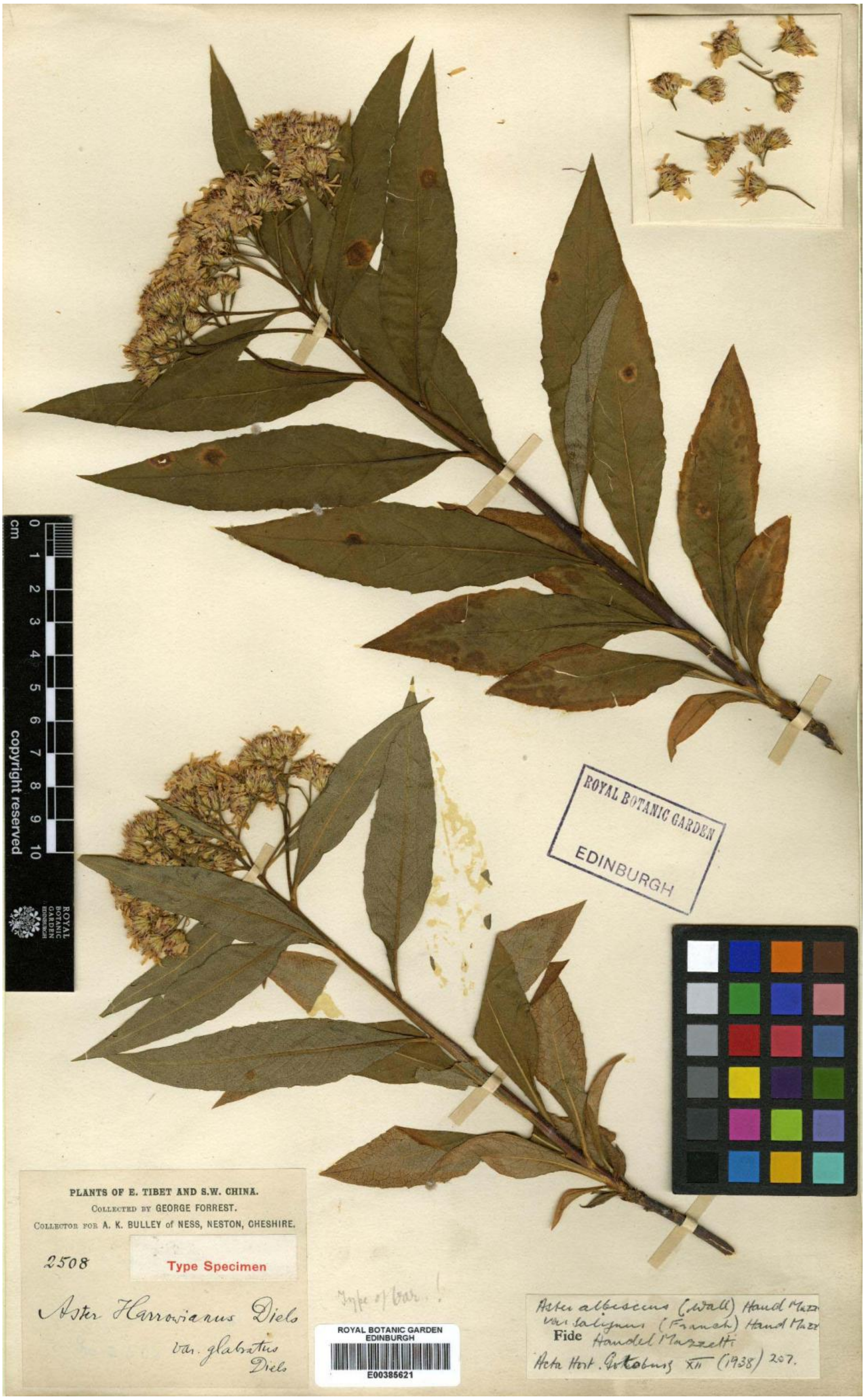

figure S7. ***Homostylium albescens*** var. ***salignum*** (Franch.) Z.X.Fu, **comb. nov.** ≡ *Inula cuspidata* var. *saligna* Franch. ≡ *Aster albescens* var. *salignus* (Franch.) Hand.-Mazz. India, George Forrest 2508 (lectotype, designated here, E 00385621!)

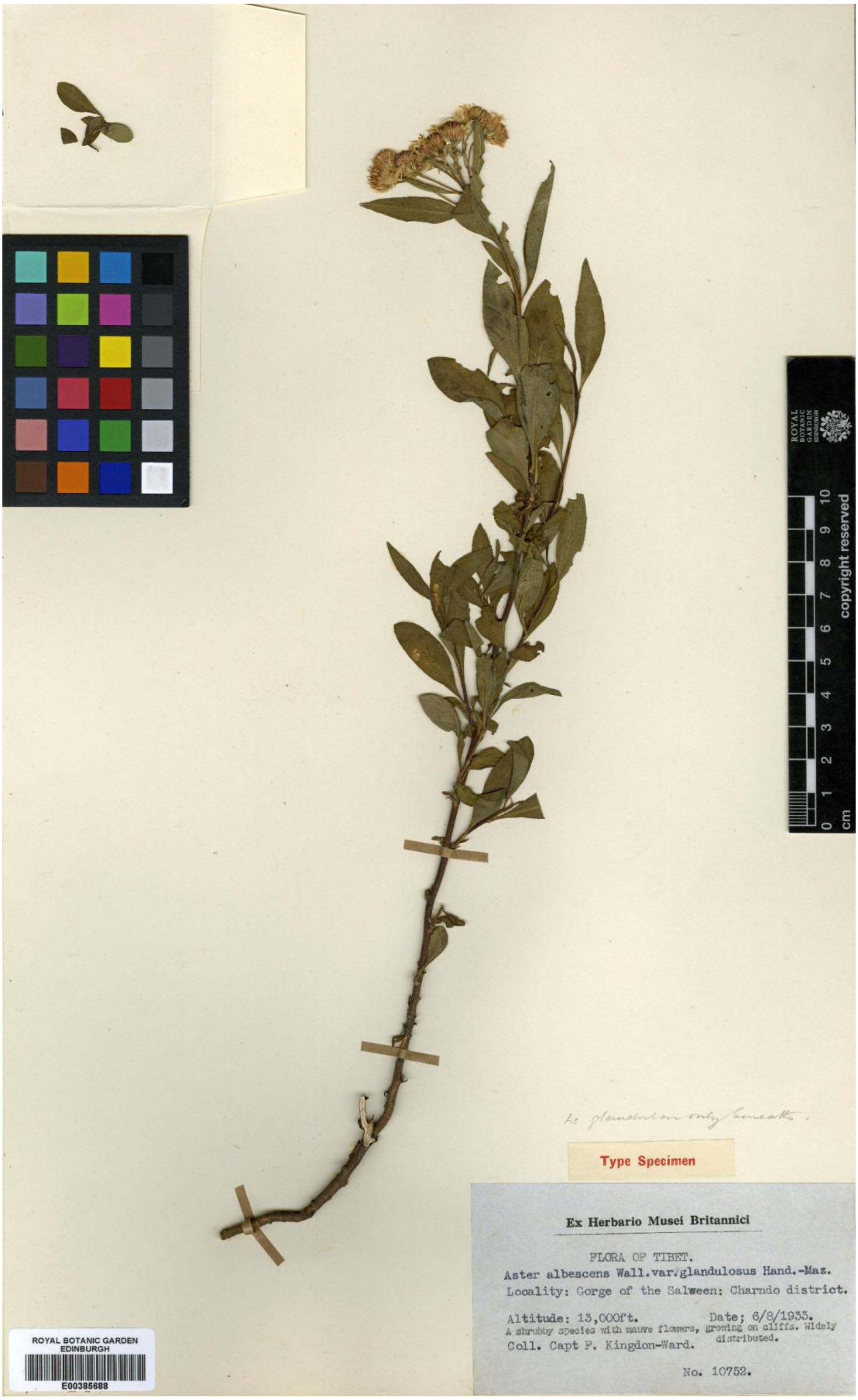

figure S8. ***Homostylium albescens*** var. ***glandulosum*** (Hand.-Mazz.) Z.X.Fu, **comb. nov.** ≡ *Aster albescens* var. *glandulosus* Hand.-Mazz. ≡ *Sinosidus albescens* var. *glandulosus* (Hand.-Mazz.) G.L.Nesom. China, Xizang, Chamdo, alt. 13000ft, 6 August 1933, F. Kingdon-Ward 10752 (holotype, E 00385688!).

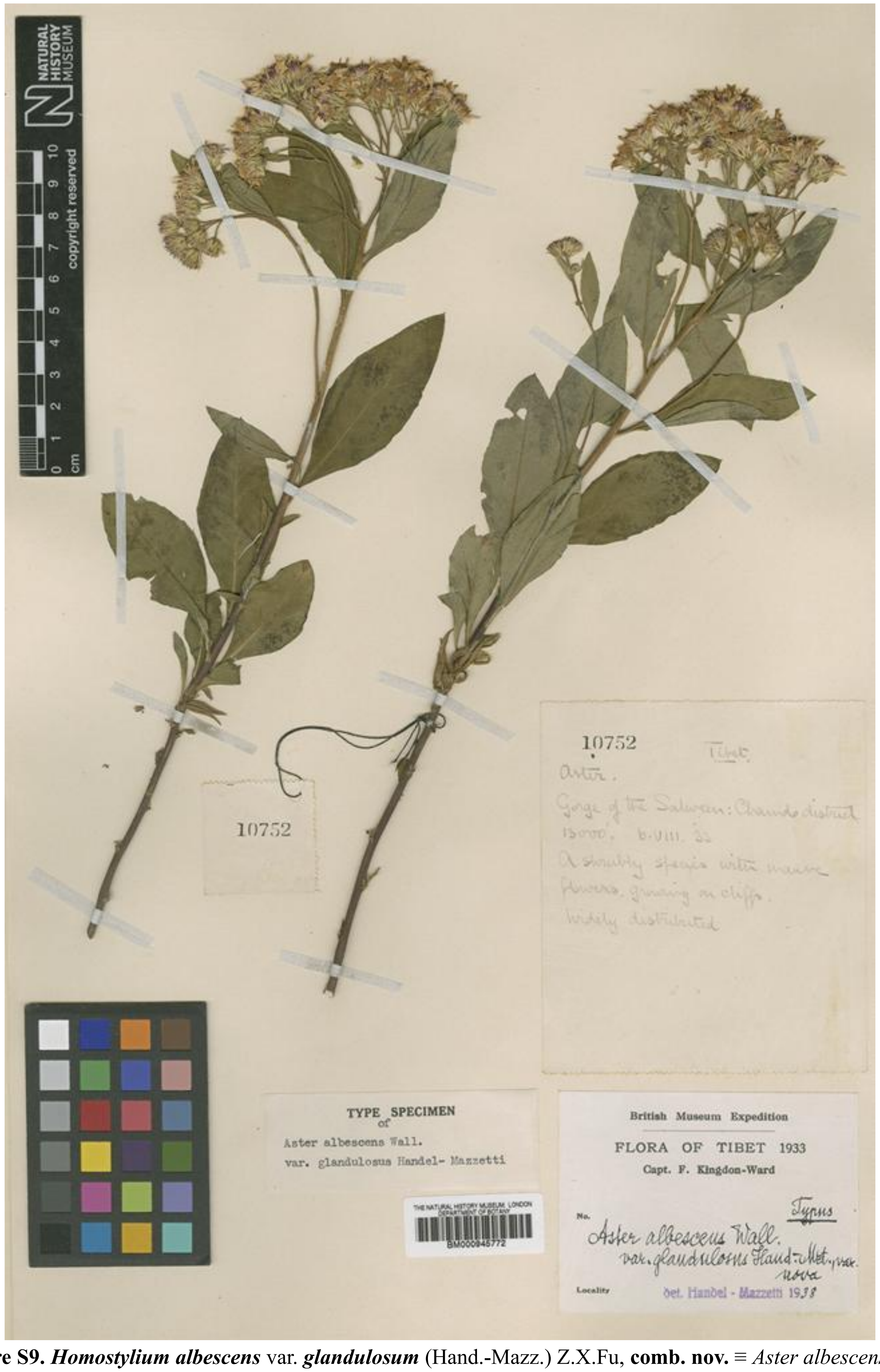

figure S9. ***Homostylium albescens*** var. ***glandulosum*** (Hand.-Mazz.) Z.X.Fu, **comb. nov.** ≡ *Aster albescens* var. *glandulosus* Hand.-Mazz. ≡ *Sinosidus albescens* var. *glandulosus* (Hand.-Mazz.) G.L.Nesom. China, Xizang, Chamdo, alt. 13000ft, 6 August 1933, F. Kingdon-Ward 10752 (isotype, BM !).

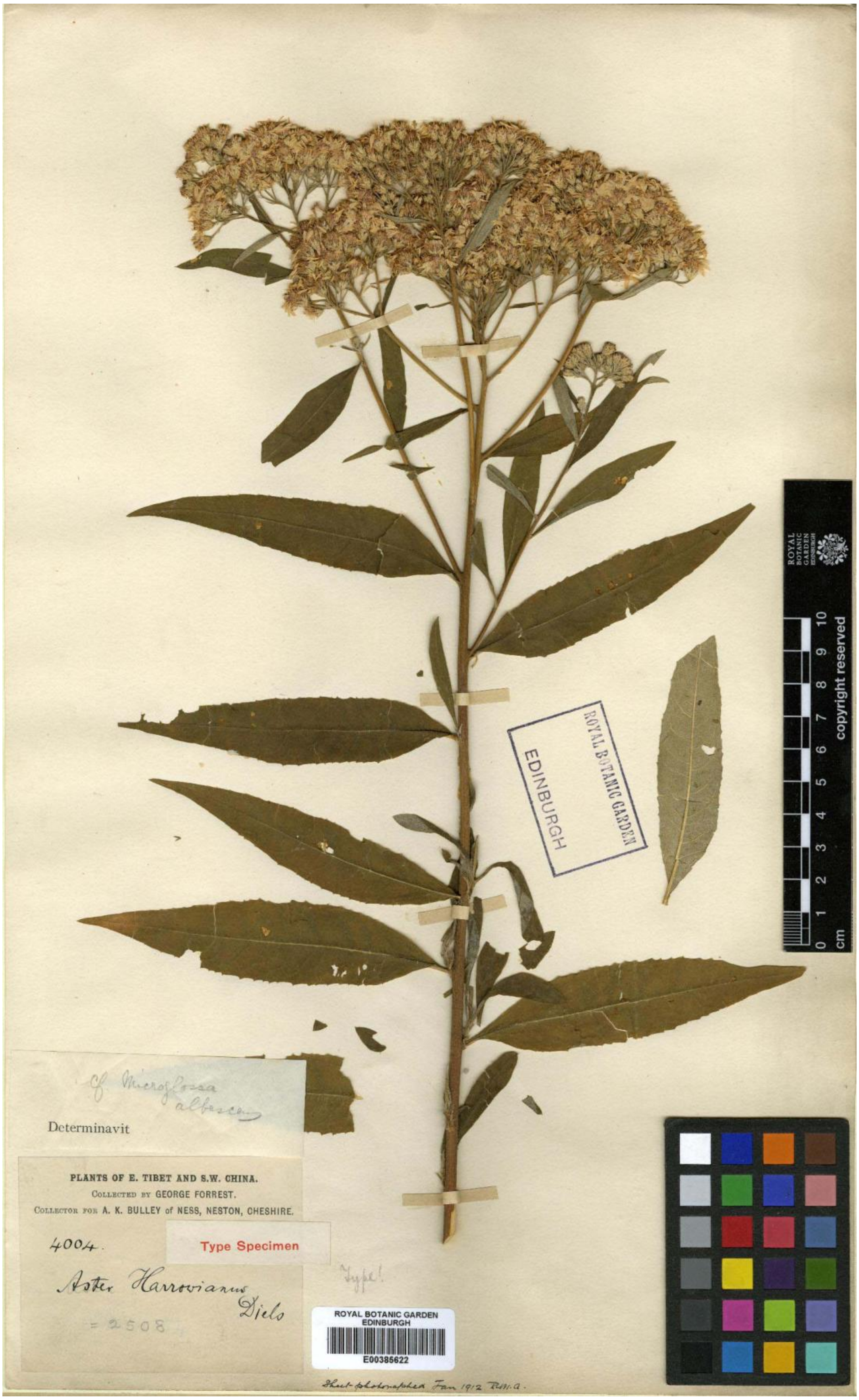

figure S10. ***Homostylium albescens*** var. ***harrowianum*** (Diels) Z.X.Fu, **comb. nov.** = *Aster harrowianus* Diels. China, Yunnan, Dali, Moist, rocky situations, side valleys on the eastern flank of the Tali Range, lat. 25' 40 N, alt. 10000-11000 ft, Sept. 1906, G. Forrest 4004 (holotype, E 00385622!).

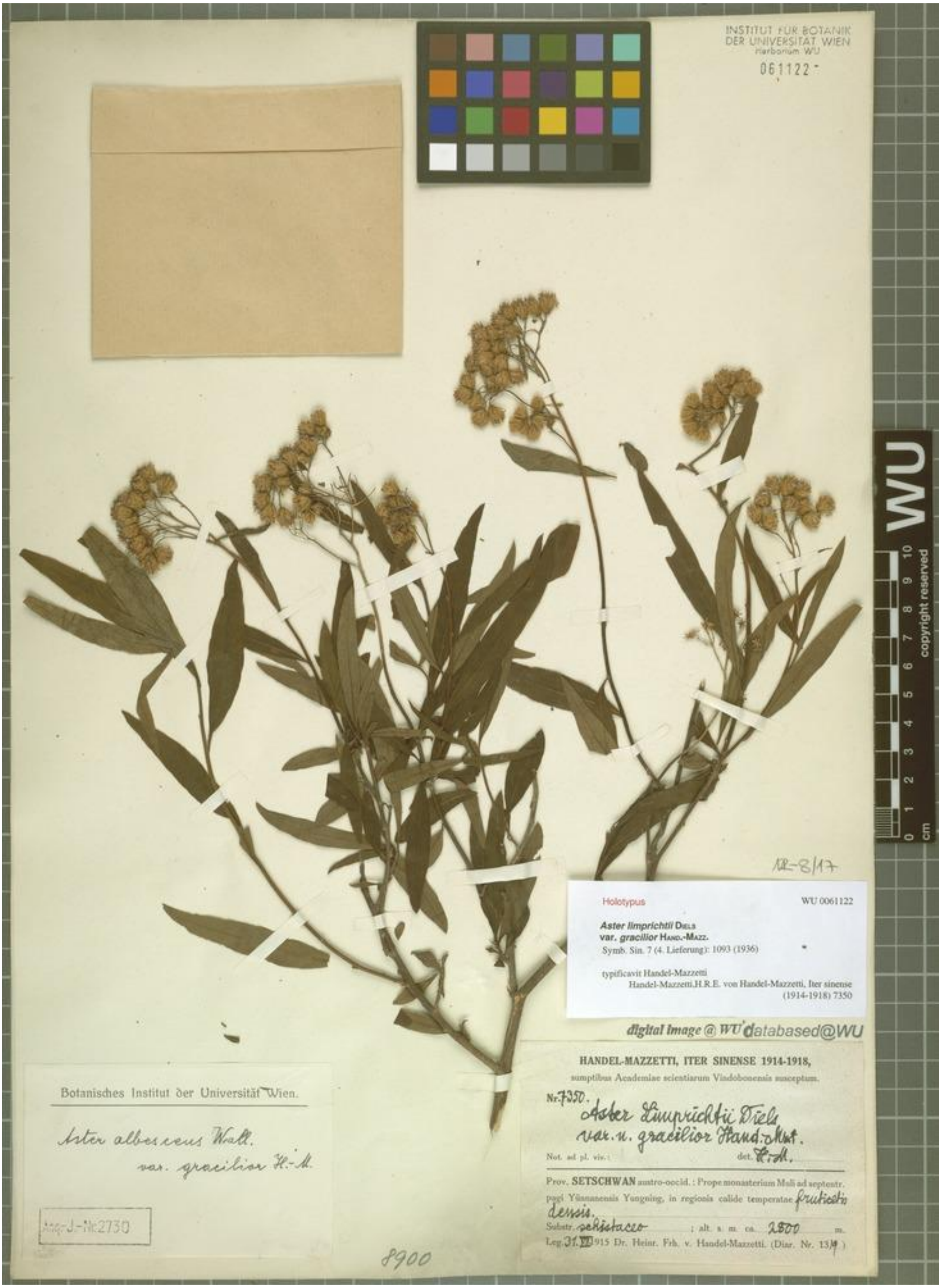

figure S11. ***Homostylium albescens*** var. ***harrowianum*** (Diels) Z.X.Fu, **comb. nov.** ≡ *Aster limprichtii* var. *gracilior* Hand.-Mazz. ≡ *Aster albescens* var. *gracilior* (Hand.-Mazz.) Hand.-Mazz. ≡ *Sinosidus albescens* var. *gracilior* (Hand.-Mazz.) G.L.Nesom. China, Sichuan, Muli, alt. 2800m, 31 July 1915, Hand.-Mazz. 7350 (holotype, WU 0061122!).

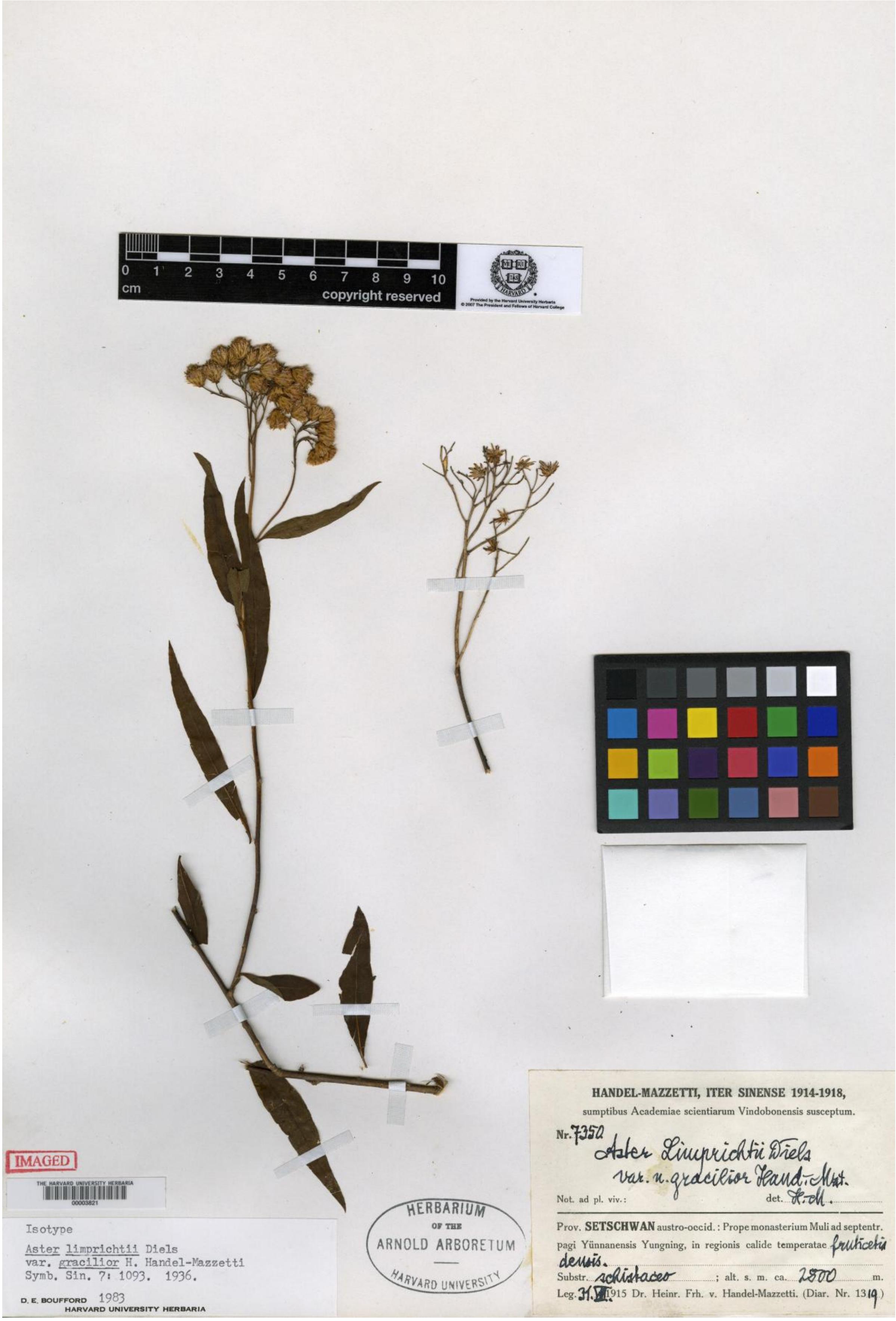

figure S12. ***Homostylium albescens*** var. ***harrowianum*** (Diels) Z.X.Fu, **comb. nov.** ≡ *Aster limprichtii* var. *gracilior* Hand.-Mazz. ≡ *Aster albescens* var. *gracilior* (Hand.-Mazz.) Hand.-Mazz. ≡ *Sinosidus albescens* var. *gracilior* (Hand.-Mazz.) G.L.Nesom. China, Sichuan, Muli, alt. 2800m, 31 July 1915, Hand.-Mazz. 7350 (isotype, A 00003821!).

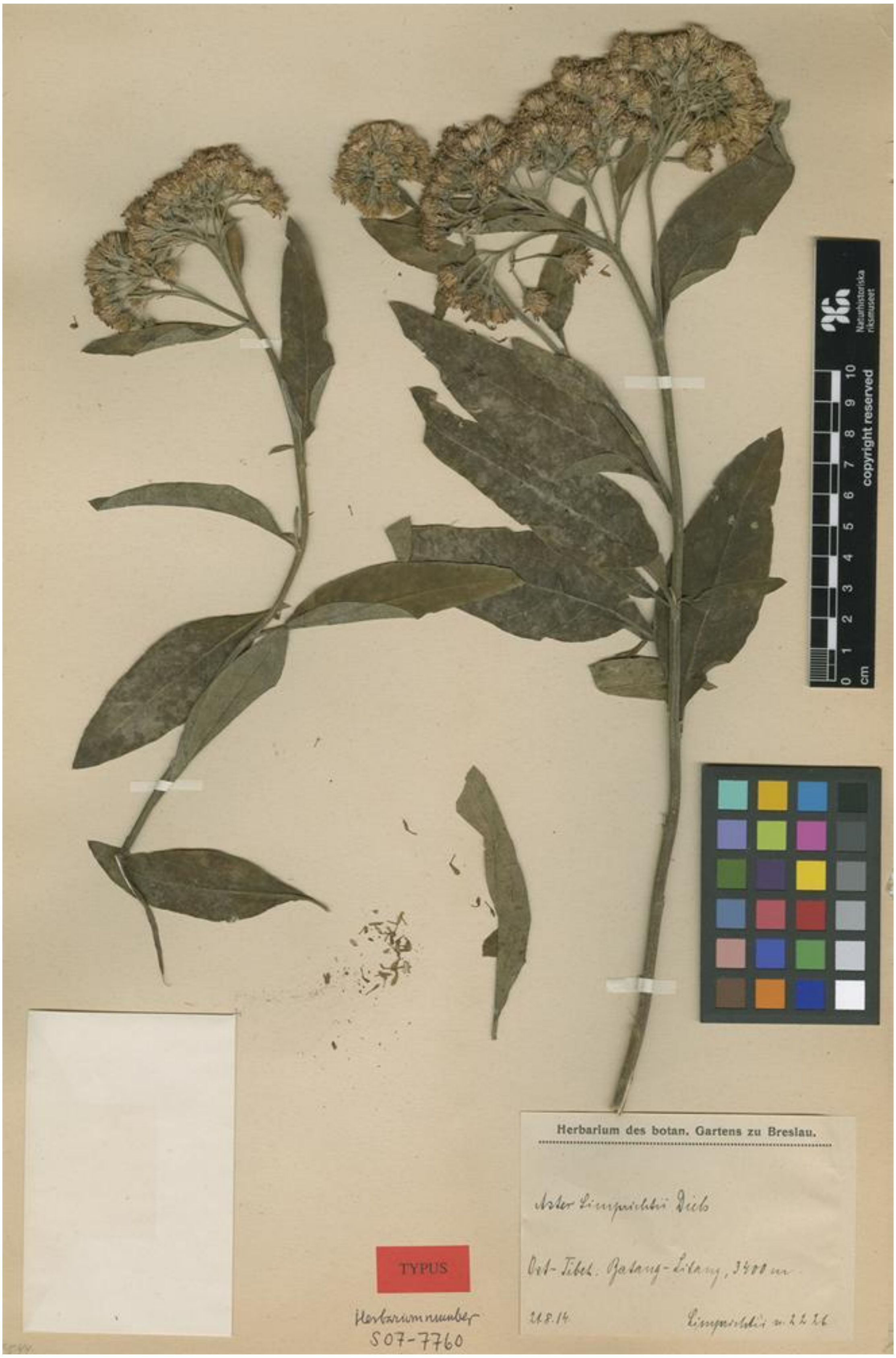

figure S13. ***Homostylium albescens*** var. ***limprichtii*** (Diels) Z.X.Fu, **comb. nov.** ≡ *Aster limprichtii* Diels ≡ *Aster albescens* var. *limprichtii* (Diels) Hand.-Mazz. ≡ *Sinosidus albescens* var. *limprichtii* (Diels) G.L.Nesom. China, Sichuan, Batang-Litang, alt. 3400 m, 21 August 1914, H. W. Limpricht 2226 (lectotype, designated here, WRSL S07-7760!).

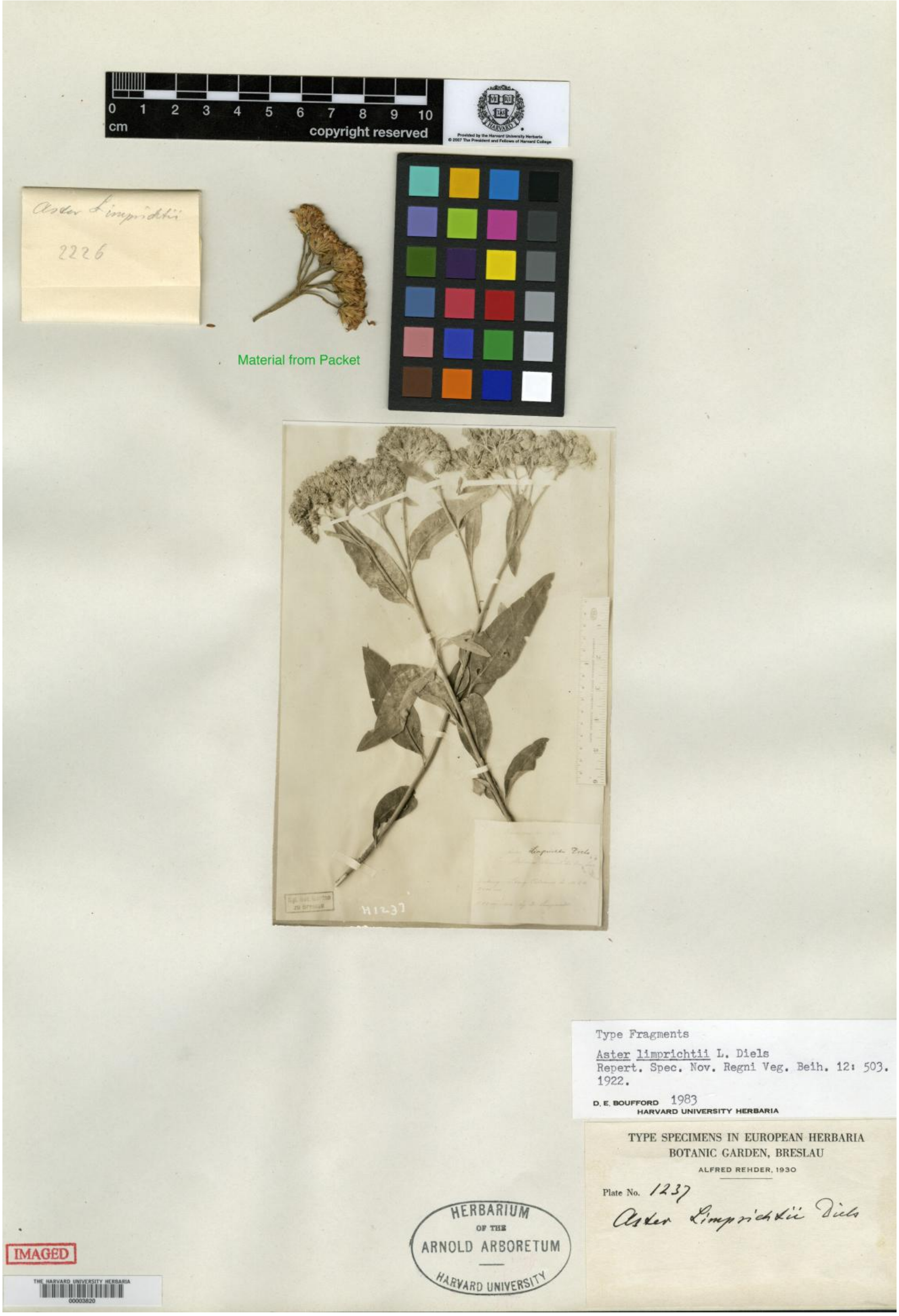

figure S14. ***Homostylium albescens*** var. ***limprichtii*** (Diels) Z.X.Fu, **comb. nov.** ≡ *Aster limprichtii* Diels ≡ *Aster albescens* var. *limprichtii* (Diels) Hand.-Mazz. ≡ *Sinosidus albescens* var. *limprichtii* (Diels) G.L.Nesom. China, Sichuan, Batang-Litang, alt. 3400 m, 21 August 1914, H. W. Limpricht 2226 (isolectotype, A 00003820!).

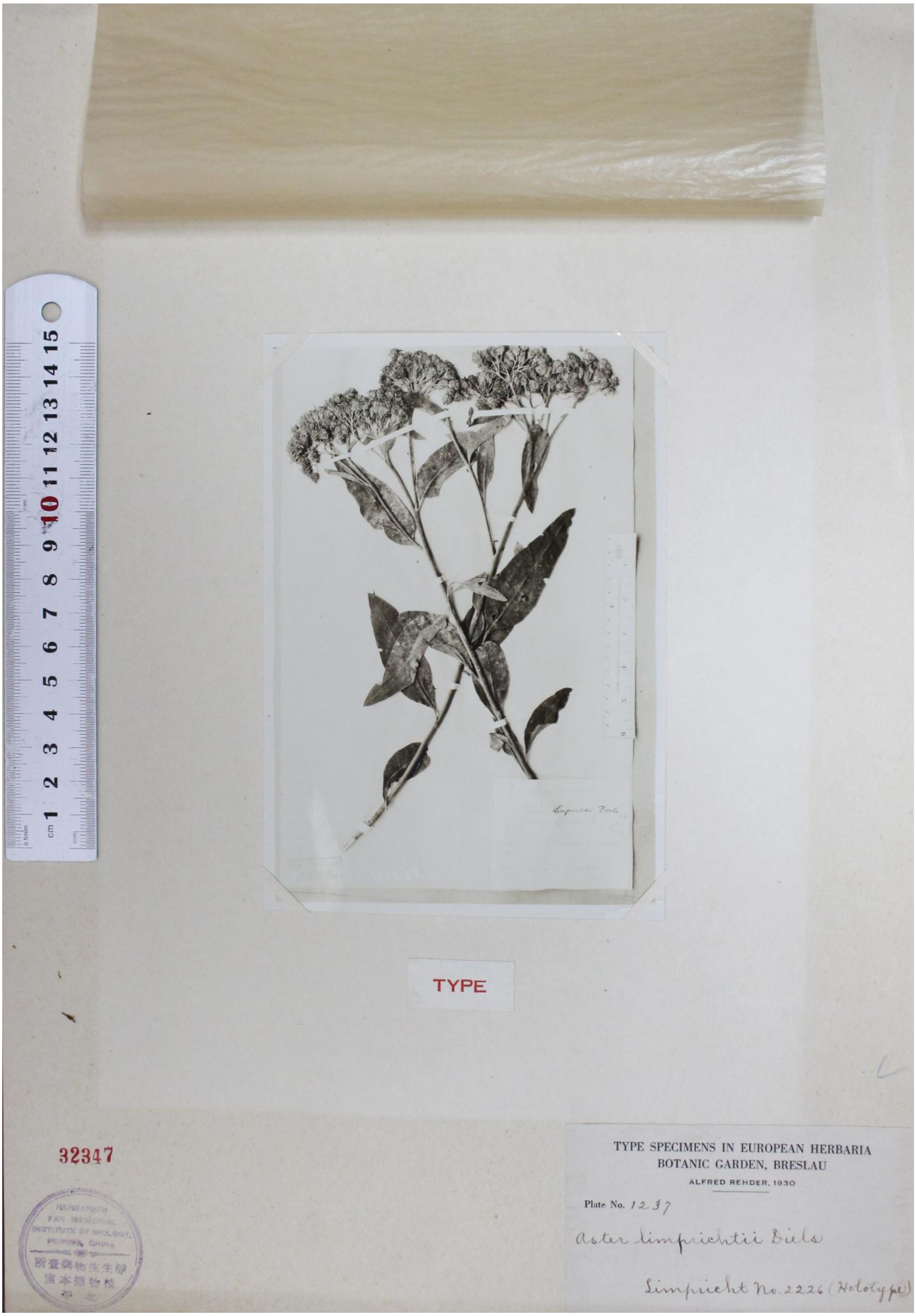

figure S15. ***Homostylium albescens*** var. ***limprichtii*** (Diels) Z.X.Fu, **comb. nov.** ≡ *Aster limprichtii* Diels ≡ *Aster albescens* var. *limprichtii* (Diels) Hand.-Mazz. ≡ *Sinosidus albescens* var. *limprichtii* (Diels) G.L.Nesom. China, Sichuan, Batang-Litang, alt. 3400 m, 21 August 1914, H. W. Limpricht 2226 (isolectotype, PE no. 32347!).

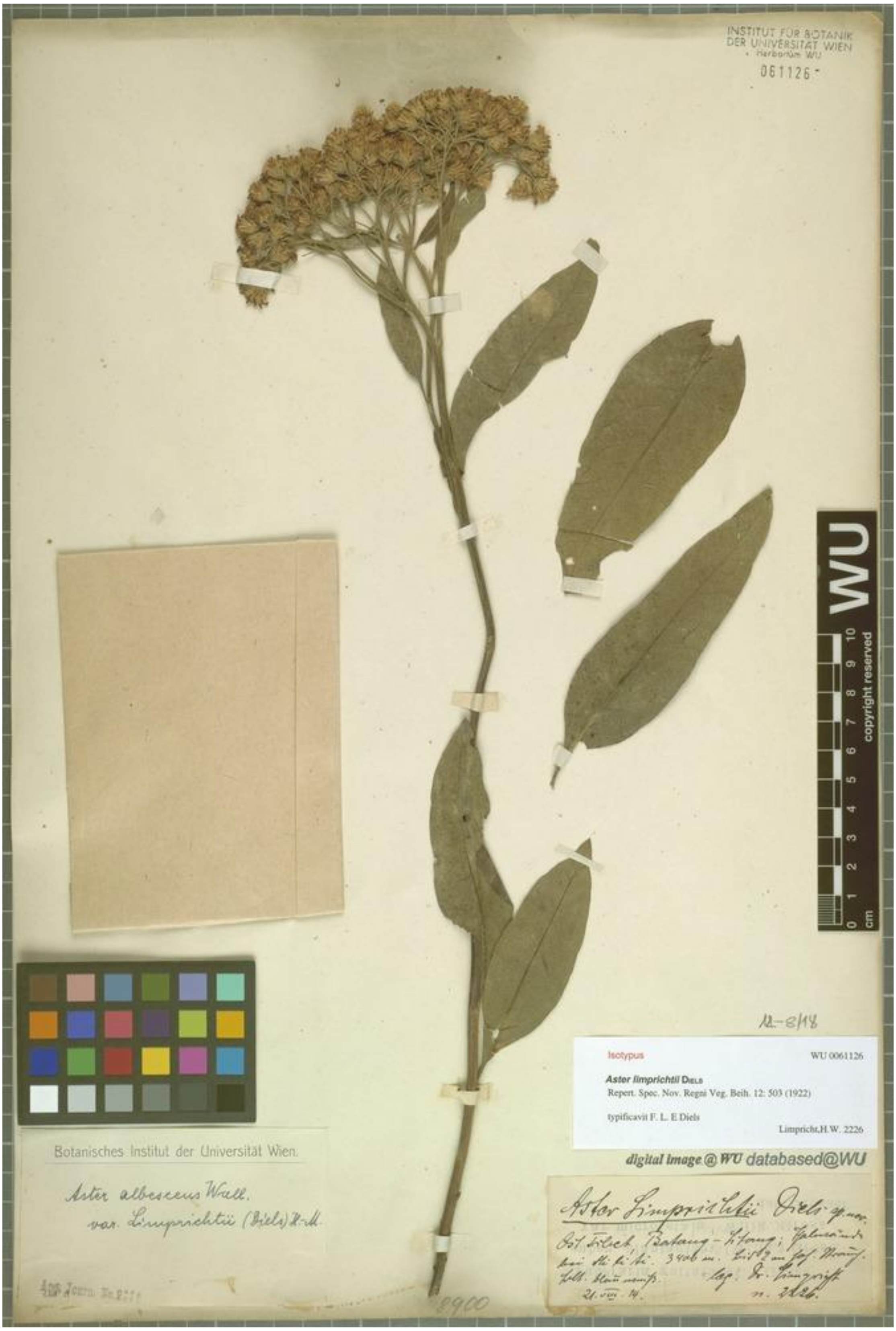

figure S16. ***Homostylium albescens*** var. ***limprichtii*** (Diels) Z.X.Fu, **comb. nov.** ≡ *Aster limprichtii* Diels ≡ *Aster albescens* var. *limprichtii* (Diels) Hand.-Mazz. ≡ *Sinosidus albescens* var. *limprichtii* (Diels) G.L.Nesom. China, Sichuan, Batang-Litang, alt. 3400 m, 21 August 1914, H. W. Limpricht 2226 (isolectotype, WU 0061126!).

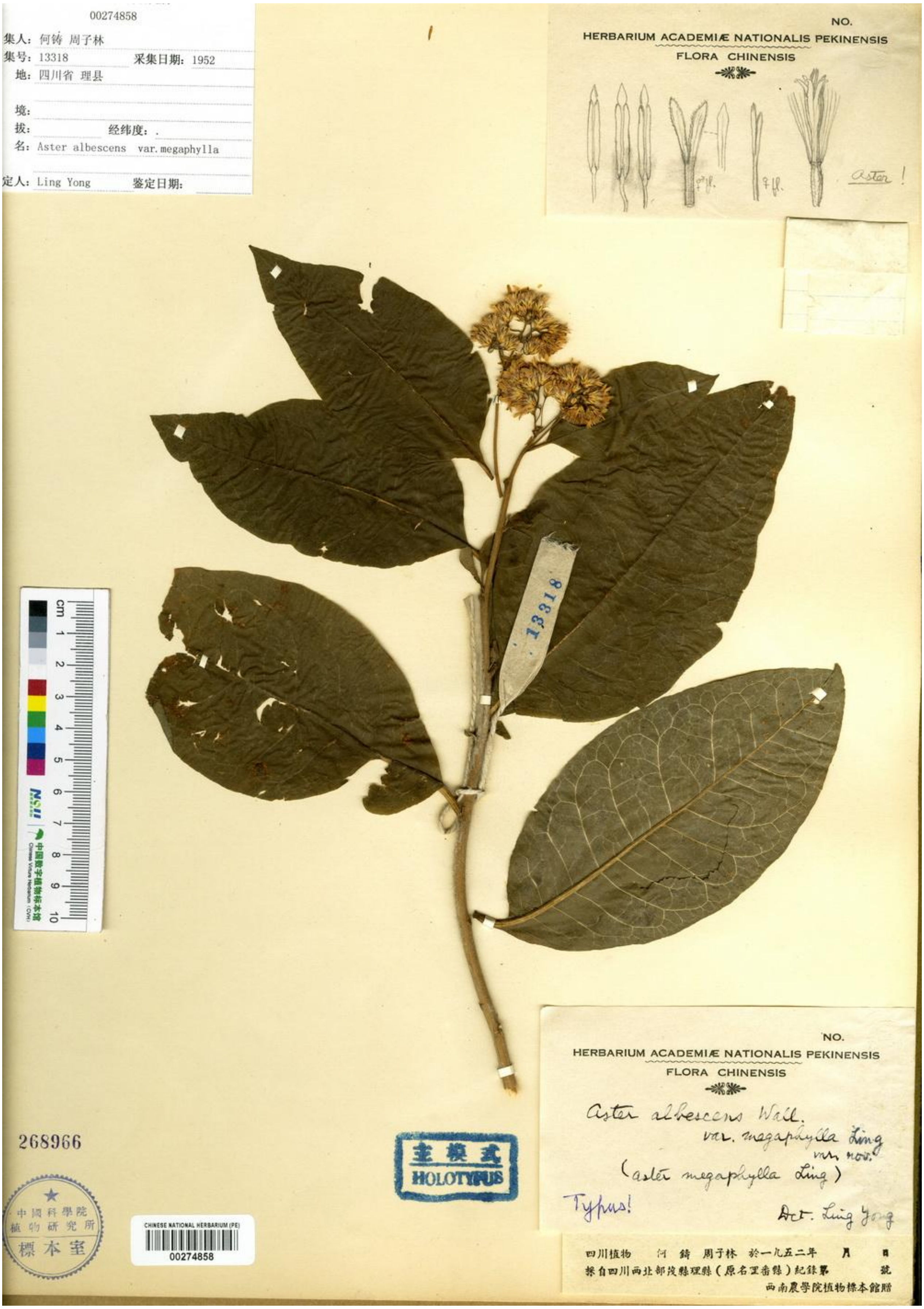

figure S17. ***Homostylium albescens*** var. ***megaphyllum*** (Y.Ling) Z.X.Fu, **comb. nov.** ≡ *Aster albescens* var. *megaphyllus* Y.Ling ≡ *Sinosidus albescens* var. *megaphyllus* (Y.Ling) G.L.Nesom. China, Sichuan, Lixian, C. Ho & Z. L. Zhou 13318 (holotype, PE 00274858!).

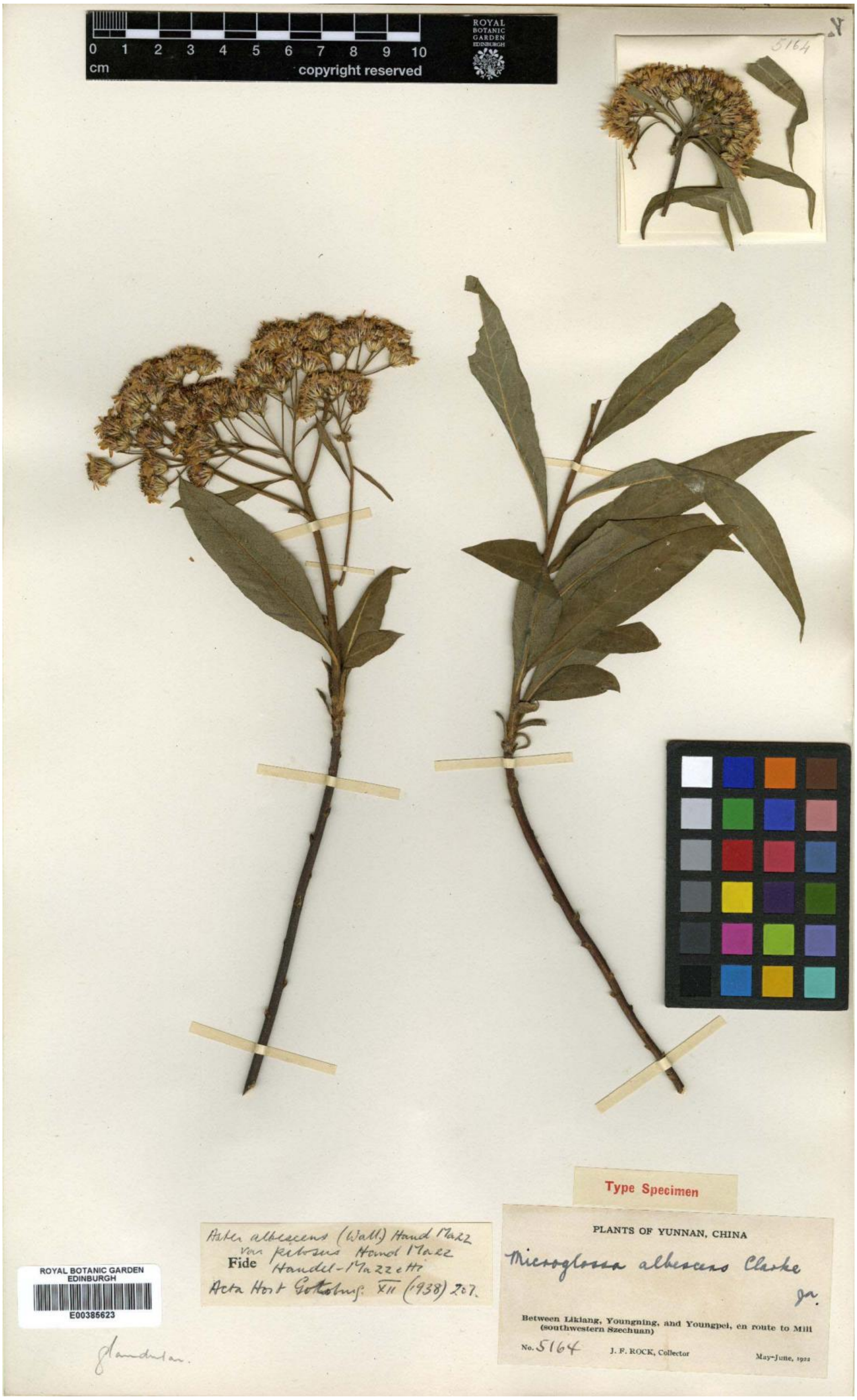

figure S18. ***Homostylium albescens*** var. ***pilosum*** (Hand.-Mazz.) Z.X.Fu, **comb. nov.** ≡ *Aster albescens* var. *pilosus* Hand.-Mazz. ≡ *Sinosidus albescens* var. *pilosus* (Hand.-Mazz.) G.L.Nesom. China, Yunnan, Lijiang, Ninglang and Yongsheng, 15 May 1922, J. F. Rock 5164 (isotype, E 00385623!).

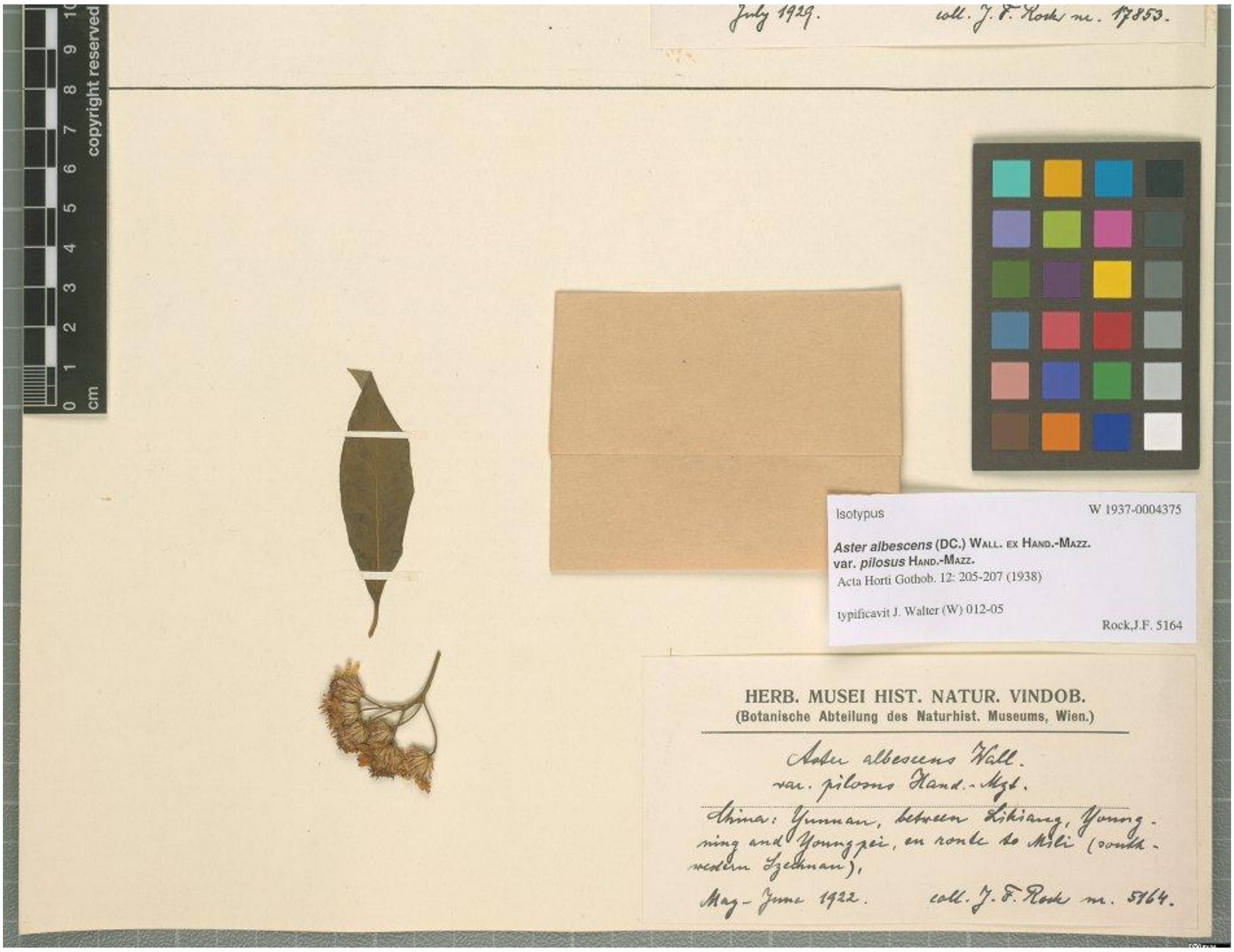

figure S19. ***Homostylium albescens*** var. ***pilosum*** (Hand.-Mazz.) Z.X.Fu, **comb. nov.** ≡ *Aster albescens* var. *pilosus* Hand.-Mazz. ≡ *Sinosidus albescens* var. *pilosus* (Hand.-Mazz.) G.L.Nesom. China, Yunnan, Lijiang, Ninglang and Yongsheng, 15 May 1922, J. F. Rock 5164 (isotype, WU 1937-0004375!).

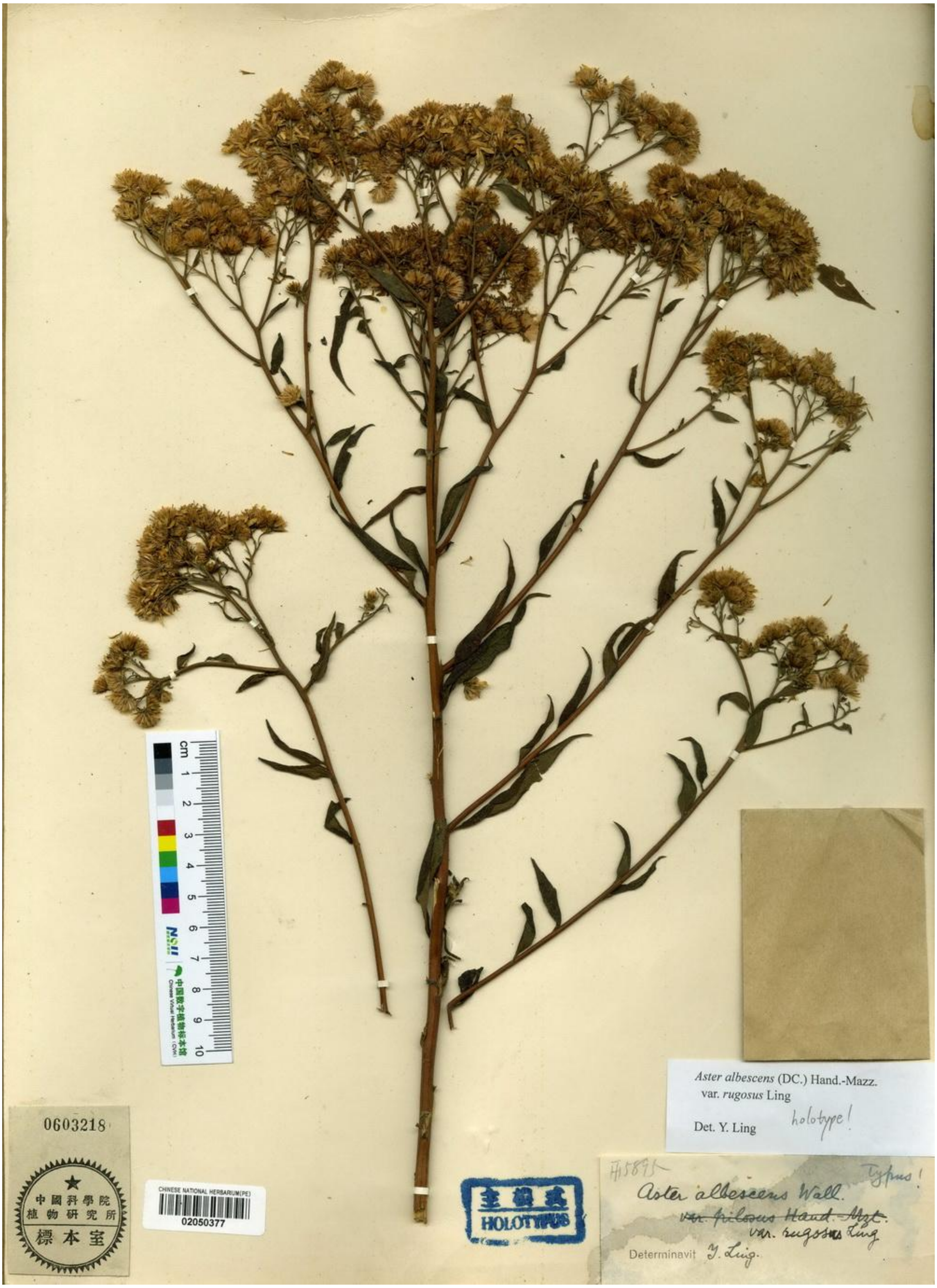

figure S20. ***Homostylium albescens*** var. ***rugosum*** (Y.Ling) Z.X.Fu, **comb. nov.** ≡ *Aster albescens* var. *rugosus* Y.Ling. China, Yunnan, Qinghua university 5895 (holotype, PE 02050377!).

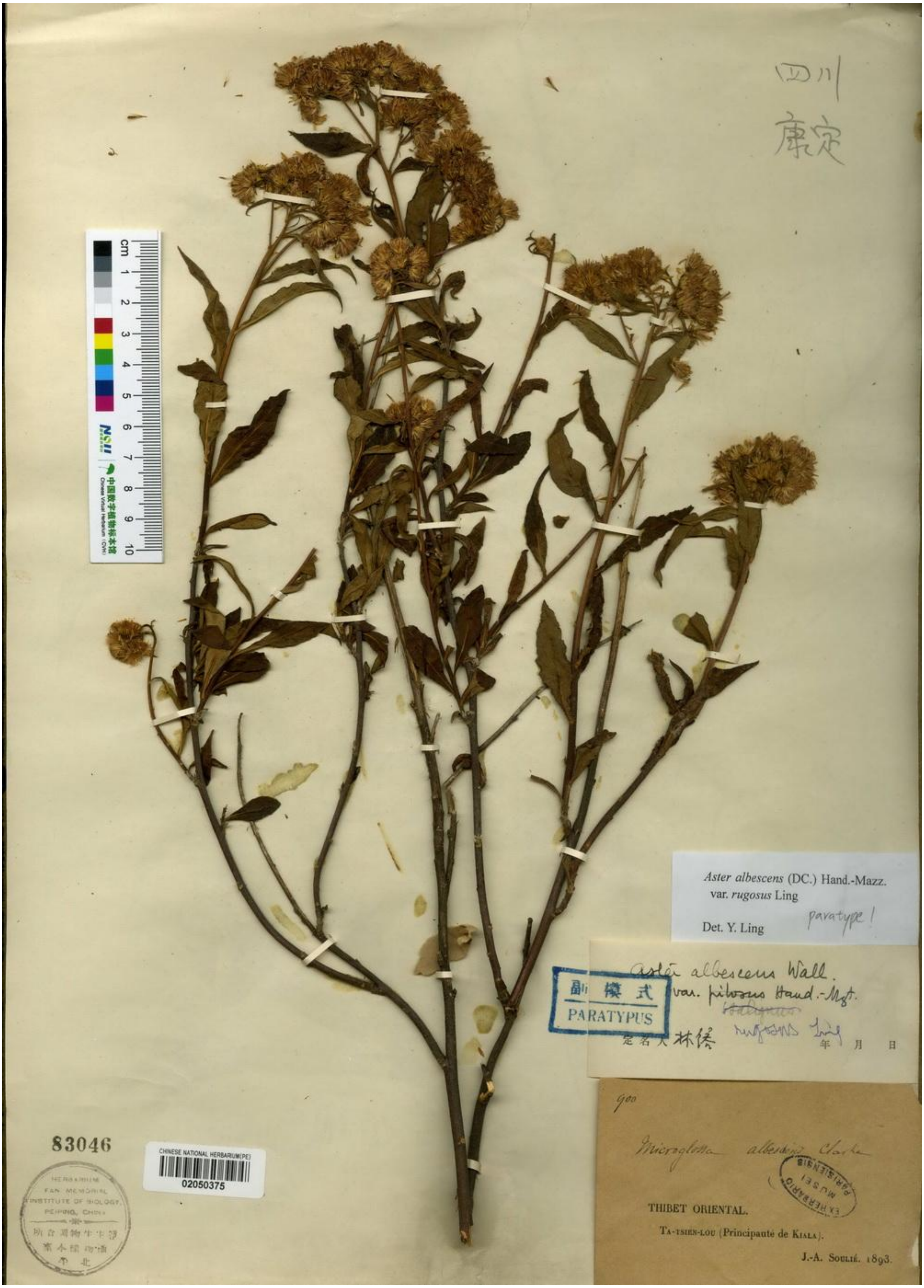

figure S21. ***Homostylium albescens*** var. ***rugosum*** (Y.Ling) Z.X.Fu, **comb. nov.** = *Sinosidus albescens* var. *rugosus* (Y.Ling) G.L.Nesom. China, Sichuan, Kangding, 1893, Soulie 900 (paratype, PE 02050375!).

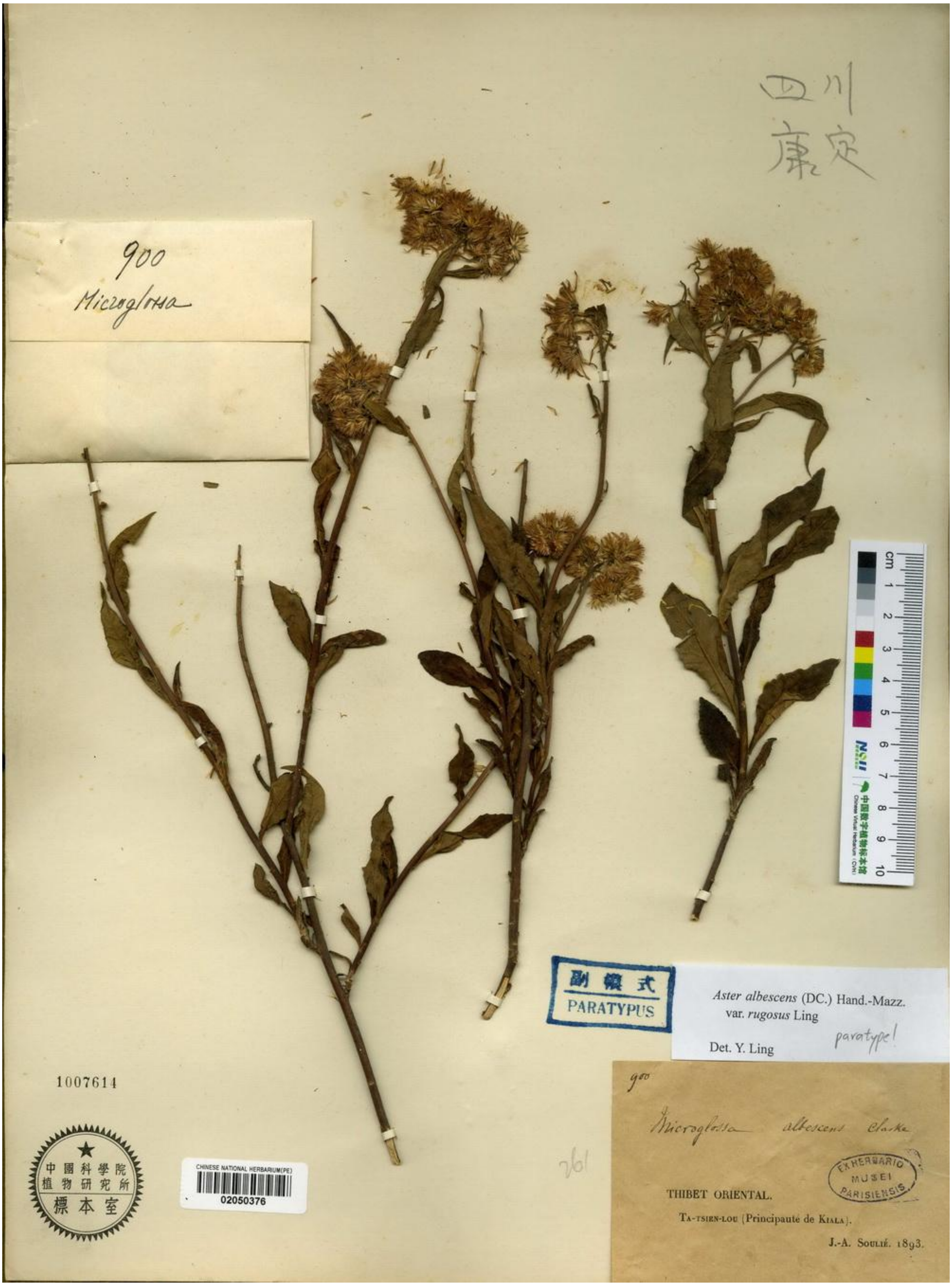

figure S22. ***Homostylium albescens*** var. ***rugosum*** (Y.Ling) Z.X.Fu, **comb. nov.** = *Sinosidus albescens* var. *rugosus* (Y.Ling) G.L.Nesom. China, Sichuan, Kangding, 1893, Soulie 900 (paratype, PE 02050376!).

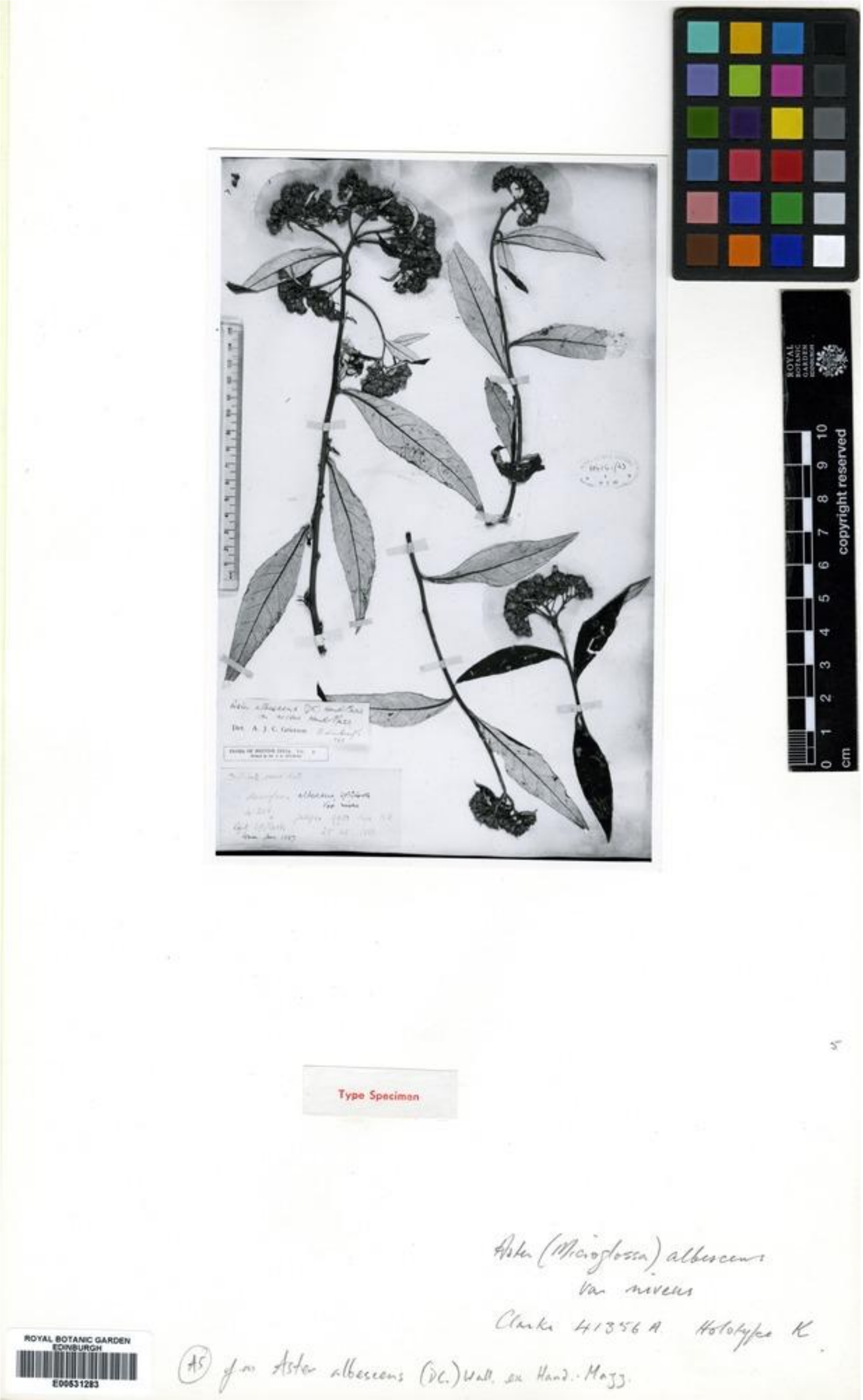

figure S23. ***Homostylium albescens*** var. ***niveum*** (Hand.-Mazz.) Z.X.Fu, **comb. nov.** ≡ *Aster albescens* var. *niveus* Hand.-Mazz. India, Sikkim, Jakpho, Napa Hill, alt. 3017 m, 25 Oct. 1885, C.B.Clarke 41356 (holotype, E 00531283!).

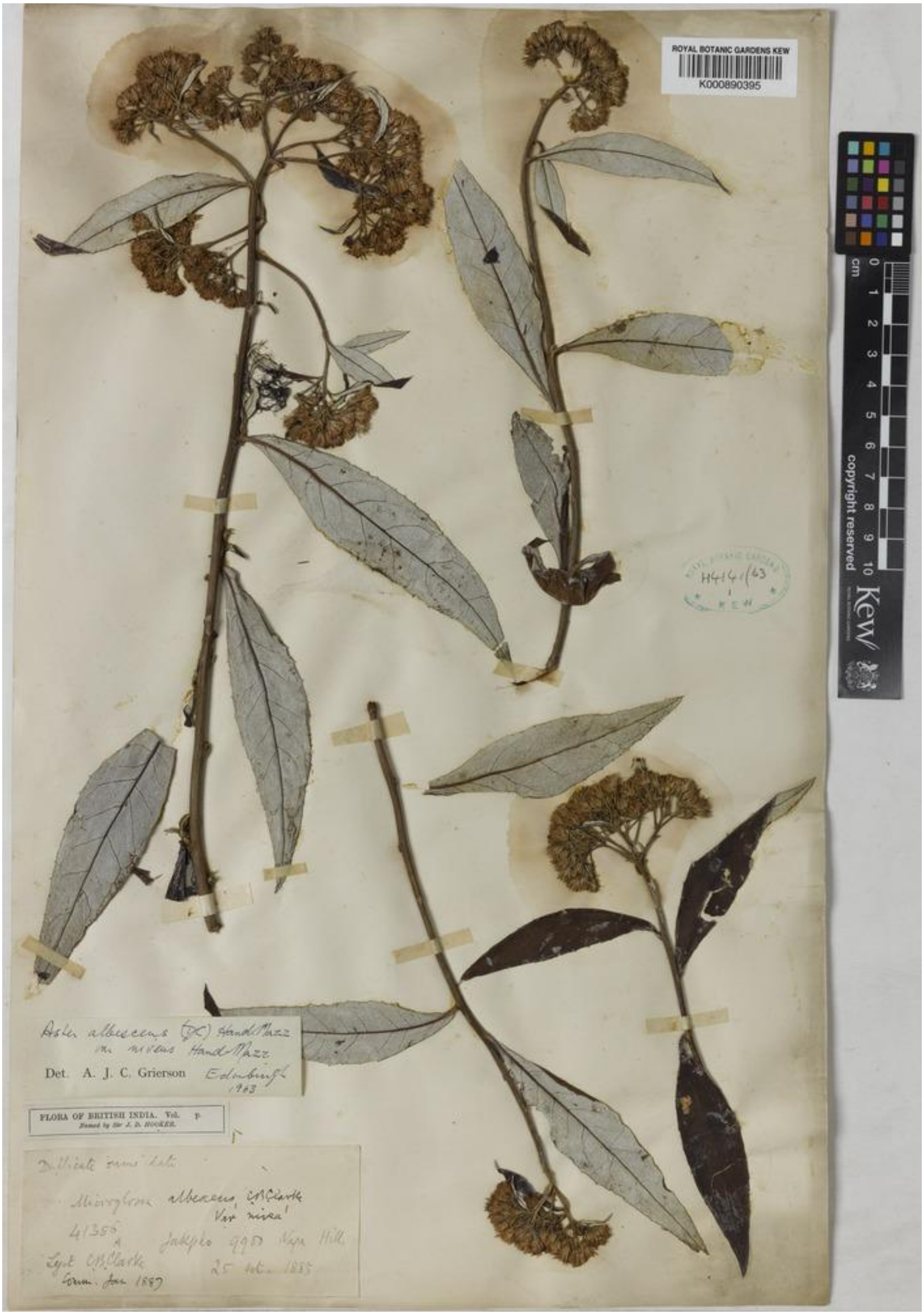

figure S24. ***Homostylium albescens*** var. ***niveum*** (Hand.-Mazz.) Z.X.Fu, **comb. nov.** ≡ *Aster albescens* var. *niveus* Hand.-Mazz. India, Sikkim, Jakpho, Napa Hill, alt 3017 m, 25 Oct. 1885, C.B.Clarke 41356 (isotype, K 00089039!).

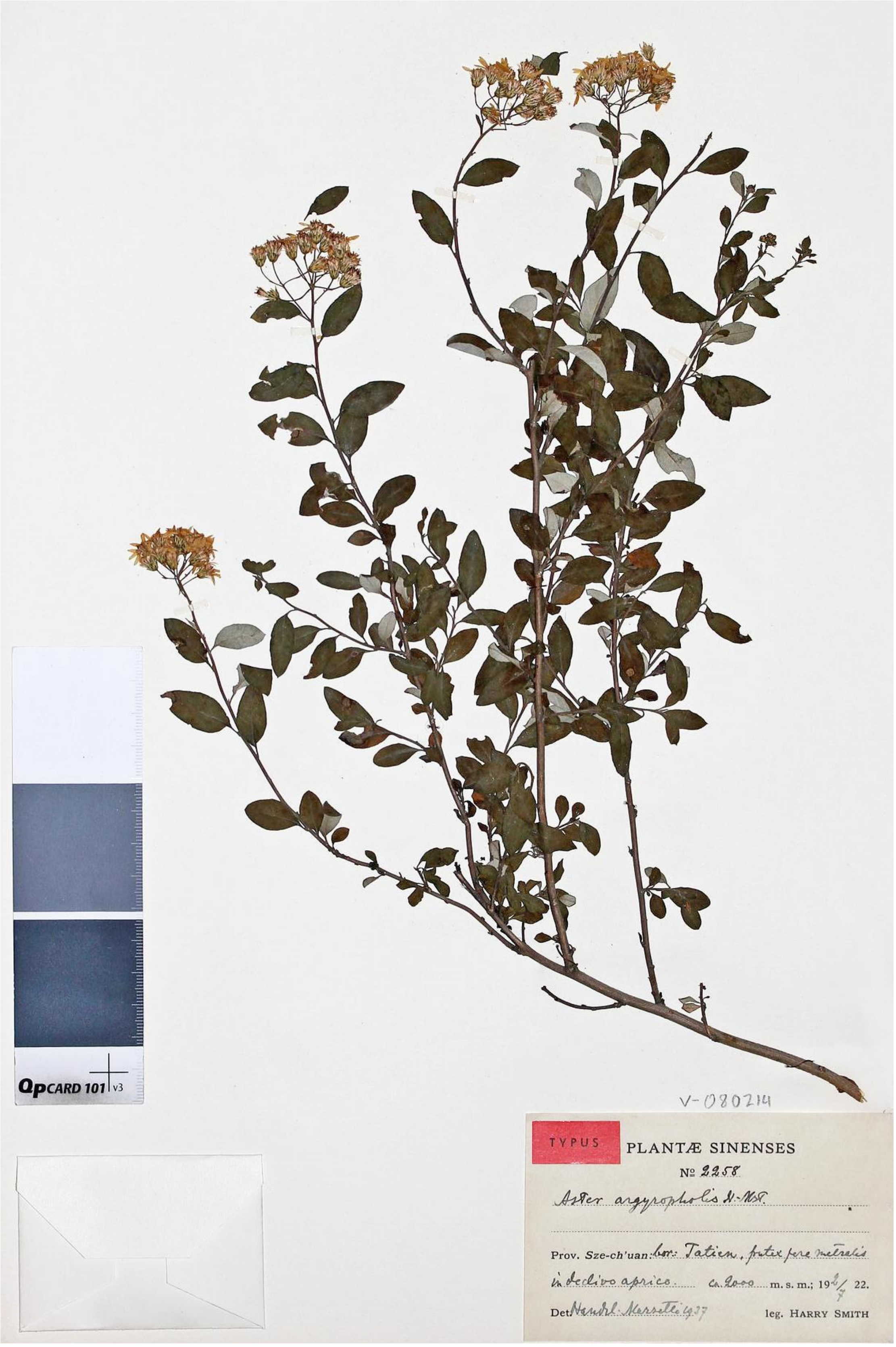

figure S25. ***Homostylium argyropholium*** (Hand.-Mazz.) Z.X.Fu, **comb. nov.** ≡ *Aster argyropholis* Hand.-Mazz. ≡ *Sinosidus argyropholis* (Hand.-Mazz.) G.L.Nesom. China, Sichuan, Kangding (Tatien-lu), frutex fere metralis in declivo aprico, 2 Jul 1922, H. Smith 2258 (holotype, UPS, v-080214!).

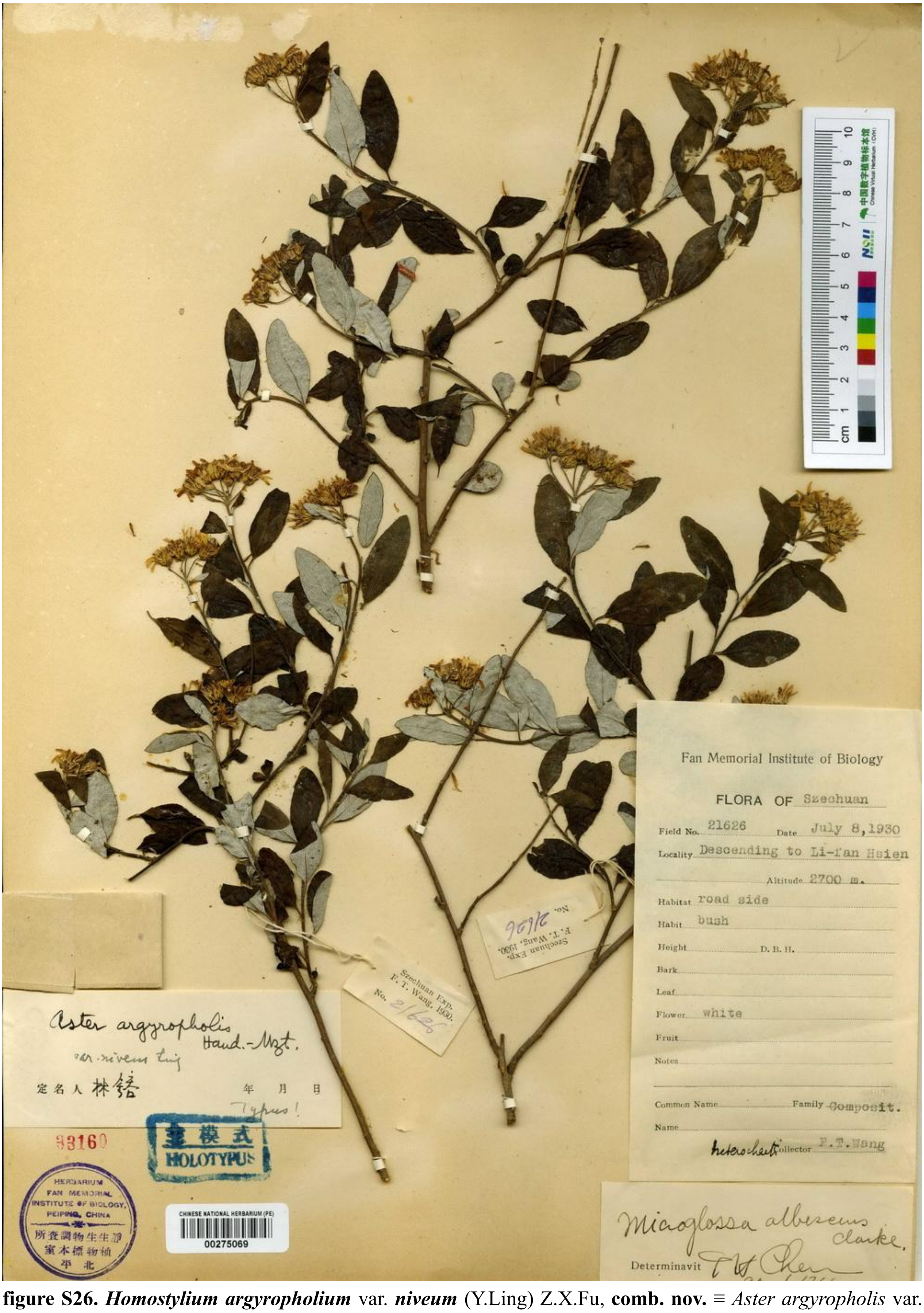

figure S26. ***Homostylium argyropholium*** var. ***niveum*** (Y.Ling) Z.X.Fu, **comb. nov.** ≡ *Aster argyropholis* var. *niveus* Y.Ling ≡ *Sinosidus argyropholis* (Hand.-Mazz.) G.L.Nesom. China, Sichuan, Lixian, alt. 2700m, 8 July 1930, F. T. Wang 21626 (holotype, PE 00275069!).

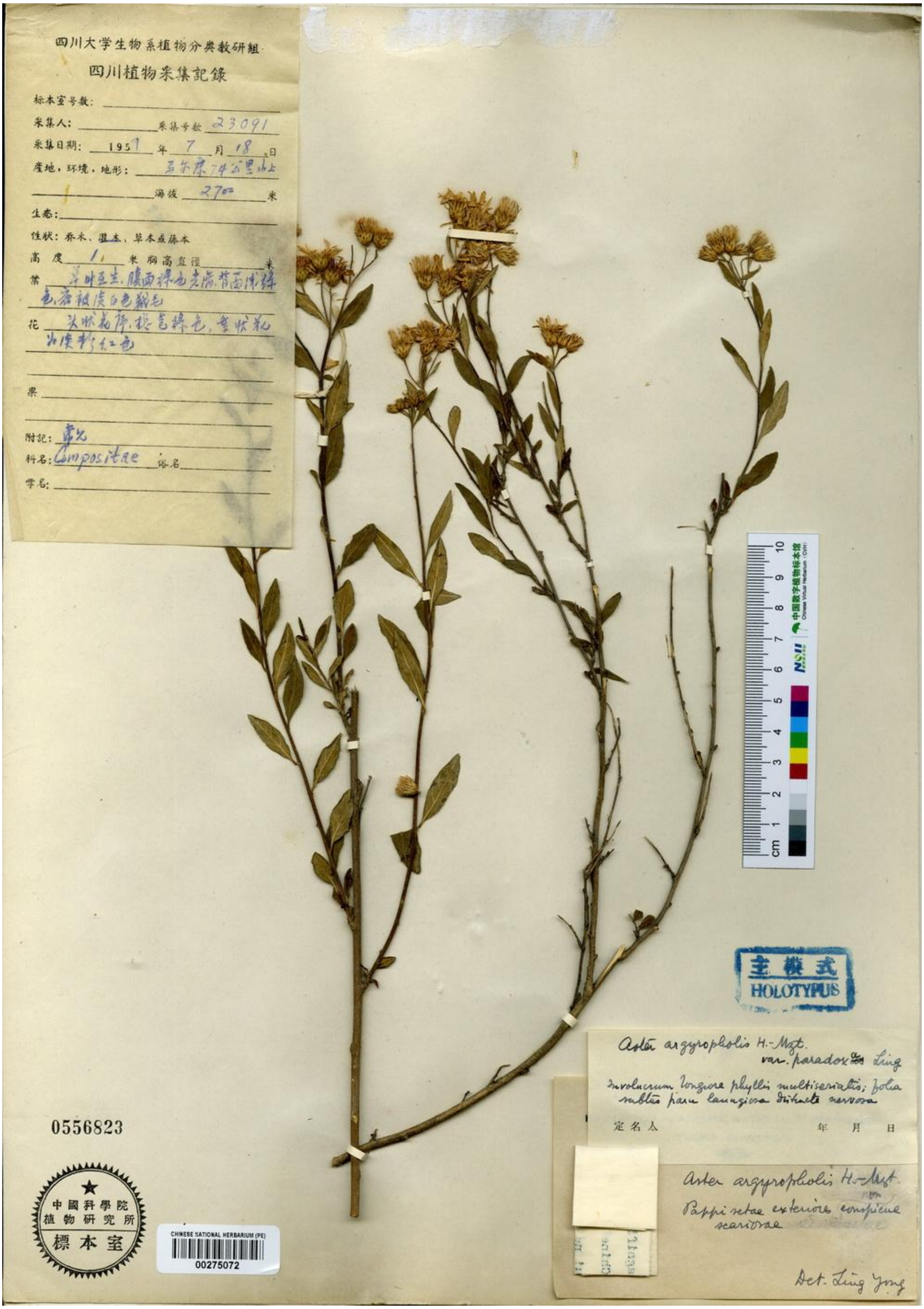

figure S27. ***Homostylium argyropholium*** var. ***paradoxum*** (Y.Ling) Z.X.Fu, **comb. nov.** ≡ *Aster argyropholis* var. *paradoxus* Y.Ling ≡ *Sinosidus paradoxus* (Y.Ling) G.L.Nesom. China, Sichuan, Barkam, alt. 2700 m, 18 July 1957, X. Li 23091 (holotype, PE 00275072!).

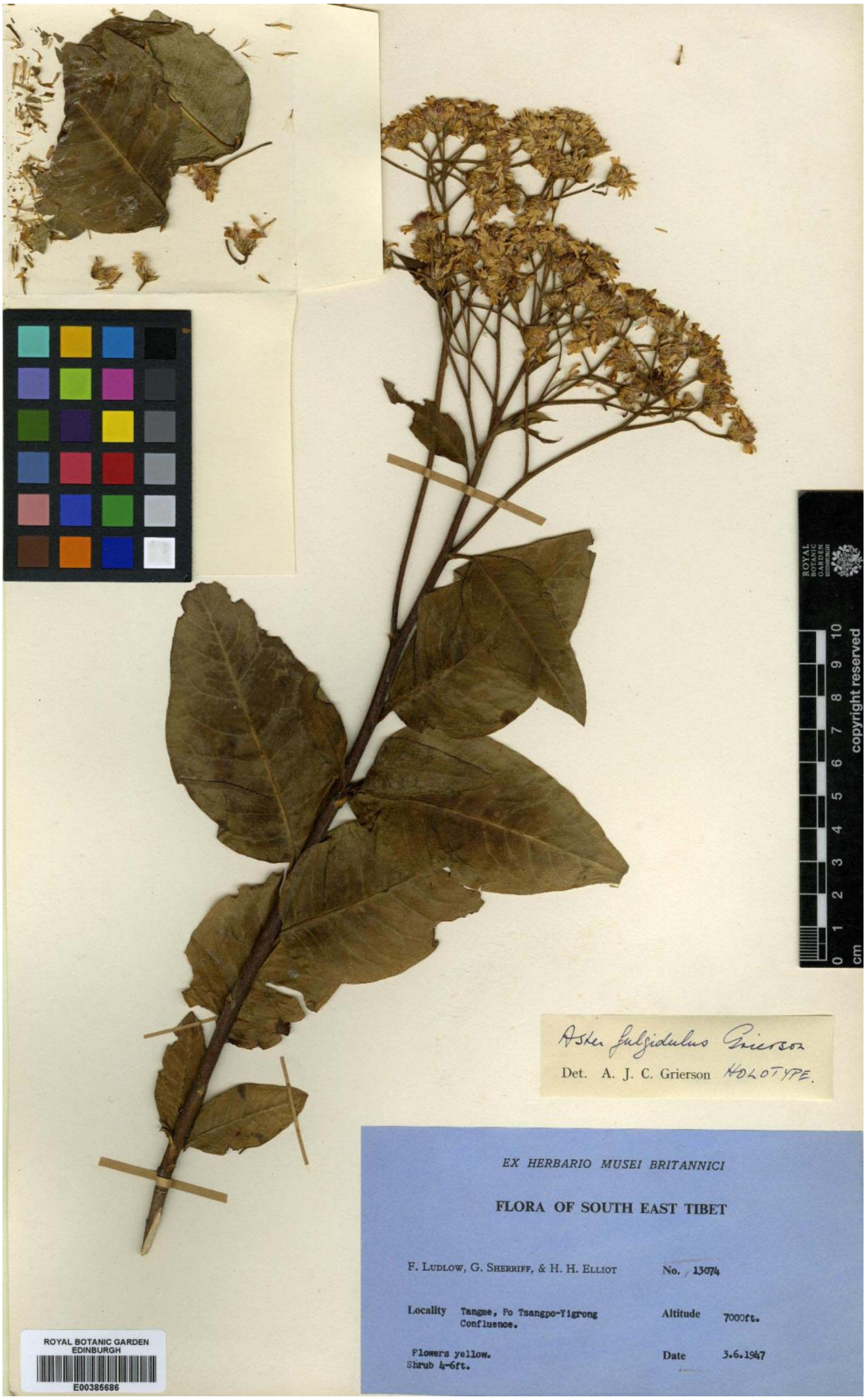

figure S28. ***Homostylium fulgidulum*** (Grierson) Z.X.Fu, **comb. nov.** ≡ *Aster fulgidulus* Griers. ≡ *Sinosidus fulgidulus* (Grierson) G.L.Nesom. China, Xizang, Bomê, Tangme (Tongmai), Tsangpo-Yigrong Confluence, alt. 7000 ft, 3 June 1947, F. Ludlow, G. Sherriff & H. H. Elliott 13074 (holotype, E 00385686!).

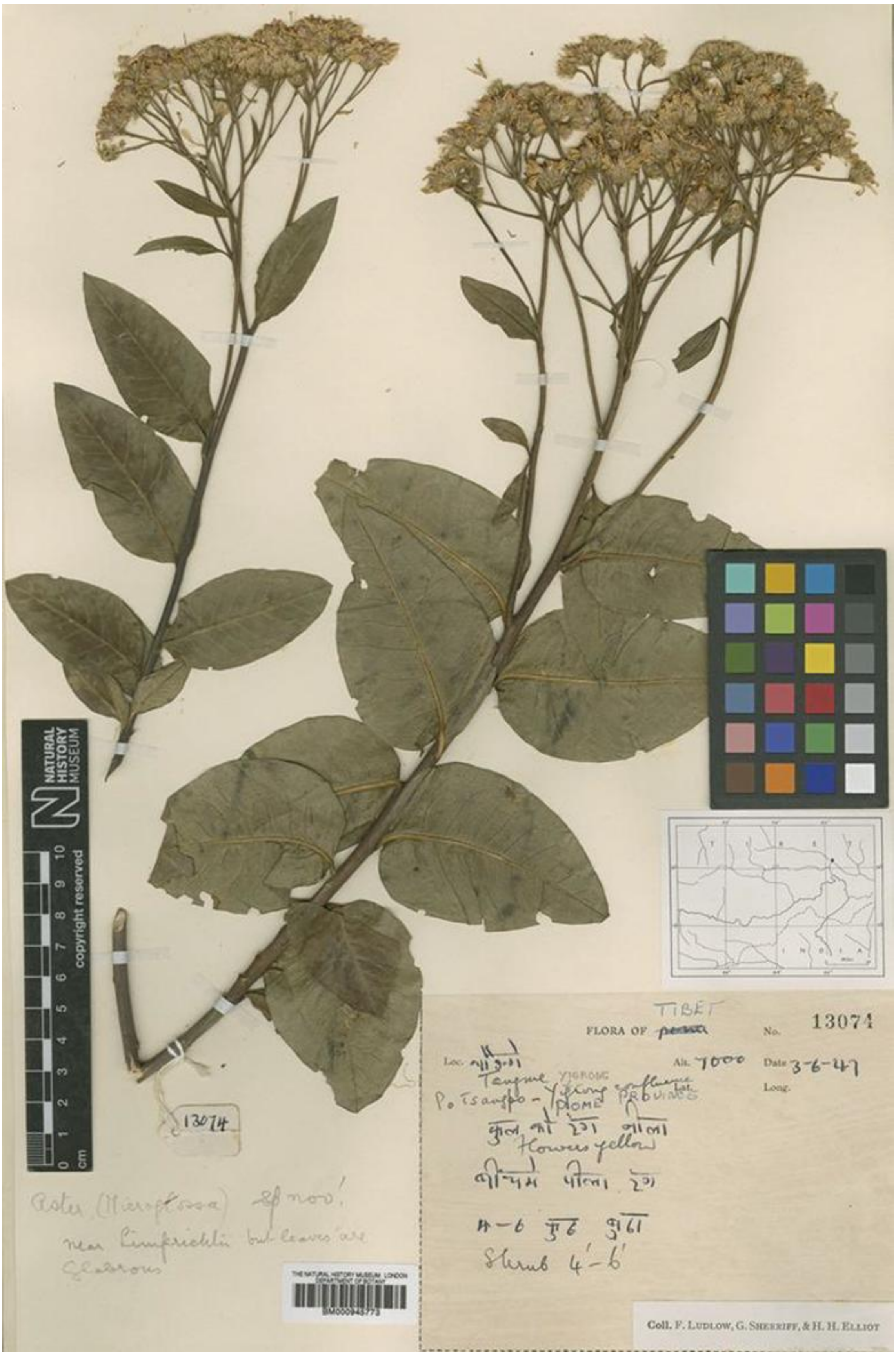

figure S29. ***Homostylium fulgidulum*** (Grierson) Z.X.Fu, **comb. nov.** ≡ *Aster fulgidulus* Griers. ≡ *Sinosidus fulgidulus* (Grierson) G.L.Nesom. China, Xizang, Bomê, Tangme (Tongmai), Tsangpo-Yigrong Confluence, alt. 7000 ft, 3 June 1947, F. Ludlow, G. Sherriff & H. H. Elliott 13074 (isotype, BM 000945773!).

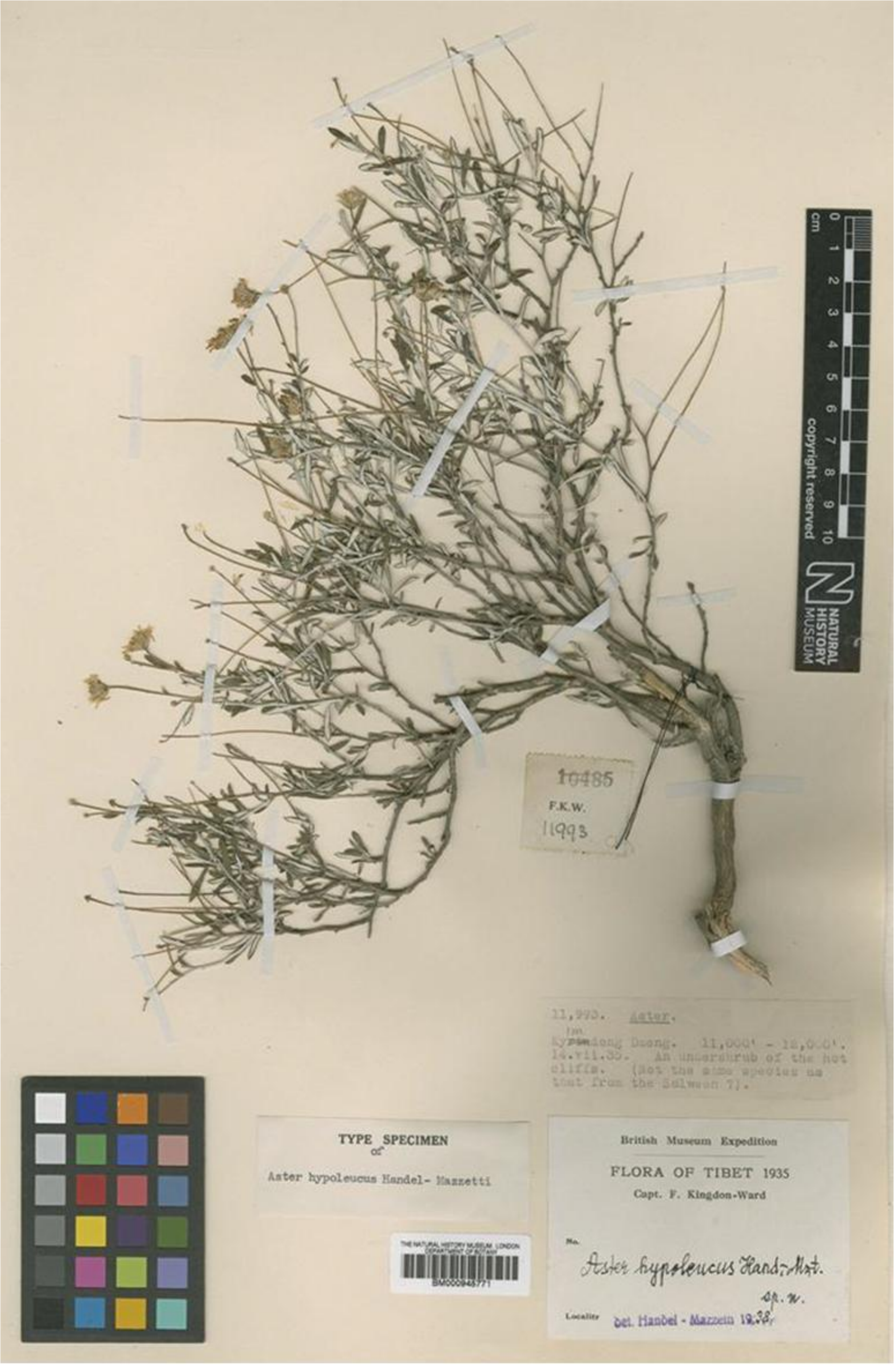

figure S30. ***Homostylium hypoleucus*** (Hand.-Mazz.) Z.X.Fu, **comb. nov.** ≡ *Aster hypoleucus* Hand.-Mazz. ≡ *Sinosidus hypoleucus* (Hand.-Mazz.) G.L.Nesom. China, Xizang, Nangxian, Kyimdong Dzong, F. Kingdon-Ward 11993 (holotype, BM 000945771!).

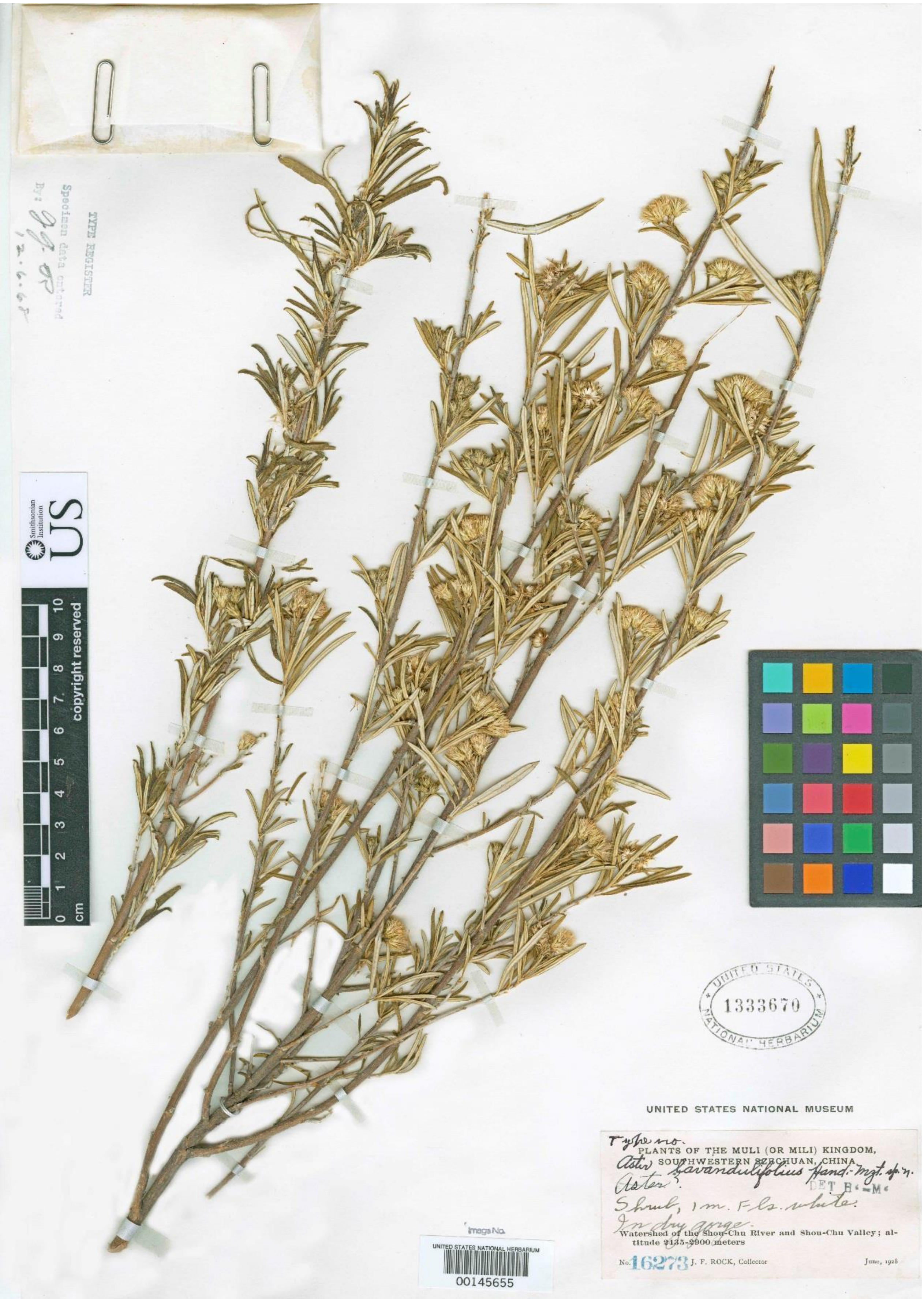

figure S31. ***Homostylium lavandulifolium*** (Hand.-Mazz.) Z.X.Fu, **comb. nov.** ≡ *Aster lavandulifolius* Hand.-Mazz. ≡ *Sinosidus lavandulifolius* (Hand.-Mazz.) G.L.Nesom. China, Sichuan, Muli, watershed of the Shou-chu river and Shou-chu valley, in dry gorge, alt. 2435-2900 m, June 1928, J.C.F. Rock 16273 (isolectotype, US 00145655!).

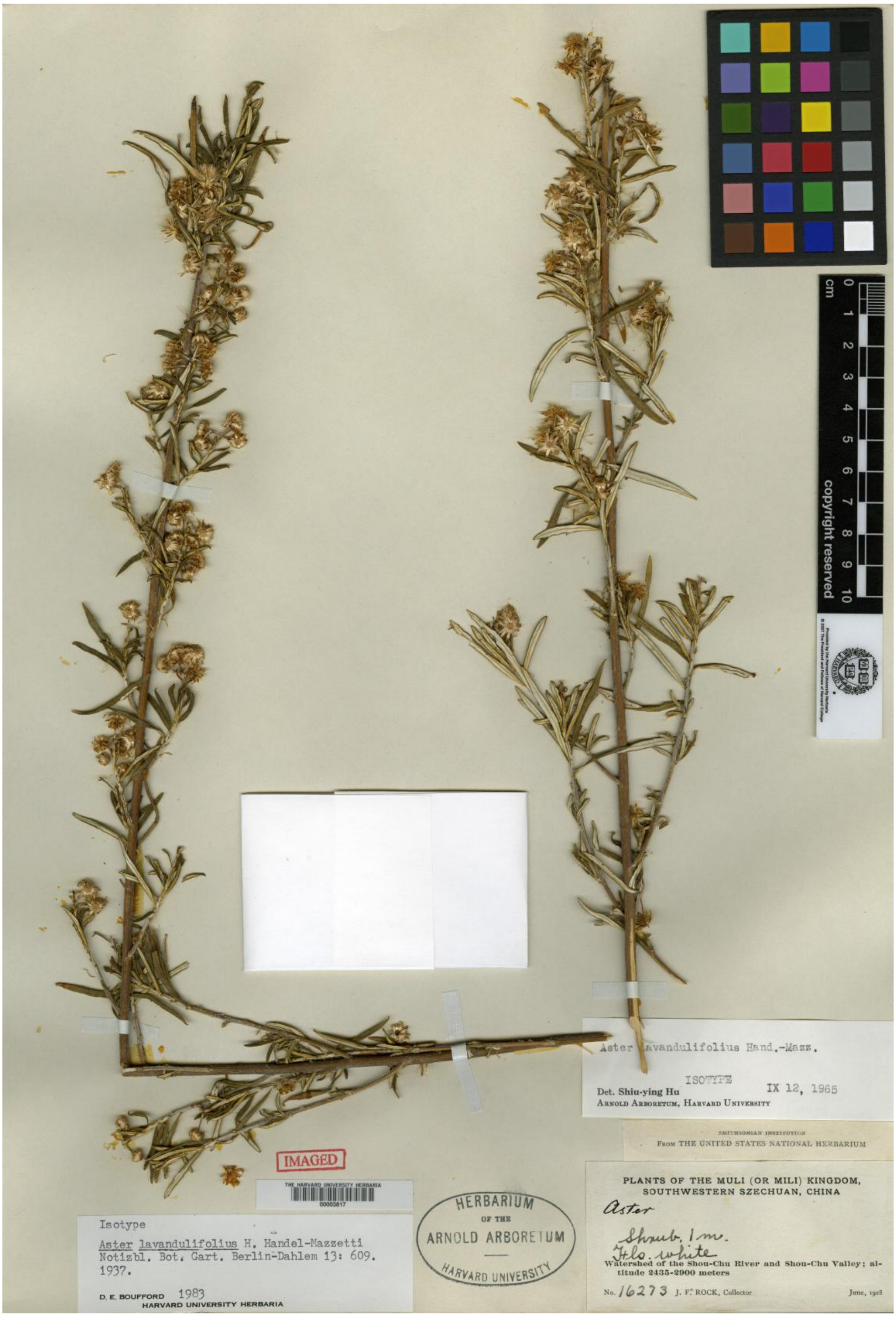

figure S32. ***Homostylium lavandulifolium*** (Hand.-Mazz.) Z.X.Fu, **comb. nov.** ≡ *Aster lavandulifolius* Hand.-Mazz. ≡ *Sinosidus lavandulifolius* (Hand.-Mazz.) G.L.Nesom. China, Sichuan, Muli, watershed of the Shou-chu river and Shou-chu valley, in dry gorge, alt. 2435-2900 m, June 1928, J.C.F. Rock 16273 (isolectotype, A 00003817!).

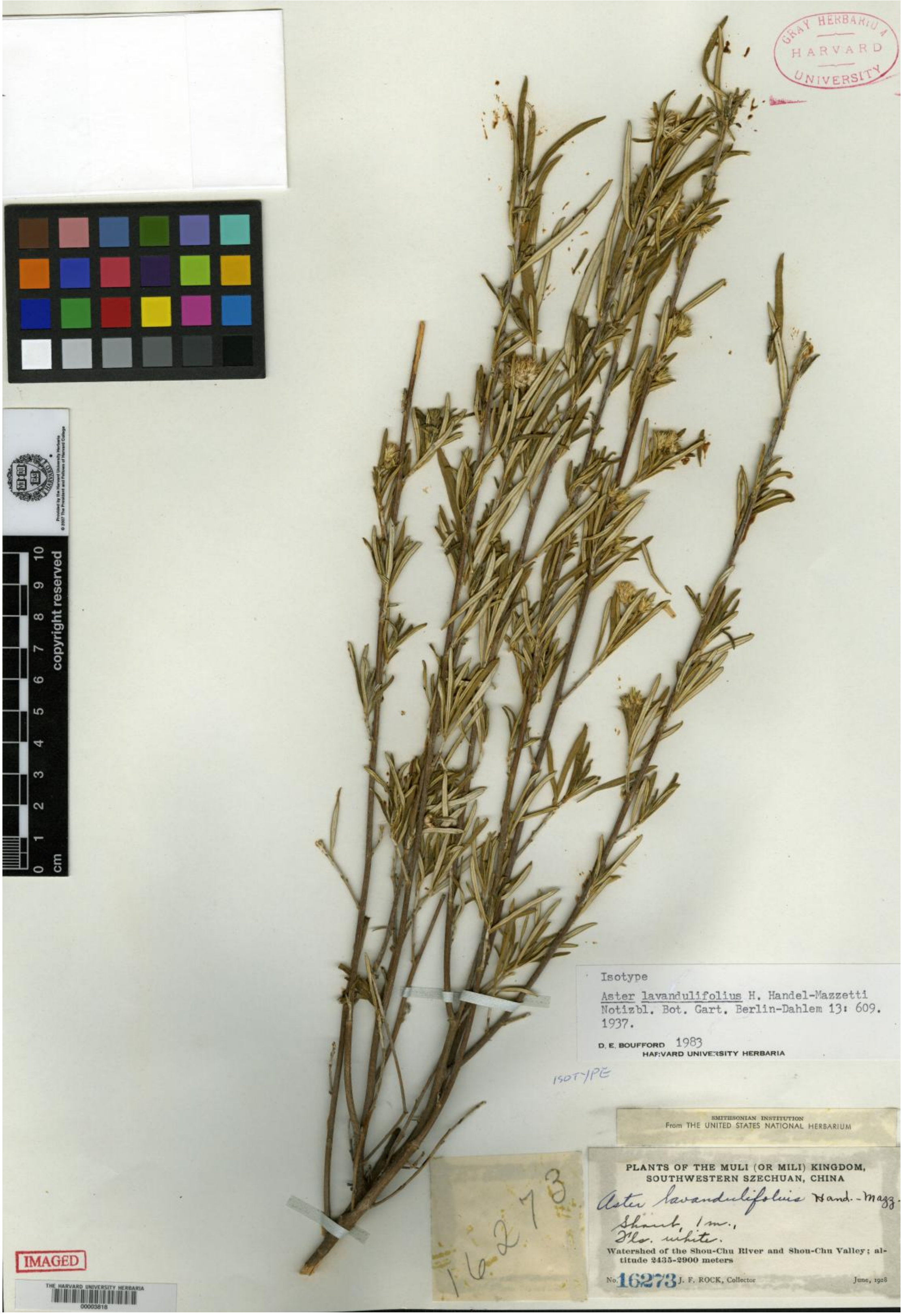

figure S33. ***Homostylium lavandulifolium*** (Hand.-Mazz.) Z.X.Fu, **comb. nov.** ≡ *Aster lavandulifolius* Hand.-Mazz. ≡ *Sinosidus lavandulifolius* (Hand.-Mazz.) G.L.Nesom. China, Sichuan, Muli, watershed of the Shou-chu river and Shou-chu valley, in dry gorge, alt. 2435-2900 m, June 1928, J.C.F. Rock 16273 (isolectotype, GH 00003818!).

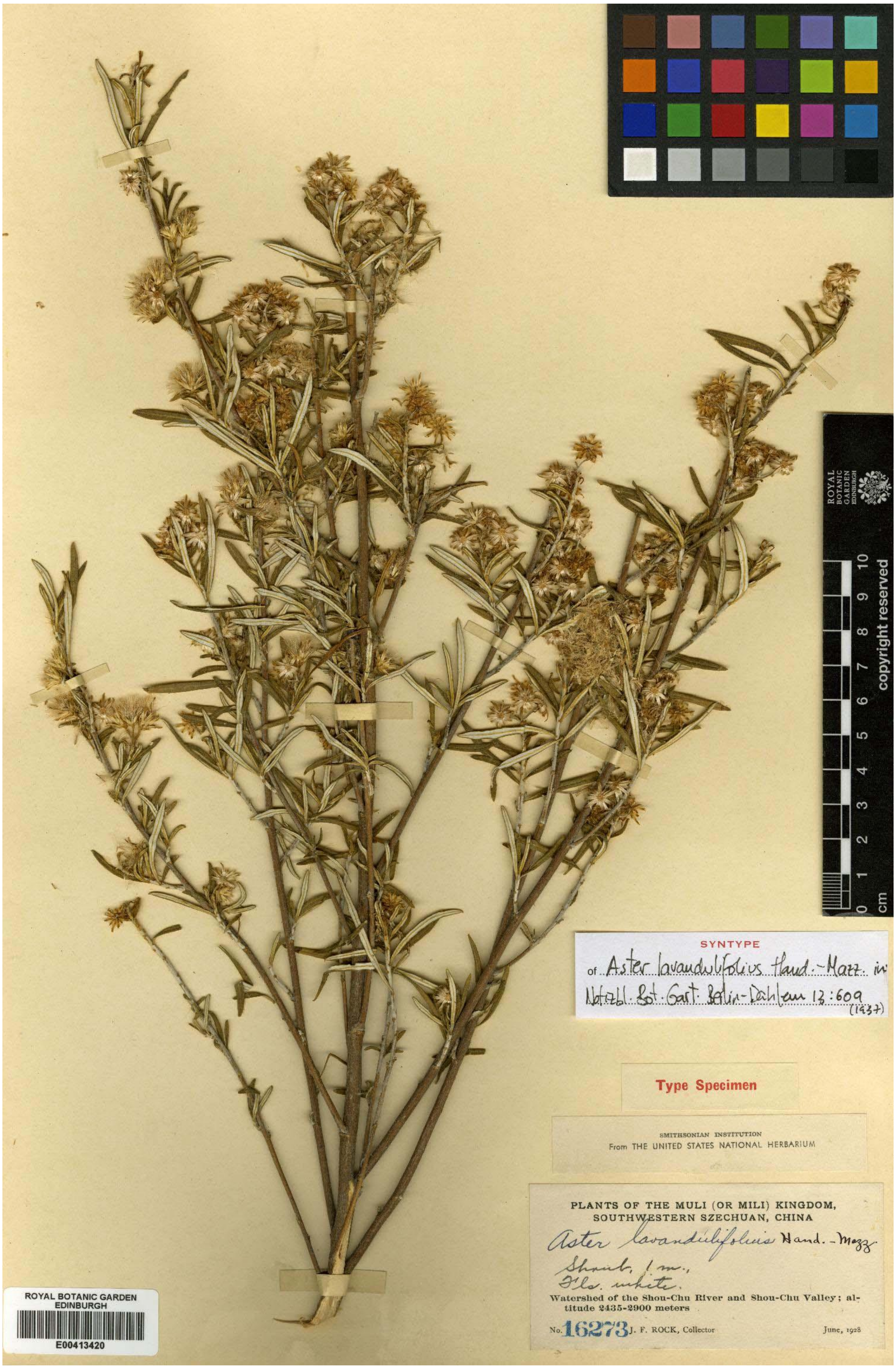

figure S34. ***Homostylium lavandulifolium*** (Hand.-Mazz.) Z.X.Fu, **comb. nov.** ≡ *Aster lavandulifolius* Hand.-Mazz. ≡ *Sinosidus lavandulifolius* (Hand.-Mazz.) G.L.Nesom. China, Sichuan, Muli, watershed of the Shou-chu river and Shou-chu valley, in dry gorge, alt. 2435-2900 m, June 1928, J.C.F. Rock 16273 (isolectotype, E 00413420!).

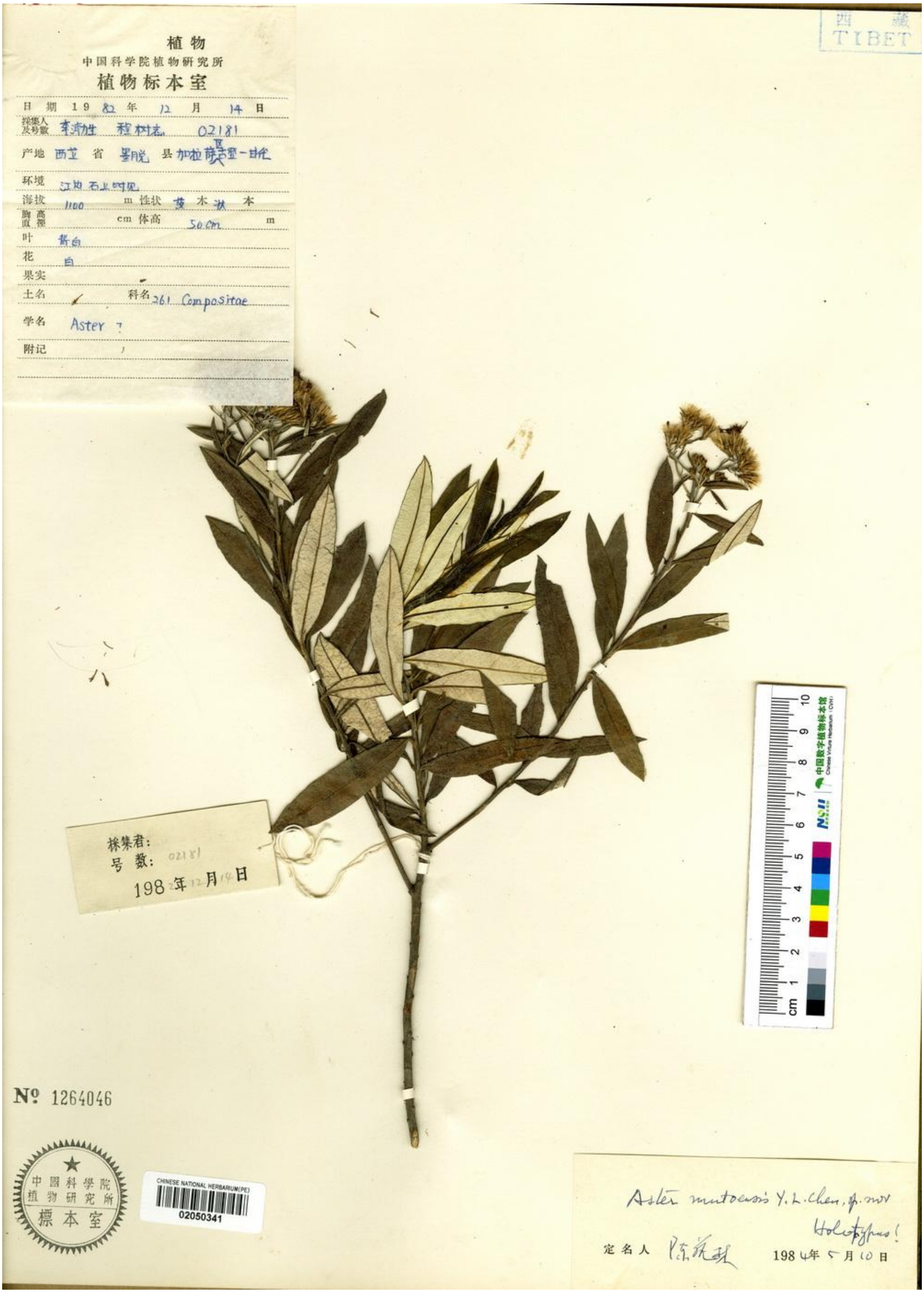

figure S35. ***Homostylium motuoense*** (Y.L.Chen) Z.X.Fu, **comb. nov.** ≡ *Aster motuoensis* Y.L.Chen ≡ *Sinosidus motuoensis* (Y.L.Chen) G.L.Nesom. China, Xizang, Mêdog, Qarasa, gudeng-ganhua, in prato lapidoso secus marginem rivuli, alt. 1100 m, Dec. 1982, S. Z. Cheng & B. S. Li 2181 (holotype, PE 02050341!).

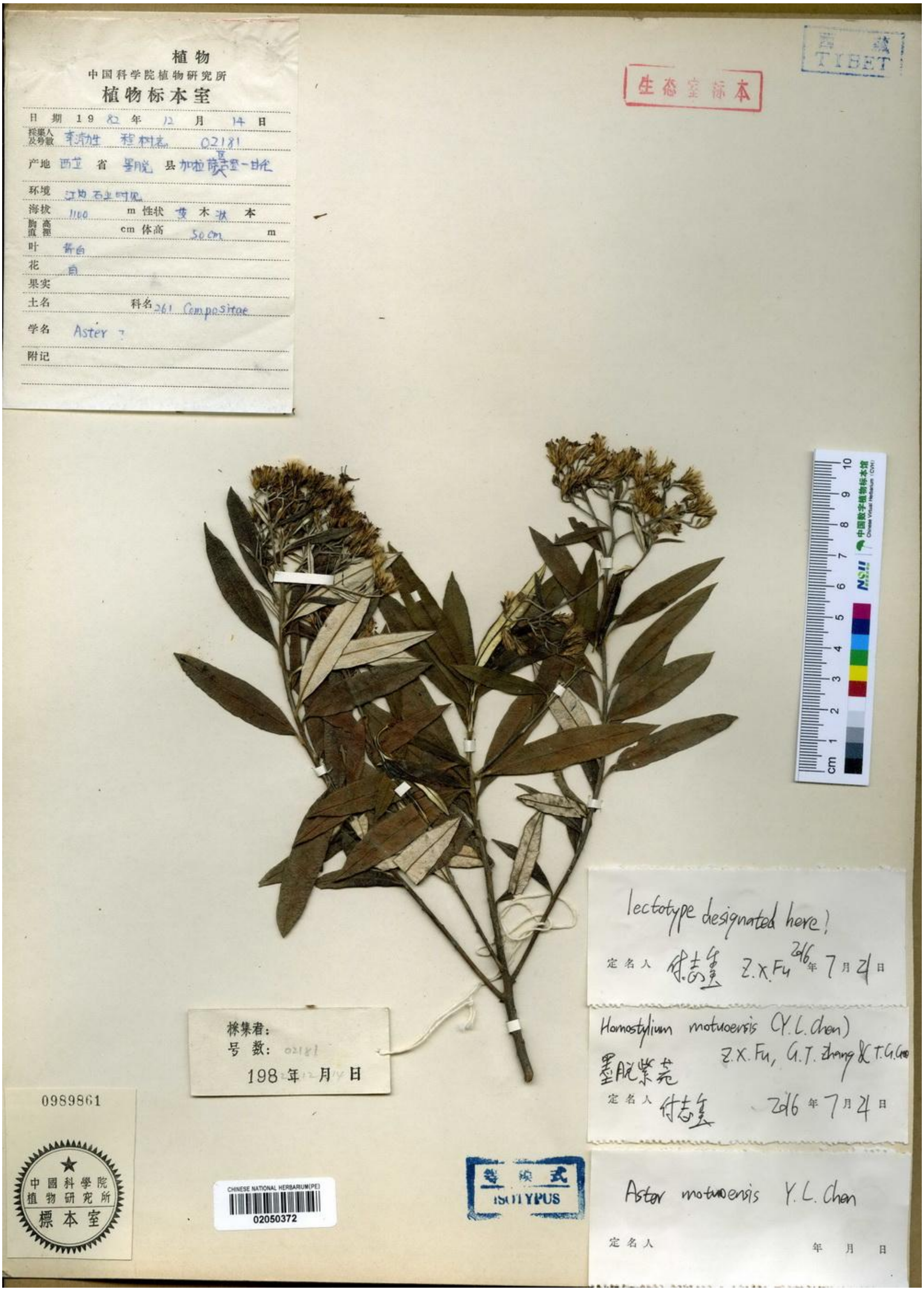

figure S36. ***Homostylium motuoense*** (Y.L.Chen) Z.X.Fu, **comb. nov.** ≡ *Aster motuoensis* Y.L.Chen ≡ *Sinosidus motuoensis* (Y.L.Chen) G.L.Nesom. China, Xizang, Mêdog, Qarasa, gudeng-ganhua, in prato lapidoso secus marginem rivuli, alt. 1100 m, Dec. 1982, S. Z. Cheng & B. S. Li 2181 (isotypes, PE 02050372!).

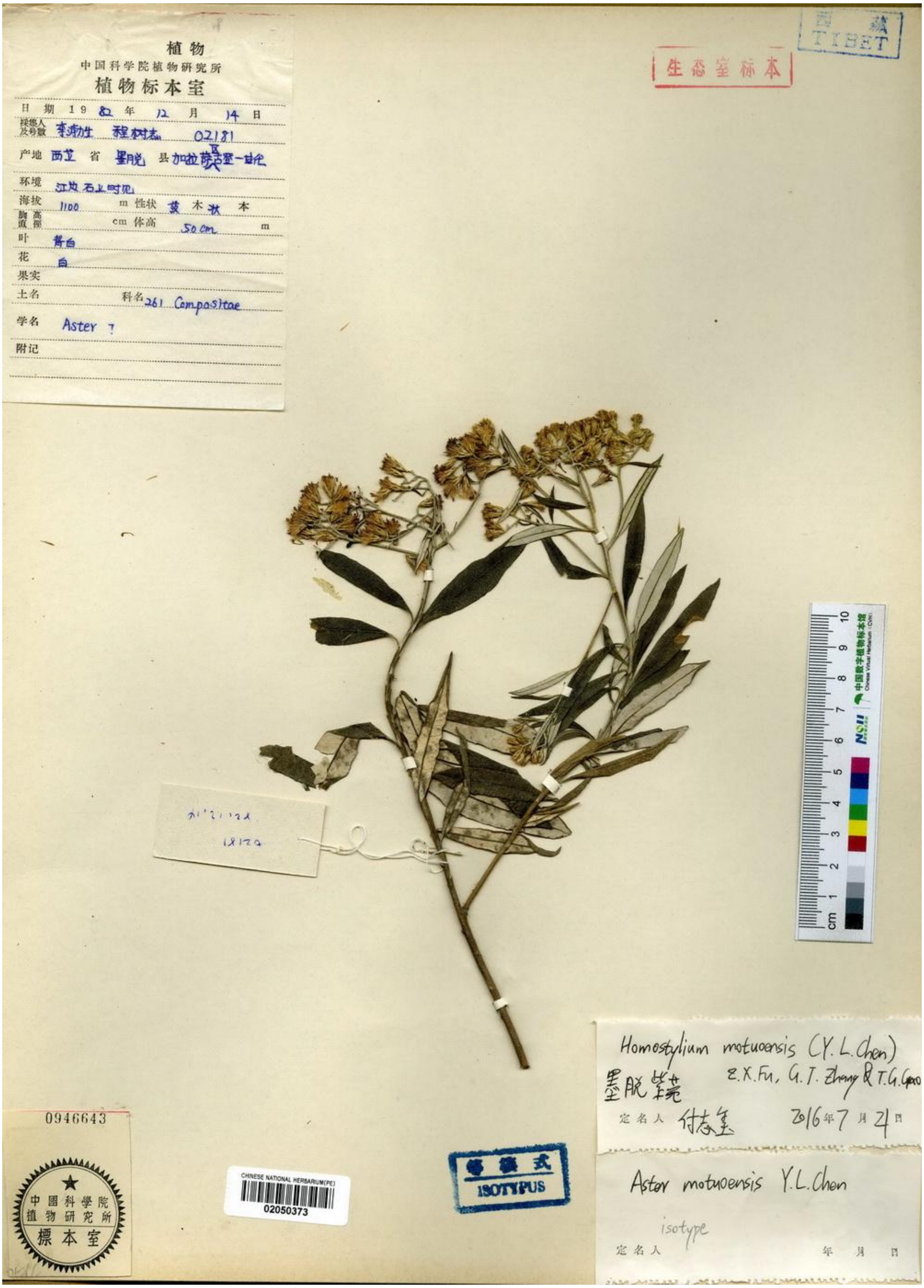

figure S37. ***Homostylium motuoense*** (Y.L.Chen) Z.X.Fu, **comb. nov.** ≡ *Aster motuoensis* Y.L.Chen ≡ *Sinosidus motuoensis* (Y.L.Chen) G.L.Nesom. China, Xizang, Mêdog, Qarasa, gudeng-ganhua, in prato lapidoso secus marginem rivuli, alt. 1100 m, Dec. 1982, S. Z. Cheng & B. S. Li 2181 (isotype, PE 02050373!).

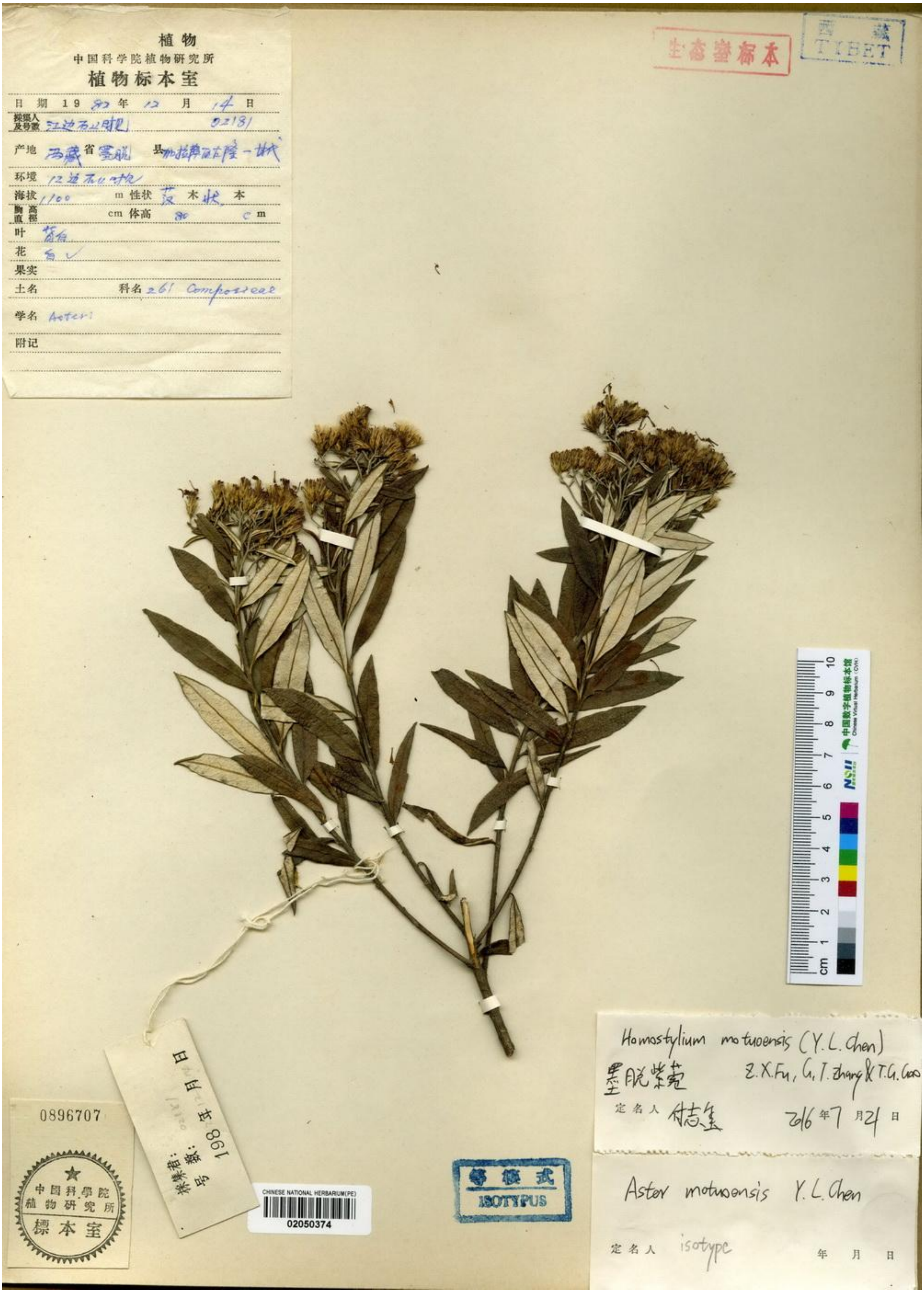

figure S38. ***Homostylium motuoense*** (Y.L.Chen) Z.X.Fu, **comb. nov.** ≡ *Aster motuoensis* Y.L.Chen ≡ *Sinosidus motuoensis* (Y.L.Chen) G.L.Nesom. China, Xizang, Mêdog, Qarasa, gudeng-ganhua, in prato lapidoso secus marginem rivuli, alt. 1100 m, Dec. 1982, S. Z. Cheng & B. S. Li 2181 (isotype, PE 02050374!).

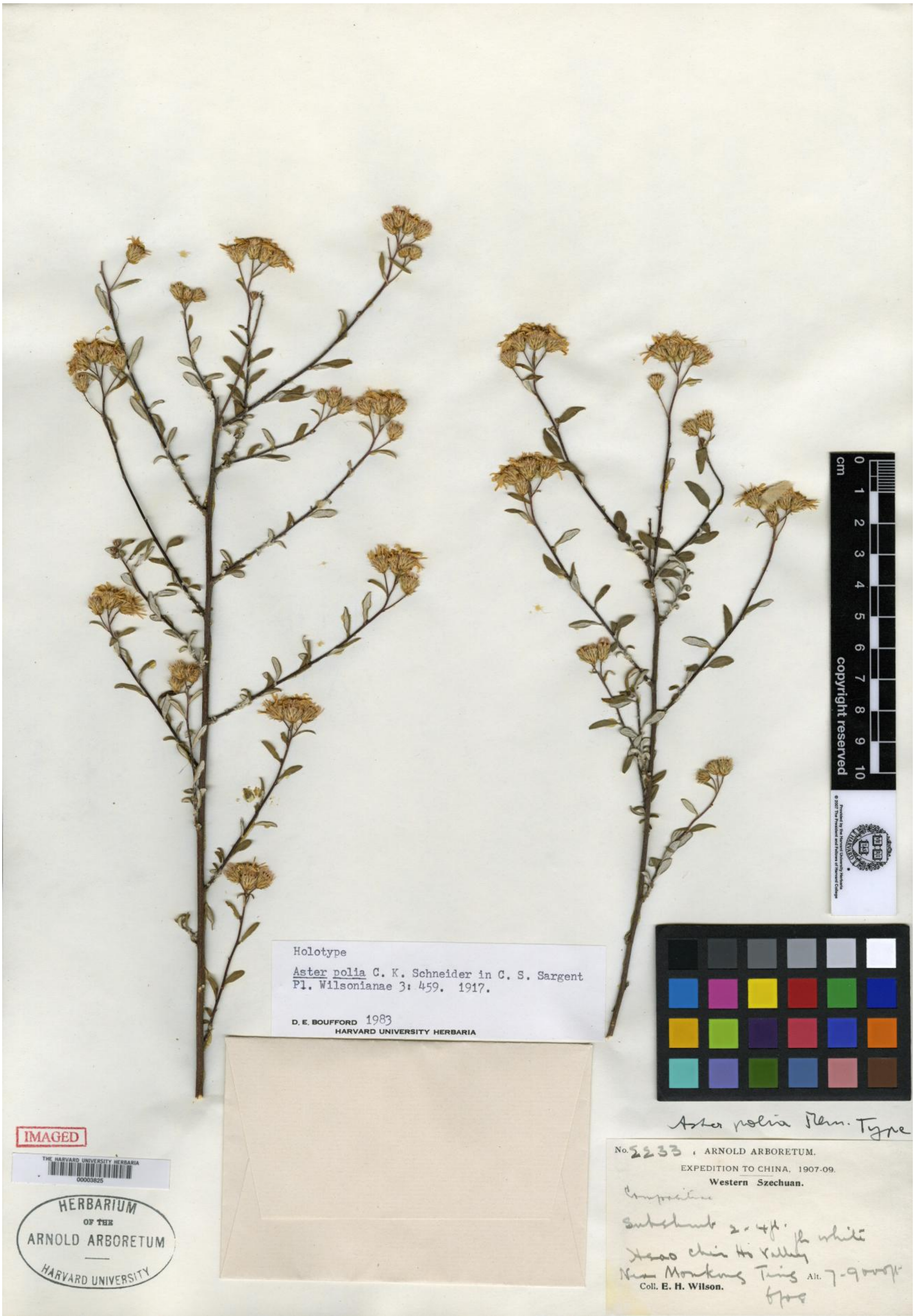

figure S39. ***Homostylium polium*** (Schneid.) Z.X.Fu, **comb. nov.** ≡ *Aster polius* C.K.Schneid. ≡ *Sinosidus polius* (C.K.Schneid.) G.L.Nesom. China, Sichuan, Xiaojin (=Nin Monkong Ting), head of Chin Ho Valley, alt. 7000-9000 ft, June 1908, E. H. Wilson 2233 (holotype, A 00003825!).

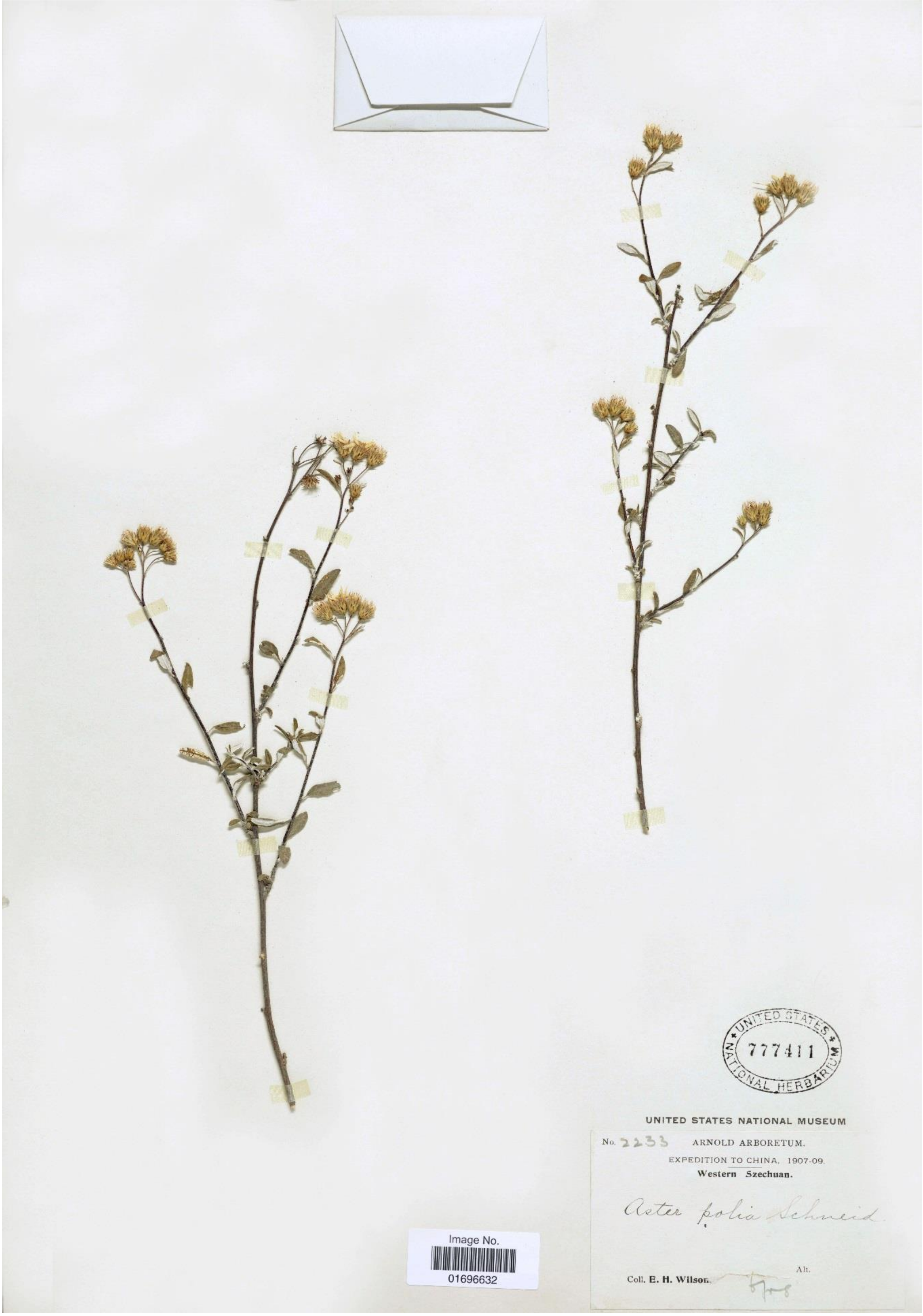

figure S40. *Homostylium polium* (Schneid.) Z.X.Fu, **comb. nov.** ≡ *Aster polius* C.K.Schneid. ≡ *Sinosidus polius* (C.K.Schneid.) G.L.Nesom. China, Sichuan, Xiaojin (=Nin Monkong Ting), head of Chin Ho Valley, alt. 7000-9000 ft, June 1908, E. H. Wilson 2233 (isotype, US 01696632!).
